# Evolution of increased longevity and slowed ageing in a genus of tropical butterfly

**DOI:** 10.1038/s41467-026-73635-7

**Published:** 2026-06-16

**Authors:** Jessica Foley, Josie McPherson, Made Roger, Cruz Batista, Rémi Mauxion, Greta Hernández, Richard Kelson, Fletcher J. Young, W. Owen McMillan, Stephen H. Montgomery

**Affiliations:** 1 School of Biological Sciences, University of Bristol, Bristol, UK; 2 Jean Mayer USDA Human Nutrition Research Center on Aging, Tufts University, Boston, MA USA; 3 Smithsonian Tropical Research Institute, Gamboa, Colón Panama; 4 Butterfly Habitat, Six Flags Discovery Kingdom, Vallejo, CA USA; 5 Department of Zoology, University of Cambridge, Cambridge, UK

**Keywords:** Evolution, Animal physiology, Evolutionary ecology, Entomology

## Abstract

Evolution has given rise to lifespans in extant species ranging from days to centuries. Given that mechanisms of ageing are highly conserved, studying long-lived lineages across the animal kingdom could yield insights relevant for healthy ageing in humans. The long lifespans reported for the *Heliconius* butterfly genus position it as a promising new model system for such studies, but its potential is limited by a paucity of available data. Here, we collate data from commercial butterfly houses, mark-release-recapture studies, and insectary populations to reveal the evolution of a three-fold lifespan extension in *Heliconius* over their close relatives in the Heliconiini tribe, with maximum lifespans in some species stretching nearly up to a year. We further demonstrate through captive experiments that this lifespan extension is accompanied by slowed actuarial and physiological ageing. Together, these results establish *Heliconius* as a powerful model for investigating the evolutionary and mechanistic basis of increased longevity.

## Abstract (web summary)

Studying long-lived species across the animal kingdom could provide insights into healthy ageing in humans. This study reveals long lifespans and slowed ageing in *Heliconius* butterflies, identifying them as a valuable new model for ageing biology.

## Introduction

The diversity of lifespans across the animal kingdom, ranging from days to centuries^1^, has intrigued scholars for millennia^2^. More recent work on age-specific mortality has now shown that variation in rates of ageing is just as pronounced; while humans show steep increases in mortality with age, for many species these inclines are much gentler^3^. Evolutionary explanations for senescence, defined as the physiological deterioration that accompanies ageing, rest on the concept of the “selection shadow”, the declining force of natural selection with age^4,5^. This effect is thought to provide an explanation for the prevalence of age-related declines in physiological function and survival. Yet the observed diversity in ageing across the tree of life^3^ suggests that many organisms have evolved adaptations allowing them to slow or delay senescence. Considering the high evolutionary conservation of mechanisms of ageing^6^, uncovering such adaptations across diverse taxa may provide unique insights for the study of healthy ageing.

As the most species-rich animal class, insects are famed for their morphological and ecological diversity. They also display extreme variation in longevity, with maximum lifespans ranging from just a few days in adult mayflies to up to several decades in the case of some ant and termite reproductive castes. This represents a 5000-fold difference within the class – versus, for comparison, the 100-fold difference in lifespan observed in mammals^7^. The majority of research on insect ageing has thus far focused on intra-species differences, in particular in the fruit fly, *Drosophila melanogaster*, where the vast genetic toolbox has allowed for the elucidation of specific genes regulating lifespan^8^. Similarly, research on the dramatic differences in lifespan between social insect castes has provided valuable insights into the molecular underpinnings of such plasticity^9^. This work has been instrumental in uncovering the proximate mechanisms of lifespan extension. However, leveraging the rich inter-specific diversity in insect lifespan, in combination with the depth of available ecological knowledge, can help to illuminate the evolutionary origins of lengthened life. In particular, inter-species lifespan differences within insects can be long in relative terms but short in absolute terms, making them much more tractable for longitudinal studies than typical mammalian and avian models of extended longevity, which often live for decades.

The long adult lifespans of the Neotropical *Heliconius* genus rank among the longest recorded in butterflies^10^, and have been reported to stretch to at least 6 months in the wild^11^. While some other Lepidoptera display lifespans on similar scales^12^, or even longer when accounting for diapause^13^, *Heliconius*’ longevity represents a dramatic adult lifespan extension over the ~6 weeks reported for their close relatives in the Heliconiini tribe^14^. The relatively recent evolutionary origin (18mya) of *Heliconius’* lifespan extension offers an ideal comparative framework for pinpointing the basis of extended longevity^15^. The remarkable lifespan increase in *Heliconius* has been linked to the evolution of adult pollen-feeding in this genus, which is unique among butterflies^14,16^. Pollen-feeding provides *Heliconius* with a source of amino acids in adulthood, in contrast with their Heliconiini relatives, which, as adults, must rely on nitrogenous resources derived from larval phytophagy^16,17^. All *Heliconius* species actively collect and ingest pollen, with the exception of one clade of 4 species, whose phylogenetic position has been debated^15,16^. This dietary innovation has been linked to a suite of other behavioural, neuroanatomical, and physiological traits in *Heliconius* that are not present in other non-pollen-feeding Heliconiini^16^. These include increased investment in neural structures supporting learning and memory^18^ and more stable long-term visual memories^19^. Importantly, pollen-derived amino acids have been shown to facilitate a lengthened reproductive lifespan in female *Heliconius*, enabling the continual production of oocytes throughout adulthood^14,20^. While pollen-feeding is also linked to lower daily oviposition rates^14^, their prolonged reproductive capacity results in greater overall lifetime fecundity compared with pollen-deprived *Heliconius* and their non-pollen-feeding Heliconiini relatives, which exhibit rapid reproductive senescence beginning at approximately three weeks of age^14^. Pollen consumption has also been suggested to directly extend *Heliconius’* lifespan, with pollen-deprived *Heliconius* reportedly living no longer than their non-pollen-feeding relatives^14^. However, the evidence for a direct effect on longevity is more equivocal, and more recent pollen-deprivation experiments have not recapitulated these results^20,21^. Across evolutionary time, enhanced nutrition may have instead indirectly facilitated longevity in this genus via an investment in longer-lived tissues. The lengthened reproductive lifespan permitted by this nutritional advantage would likely favour selection for longevity due to the enhanced fitness of long-lived, continually reproducing individuals.

Despite *Heliconius’* potential as a model for the evolution of longevity, available data are restricted to scattered observations of maximum lifespan. These data point to significant increases in *Heliconius* over their non-pollen-feeding Heliconiini relatives (see Table 1). However, discussion of this lifespan extension tends to refer to a single broad shift in *Heliconius*, despite observed variation in the degree of this increase between *Heliconius* species. Maximum lifespan is also a notoriously problematic metric of ageing, correlating highly with sample size and being unrepresentative of an average individual^22^. More thorough survival data from a wider range of species across the tribe would allow for estimates of more reliable metrics, such as median lifespan, which are less sensitive to sample size^23^. Parametric survival analysis, which fits survival data to a hazard function to understand mortality patterns over time, can also help to disentangle the basis of observed lifespan differences by offering insights into how not just survival, but also rates of ageing, vary between populations^24^. These techniques allow for the identification and quantification of actuarial senescence, defined as an increase in mortality with age. This is distinct from physiological senescence, which measures the decline in physiological function that may often accompany the ageing process, although actuarial senescence may imply the presence of physiological senescence^25^. A characterisation of physiological senescence in this system would also allow for a thorough interrogation of the direct and indirect impacts of pollen-feeding on *Heliconius* life history, and provide a reliable index upon which future ageing-related interventions may be based. To date, the only description of physiological senescence in the Heliconiini tribe is limited to the reproductive senescence demonstrated by *Dryas iulia* and pollen-deprived *Heliconius*^14,21^. To clarify *Heliconius’* utility as a model system for the evolutionary and mechanistic bases of ageing, it is important to determine whether longevity in this genus reflects a simple plastic nutritional advantage or is instead indicative of evolved, genetically determined traits which may offer wider-ranging insights into longevity.

**Table 1:** Maximum reported lifespans for species in the Heliconiini tribe

Species	Feeding habit	Maximum lifespan (days)	Data source
*Heliconius hewitsoni*	pollen-feeding	348	Butterfly house (Kelson, unpublished)
*Heliconius hecale*	pollen-feeding	277	Butterfly house (Kelson, unpublished)
*Heliconius erato*	pollen-feeding	274	Butterfly house (Kelson, unpublished)
*Heliconius melpomene*	pollen-feeding	260	Butterfly house (Kelson, unpublished)
*Heliconius ismenius*	pollen-feeding	245	Butterfly house (Kelson, unpublished)
*Heliconius cydno*	pollen-feeding	244	Butterfly house (Kelson, unpublished)
*Heliconius atthis*	pollen-feeding	210	Butterfly house (Kelson, unpublished)
*Heliconius numata*	pollen-feeding	210	Butterfly house (Kelson, unpublished)
*Heliconius hortense*	pollen-feeding	198	Butterfly house (Kelson, unpublished)
*Heliconius charithonia*	pollen-feeding	184	Butterfly house (Kelson, unpublished)
*Heliconius sapho*	pollen-feeding	177	Butterfly house (Kelson, unpublished)
*Heliconius sara*	pollen-feeding	170	Butterfly house (Kelson, unpublished)
*Heliconius ethilla*	pollen-feeding	162	Mark-release-recapture^11^
*Heliconius clysonymus*	pollen-feeding	156	Butterfly house (Kelson, unpublished)
*Heliconius himera*	pollen-feeding	155	Butterfly house (Kelson, unpublished)
*Heliconius hermathena*	pollen-feeding	139	Mark-release-recapture^66^
*Heliconius pachinus*	pollen-feeding	106	Butterfly house^67^
*Dryas iulia*	non-pollen-feeding	98	Butterfly house (Kelson, unpublished)
*Heliconius hecalesia*	pollen-feeding	89	Butterfly house (Kelson, unpublished)
*Heliconius eleuchia*	pollen-feeding	86	Butterfly house (Kelson, unpublished)
*Dryadula phaetusa*	non-pollen-feeding	85	Butterfly house (Kelson, unpublished)
*Heliconius nattereri*	pollen-feeding	77	Mark-release-recapture^68^
*Heliconius doris*	pollen-feeding	69	Butterfly house (Kelson, unpublished)
*Heliconius xanthocles*	pollen-feeding	66	Mark-release-recapture^69^
*Agraulis vanillae*	non-pollen-feeding	60	Butterfly house^67^
*Eueides isabella*	non-pollen-feeding	46	Butterfly house (Kelson, unpublished)
*Philaethria dido*	non-pollen-feeding	44	Butterfly house^67^
*Dione juno*	non-pollen-feeding	14	Insectary population^70^

“Mark-release-recapture” refers to studies conducted on wild populations; “insectary population” refers to studies conducted on captive populations reared in insectary conditions (typically in smaller cages); “butterfly house” refers to studies conducted on captive populations living in semi-natural conditions in commercial butterfly houses. The single highest record for each species is shown; for additional records, see Supplementary Data 2.

Here, we leverage the neat experimental framework provided by comparisons with the closely-related but shorter-lived Heliconiini outgroups to assess the degree of *Heliconius’* lifespan extension, the extent to which it is accompanied by slowed actuarial and functional senescence, and its evolutionary association with their unique pollen-feeding behaviour. We collate data from multiple complementary sources to build a profile of ageing across the Heliconiini tribe, broadly supporting a shift towards longer lifespans co-occurring with the transition to a pollen-feeding behaviour, but revealing diversity in the parameters of ageing within the *Heliconius* genus. We then narrow our focus to one representative long-lived pollen-feeder, *Heliconius hecale*, and one representative short-lived non-pollen-feeder, *Dryas iulia*, combining survival analyses and measurements of physiological senescence across the lifespan under both pollen-fed and pollen-deprived diet treatments to disentangle the impact of diet on *Heliconius* ageing and longevity. Our results provide evidence for an evolved lifespan extension and slowed ageing in this genus, and establish *Heliconius* as a valuable new case study for the evolution of long life.

## Results

### Reduced ageing parameters in pollen-feeding *Heliconius* species

**Fig. 1:**
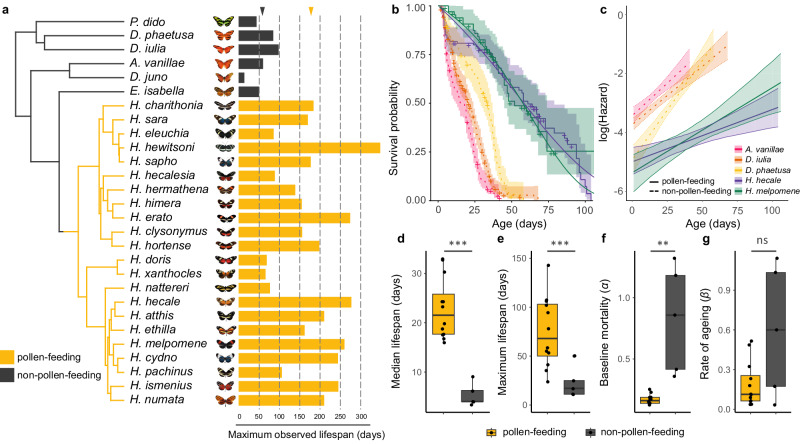
Pollen-feeding *Heliconius* spp. have longer lifespans and reduced ageing parameters relative to non-pollen feeding relatives. **a** Phylogeny of the Heliconiini tribe with associated bars representing maximum reported lifespans based on Table 1 and 3. Branches are coloured according to feeding habit. Arrowheads indicate the mean maximum lifespan for each feeding habit. **b** Kaplan-Meier survival estimates (stepped lines) and 95% confidence intervals (shaded regions) overlaid with the corresponding parametric survival curves (smooth curves) for the multi-species cognitive experiment cohort. *n*_*A. vanillae*_ = 175, *n*_*D. iulia*_ = 263, *n*_*D. phaetusa*_ = 108, *n*_*H. hecale*_ = 120, *n*_*H. melpomene*_ = 66. “+” indicates a censored data point. **c** Model-estimated log(Hazard) (equivalent to log[likelihood of death]) curves (lines) and 95% confidence intervals (shaded regions) resulting from the transformation of the parametric survival function shown in (**b**) to the hazard function. An increase in the intercept on this graph represents an increase in baseline mortality (*α*); an increase in the slope represents an increase in the rate of ageing (*β*). Boxplots represent overall differences between pollen-feeders (*n* = 12) and non-pollen-feeders (*n* = 5) from the semi-natural “mark-release-recapture” cohort in (**d**) median lifespan estimates, (**e**) maximum observed lifespan, (**f**) baseline mortality estimates, and (**g**) rate of ageing estimates. Boxplots show the median (centre line), interquartile range (box bounds), whiskers extending to the most extreme data points within 1.5 × the interquartile range, and outliers plotted individually. Indications of statistical significance for these boxplots are taken from results of two-tailed *t*-tests for median (*p* = 2.33 × 10^−5^) and maximum (*p* = 0.008) lifespans, and two-tailed Mann-Whitney U tests for baseline mortality (*p* = 3.23 × 10^−4^) and rate of ageing (*p* = 0.195), without controlling for phylogenetic relatedness; see Supplementary Note 1 for results of phylogenetic ANOVA. *H. hecale* image reproduced and cropped from^19^ under CC BY 4.0 (https://creativecommons.org/licenses/by/4.0/); all other butterfly images reproduced and cropped from^15^ under CC BY 4.0 (https://creativecommons.org/licenses/by/4.0/). Source data are provided as a Source Data file.

Data collated from published field studies and public butterfly houses revealed a 25-fold range in maximum reported lifespan for species across the Heliconiini tribe, from 14 days in *Dione juno* to an observed maximum of 348 days in *Heliconius hewitsoni* (Fig. 1a and Table 1). Pollen-feeding *Heliconius* species have longer maximum reported lifespans, with a mean of 177.36 days as compared to 57.67 days for non-pollen-feeding Heliconiini outgroups (*t*_21.88_ = 5.81, *p* < 0.001). The phylogenetic signal for median lifespan is high (Pagel’s *λ* = 0.72), such that phylogenetic regression confounds the statistical significance of the group difference (Supplementary Note 1), consistent with a major effect of the transition to pollen-feeding in *Heliconius*.

Survival data from a multi-species cohort exposed to a cognitive experiment^19^ also revealed longer lifespans in pollen-feeding *Heliconius* species (Fig. 1b and Table 2), with a mean maximum lifespan of 105.00 days for pollen-feeders as compared with 55.67 days for non-pollen-feeders (*t*_3_ = – 4.84, *p* = 0.017), and a mean median lifespan of 56.50 days for pollen-feeders as compared with 24 days for non-pollen-feeders (*t*_3_ = – 4.38, *p* = 0.022). Sex was not a significant predictor of survival in any species (*D. phaetusa*: *χ*^2^_1_ = 0.54, *p* = 0.464; *D. iulia*: *χ*^2^_1_ = 1.43, *p* = 0.231; *H. hecale*: *χ*^2^_1_ = 0.24, *p* = 0.627; *H. melpomene*: *χ*^2^_1_ = 0.02, *p* = 0.884) with the exception of *A. vanillae* (*χ*^2^_1_ = 13.38, *p* < 0.001). However, this exception appeared to be due to disproportionately high early mortality in *A. vanillae* males, as sex was no longer a significant predictor of survival when the population was subset to those that survived at least the first 5 days (*χ*^2^_1_ = 2.87, *p* = 0.090). Beyond lifespan, parametric survival analysis of the multi-species cognitive experiment cohort showed differences between species in both baseline mortality (*α*) and the rate of ageing (*β*) (see “Methods”), with the best-supported model allowing both parameters to vary by species. Both of these parameters suggested reduced ageing in pollen-feeding *Heliconius* (Fig. 1c and Table 2), though these did not always exceed formal statistical thresholds of significance (see Supplementary Note 2).

**Table 2:** Ageing parameters for the multi-species cognitive experiment cohort

Species	Feeding habit	Median lifespan (days)	Maximum lifespan (days)	Baseline mortality (${{{\boldsymbol{\alpha }}}}$)	Rate of ageing (${{{\boldsymbol{\beta }}}}$)
*Agraulis vanillae*	non-pollen-feeding	17	41	0.033	0.044
*Dryadula phaetusa*	non-pollen-feeding	34	58	0.0075	0.067
*Dryas iulia*	non-pollen-feeding	21	68	0.024	0.040
*Heliconius hecale*	pollen-feeding	61	104	0.0070	0.018
*Heliconius melpomene*	pollen-feeding	52	106	0.0050	0.029

Data from a much larger sample of species across the Heliconiini tribe provided further evidence of reduced ageing in *Heliconius*. Results from Bayesian survival trajectory analysis of the semi-natural “mark-release-recapture” cohort showed pollen-feeding *Heliconius* to have higher maximum (*t*_15_ = 3.05, *p* = 0.008) and median lifespans (*t*_15_ = 6.02, *p* < 0.001) as well as reduced baseline mortality (*α*) (*W* = 0, *p* < 0.001) compared with non-pollen-feeding outgroups (Fig. 1d–f, Table 3 and Supplementary Note 3). Rate of ageing (*β*) is generally slower in pollen-feeding *Heliconius*, but this difference is not significant (*W* = 17, *p* = 0.195) (Fig. 1g, Table 3 and Supplementary Note 3). Median lifespan in this cohort appears to be significantly underestimated compared to the more thorough survival data from the multi-species cognitive experiment cohort. However, median lifespan still showed a significant positive correlation with maximum reported lifespans from Table 1 (Supplementary Note 3; *r* = 0.62; *t*_15_ = 3.07, *p* = 0.008).

**Table 3:** Ageing parameters for the semi-natural “mark-release-recapture” cohort

Species	Feeding habit	Median lifespan (days)	Maximum lifespan (days)	Baseline mortality (${{{\boldsymbol{\alpha }}}}$)	Rate of ageing (${{{\boldsymbol{\beta }}}}$)
*Agraulis vanillae*	non-pollen-feeding	6.27	11	0.42	0.16
*Dione juno*	non-pollen-feeding	4.03	11	0.86	0.15
*Dryadula phaetusa*	non-pollen-feeding	8.93	17	0.36	0.086
*Dryas iulia*	non-pollen-feeding	3.33	25	1.32	0.025
*Eueides isabella*	non-pollen-feeding	4.03	50	1.18	0.0047
*Heliconius atthis*	pollen-feeding	16.63	53	0.25	0.016
*Heliconius charithonia*	pollen-feeding	–	22	–	–
*Heliconius cydno*	pollen-feeding	19.78	55	0.19	0.026
*Heliconius doris*	pollen-feeding	17.68	35	0.14	0.07
*Heliconius erato*	pollen-feeding	33.08	94	0.12	0.0092
*Heliconius hecale*	pollen-feeding	18.73	78	0.23	0.013
*Heliconius hewitsoni*	pollen-feeding	17.68	41	0.17	0.046
*Heliconius himera*	pollen-feeding	–	17	–	–
*Heliconius ismenius*	pollen-feeding	15.93	24	0.15	0.074
*Heliconius melpomene*	pollen-feeding	30.28	102	0.14	0.0087
*Heliconius numata*	pollen-feeding	23.28	106	0.17	0.016
*Heliconius pachinus*	pollen-feeding	24.33	58	0.13	0.033
*Heliconius sapho*	pollen-feeding	32.73	143	0.13	0.0056
*Heliconius sara*	pollen-feeding	24.33	107	0.19	0.0053
*Philaethria dido*	non-pollen-feeding	–	7	–	–

*H. charitonia*, *H. himera*, and *P. dido* were excluded from Bayesian survival trajectory analysis due to low sample size, and so only maximum lifespan was collected for these species.

### Dietary pollen does not explain full lifespan extension in *Heliconius*

**Fig. 2:**
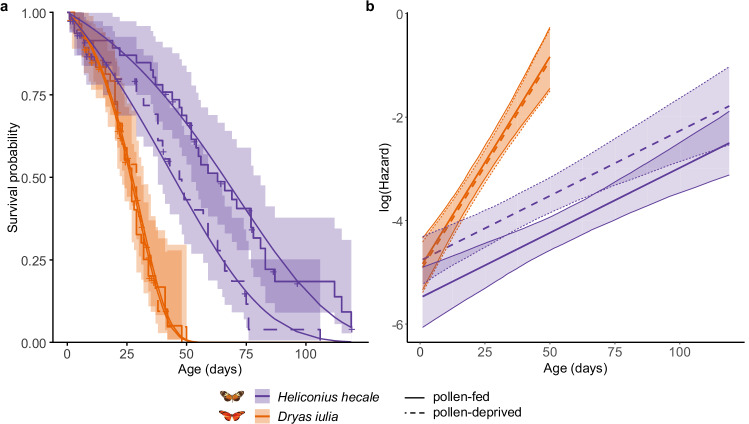
H. hecale *shows a longer lifespan and a slowed rate of ageing compared to* D. iulia. **a** Kaplan-Meier survival estimates (stepped lines) and 95% confidence intervals (shaded regions) overlaid with the corresponding parametric survival curves (smooth curves) for the pollen-manipulation experiment cohort. “+” indicates a censored data point. **b** Model-estimated log(Hazard) (equivalent to log[likelihood of death]) curves (lines) and 95% confidence intervals (shaded regions) resulting from the transformation of the parametric survival function shown in (**a**) to the hazard function. An increase in the intercept on this graph represents an increase in baseline mortality (*α*); an increase in the slope represents an increase in the rate of ageing (*β*). Survival data was collected from 96 individuals of *H. hecale* (*n*_pollen-fed_ = 47, *n*_pollen-deprived_ = 49) and 116 individuals of *D. iulia* (*n*_pollen-fed_ = 57, *n*_pollen-deprived_ = 57). *H. hecale* image reproduced and cropped from^19^ under CC BY 4.0 (https://creativecommons.org/licenses/by/4.0/); *D. iulia* image reproduced and cropped from^15^ under CC BY 4.0 (https://creativecommons.org/licenses/by/4.0/). Source data are provided as a Source Data file.

We next conducted detailed survival analyses in two focal Heliconiini species to test the effects of pollen on longevity: *H. hecale*, as a representative pollen-feeding *Heliconius*, and *D. iulia*, as a representative non-pollen-feeding outgroup. Diet (i.e., provision of pollen) was a significant predictor of survival in *H. hecale* (*χ*^2^_1_ = 8.86, *p* = 0.003), with a median survival of 47 days (maximum: 106 days) for the pollen-deprived group and a median survival of 63 days (maximum: 119 days) for the pollen-fed group (Fig. 2a and Table 4). In contrast, diet was not a significant predictor of survival in *D. iulia* (*χ*^2^_1_ = 0.24, *p* = 0.624), with a median survival of 29 days (maximum: 50 days) for the pollen-deprived group and a median survival of 27 days (maximum: 48 days) for the pollen-fed group (Fig. 2a and Table 4). This is lower than both diet groups in *H. hecale*, implying both diet-dependent and diet-independent increases in longevity in *Heliconius*. Neither sex (*H. hecale*: *χ*^2^_1_ = 0.73, *p* = 0.393; *D. iulia*: *χ*^2^_1_ = 1.58, *p* = 0.208) nor eclosion mass (*H. hecale*: *χ*^2^_1_ = 0.73, *p* = 0.530; *D. iulia*: *χ*^2^_1_ = 0.16, *p* = 0.693) were found to be significant predictors of survival in either species. Parametric survival analysis showed that this reduced lifespan in pollen-deprived *H. hecale* was due to an increase in baseline mortality (*α*), which was 1.98 times higher than that of the pollen-fed group, while the rate of ageing (*β*) remained unchanged in pollen-deprived individuals, with the best-supported model allowing *α*, but not *β*, to vary by diet (Fig. 2b and Table 4). In accordance with its lack of effect on *D. iulia* lifespan, diet also had no effect on either baseline mortality (*α*) or rate of ageing (*β*) in this species (Fig. 2b, Table 4 and see Supplementary Note 4).

**Table 4:** Ageing parameters for the pollen-manipulation experiment cohort

Species	Diet treatment	Median lifespan (days)	Maximum lifespan (days)	Baseline mortality (${{{\boldsymbol{\alpha }}}}$)	Rate of ageing (${{{\boldsymbol{\beta }}}}$)
*Heliconius hecale*	pollen-fed	63	119	0.0044	0.025
	pollen-deprived	47	106	0.0087	0.025
*Dryas iulia*	pollen-fed	27	48	0.0077	0.081
	pollen-deprived	29	50	0.0071	0.081

### Pollen deprivation accelerates body mass decline in *Heliconius*

**Fig. 3:**
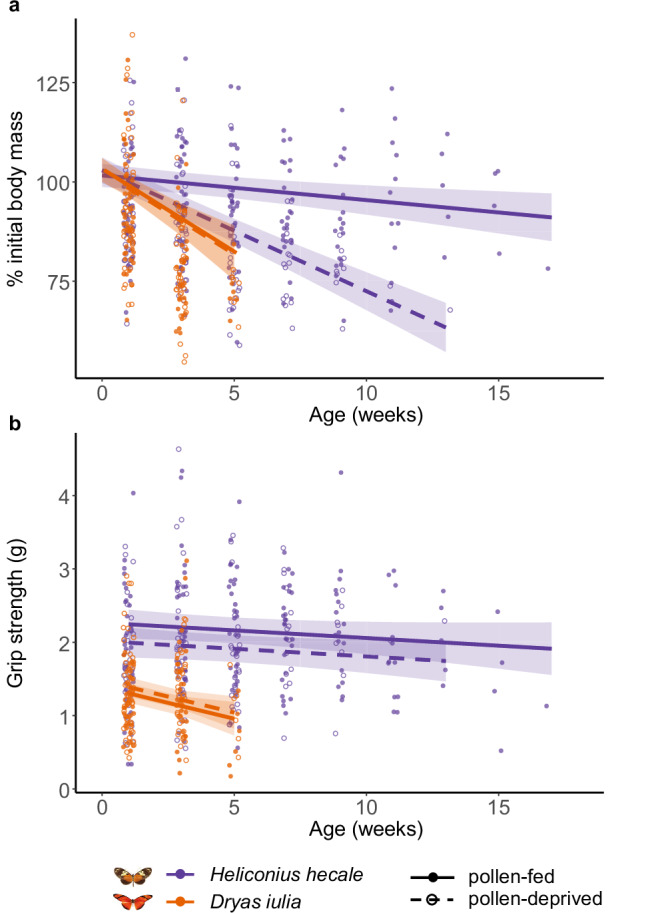
H. hecale *shows slowed functional senescence in comparison with* D. iulia*.* **a** Change in body mass with age. Both *H. hecale* and *D. iulia* show a decline in body mass with increasing age, but this is steeper in *D. iulia* and in pollen-deprived *H. hecale*. Dots represent individual data points, normalised to initial body mass to better visualise age-related change. **b** Change in grip strength with age. Grip strength declines with age in *D. iulia*, but not in *H. hecale*, although pollen-deprived *H. hecale* were weaker overall. Dots represent individual data points indicating the maximum weight a butterfly was capable of lifting with its true legs. Lines represent model-predicted mean values for each trait (centre) derived from linear mixed-effects models for each species / diet combination, with shaded areas indicating 95% confidence intervals around the predicted mean. Body mass and grip strength data was collected from 96 individuals of *H. hecale* (*n*_pollen-fed_ = 47, *n*_pollen-deprived_ = 49) and 116 individuals of *D. iulia* (*n*_pollen-fed_ = 57, *n*_pollen-deprived_ = 57). *H. hecale* image reproduced and cropped from^19^ under CC BY 4.0 (https://creativecommons.org/licenses/by/4.0/); *D. iulia* image reproduced and cropped from^15^ under CC BY 4.0 (https://creativecommons.org/licenses/by/4.0/). Source data are provided as a Source Data file.

We also measured indices of physiological senescence across the lifespan of *H. hecale* and *D. iulia* under pollen-fed and pollen-deprived diets. Both species showed an age-related decline in body mass regardless of diet treatment. However, there was a significant interaction between species, diet, and age (*F*_1, 376.62_ = 9.69, *p* = 0.002), such that pollen-deprived *H. hecale* showed a steeper body mass decline with age than the pollen-fed group, losing an estimated 3.50% of their body mass per week, compared with 1.06% for pollen-fed butterflies (Fig. 3a; *F*_1, 301.09_ = 64.88, *p* < 0.001). In contrast, and in keeping with the lack of a diet effect on longevity, in *D. iulia* there was no difference in rate of body mass decline between diet treatments; both groups lost an estimated 6.50% of their body weight per week (Fig. 3a; *χ*^2^_1_ = 0.28, *p* = 0.599), although the pollen-deprived group overall weighed an estimated 9.54% less than the pollen-fed group from the onset of the experiment (*F*_1, 128.68_ = 5.08, *p* = 0.026). Across both species, there was a significant interaction between age and sex, with body mass in males declining more steeply with age than in females (*F*_1, 376.93_ = 17.71, *p* < 0.001), an effect which persisted across the full *H. hecale* lifespan (*F*_1, 301.41_ = 4.18, *p* = 0.042). There was also a species-specific effect of longevity, whereby longer-lived *H. hecale* individuals were generally heavier (*F*_1, 97.82_ = 8.32, *p* = 0.005), whereas this was not the case for *D. iulia* (*F*_1, 137.52_ = 0.01, *p* = 0.924).

### Muscle function declines with age in *D. iulia*, but not in *H. hecale*

In addition to body mass, we measured butterfly grip strength as an index of functional senescence. Grip strength weakened with age in *D. iulia* (Fig. 3b; *F*_1, 99.37_ = 6.78, *p* = 0.011), with butterflies pulling an estimated 0.35 g less on week 5 than on than week 1, a reduction of 25.67%. However, this was not true of *H. hecale*, which did not show signs of a decline in grip strength with age, even across their much longer lifespan (Fig. 3b; *F*_1, 228.82_ = 2.62, *p* = 0.107). Diet did not have an impact on grip strength in *D. iulia* (Fig. 3b; *F*_1, 83.20_ = 1.14, *p* = 0.289), but in *H. hecale*, pollen-deprived butterflies were weaker than the pollen-fed group (Fig. 3b; *F*_1, 72.90_ = 5.05, *p* = 0.028), pulling an estimated 0.25 g less, a reduction of 12.15%. However, there was no interaction between age and diet in *H. hecale* (Fig. 3b; *χ*^2^_1_ = 2.30, *p* = 0.130), meaning that the impact of pollen-deprivation was consistent across the full lifespan. In *H. hecale*, females were stronger than males (*F*_1, 71.65_ = 4.83, *p* = 0.031), pulling an estimated 0.23 g more (12.34%), while there was no sex difference in *D. iulia* (*χ*^2^_1_ = 0.53, *p* = 0.467). Eclosion mass was also a significant predictor of grip strength across all butterflies (*F*_1, 180.82_ = 2.62, *p* < 0.001), with heavier butterflies capable of pulling heavier loads. Once again, there was a species-specific effect of longevity, whereby longer-lived *H. hecale* individuals were stronger (*F*_1, 97.01_ = 4.29, *p* = 0.041), while this was not the case for *D. iulia* (*F*_1, 114.93_ = 2.25, *p* = 0.136).

## Discussion

Research into long-lived species across the animal kingdom holds extraordinary potential for uncovering novel mechanisms of healthy ageing. In particular, the diversity of lifespan found in insects^7^, with their experimental tractability and long history of ageing research, presents a fertile ground for studying the evolution of extended life. Butterflies in the *Heliconius* genus have previously been reported to live up to 6 months^11^, suggesting a major lifespan extension over their non-pollen-feeding Heliconiini relatives^14,16^, and positioning them as a potential candidate system for such research. However, the paucity of published longevity data for many species within this tribe has previously limited the reliability of this lifespan extension. Collating maximum longevities from decades of published research on Heliconiini and utilising the presence of these charismatic butterflies in commercial butterfly houses for additional data, we reveal 25-fold variation in recorded maximum lifespan across the tribe. This range far exceeds previous estimates, and is among the largest ever recorded for such closely-related taxa (with comparable differences reported only for two groups of fish: rockfishes^26^ and roughies^27^). The maximum reported lifespan of 348 days for *Heliconius hewitsoni* also exceeds any butterfly previously recorded in the scientific literature. However, we now present the non-*Heliconius* nymphalid *Myscelia cyaniris*, with a maximum reported lifespan of 380 days, as the longest-lived butterfly species to date based on data from butterfly exhibitors (Supplementary Data 1). Our data on parameters of ageing across the Heliconiini tribe, combined with a more detailed analysis of two focal longer- and shorter-lived species, show that the transition to a novel pollen-feeding behaviour in *Heliconius* is associated with the evolution of slowed actuarial and physiological senescence and increased lifespan in this genus. Our results provide a much-needed foundation for future research into mechanisms of longevity in this new model system for the study of long life. Here we discuss three main results: i) that pollen-fed *Heliconius* outlive pollen-deprived *Heliconius*, but that ii) *Dryas iulia* gains no passive benefit of pollen; and that iii) both with and without pollen, *Heliconius* show reduced ageing over the outgroup Heliconiini, suggesting heritable mechanisms of delayed senescence.

Results from survival analysis of *Heliconius hecale* showed direct, plastic benefits of the pollen-fed diet treatment to lifespan, with a 16-day increase in median lifespan, consistent with the direction of previous results in *Heliconius charithonia*^14^. Parametric survival analysis shows that this increased lifespan in pollen-fed *H. hecale* is mediated through a reduction in baseline mortality independent of age. This may suggest that pollen-deprived individuals are more vulnerable to extrinsic hazards such as predation, starvation, or infectious diseases^25^, which aligns with some of the potential nutritional benefits of dietary pollen. For example, pollen contains a high proportion of lipids^28^, which are important in insect immunity^29^, and may directly contribute to increased lifespan through enhanced immune defences. This is supported by similar experimental work showing lifespan benefits of dietary pollen in several other pollen-feeding insects. For example, in honey bees, pollen provision extends lifespan roughly two-fold, increasing longevity by 19.5 days compared with sugar-fed controls^30^. Studies in hoverflies^31^ and wasps^32^ have also reported lifespan benefits of dietary pollen, although the increases are more modest, generally limited to just a few days.

These direct benefits of pollen consumption on physiological condition, irrespective of age, are neatly mirrored in the superior performance of pollen-fed *H. hecale* in the grip strength assay, which assesses muscle function. The consistency of this enhanced grip strength across the entire lifespan suggests that it is not due to beneficial impacts of pollen-derived amino acids on musculature, in which case we would expect to see an interaction between diet and age, whereby pollen-deprived butterflies gradually grew weaker as they got older. It may instead reflect a greater energy budget in these butterflies, both due to pollen-derived lipids, which are important energy storage compounds^33^, and due to pollen-derived amino acids. For example, the amino acid proline plays a critical role in energy storage in insect muscle and haemolymph^34^, and has been found in high proportions in the pollen of *Psiguria*, the preferred pollen source of *Heliconius* used in this study^35^. Regardless, improved physiological condition may have ecological relevance for fitness in pollen-fed *Heliconius*, conferring advantages in competition for resources, or through enhanced predator avoidance, as reflected in their reduced baseline mortality. None of these direct plastic fitness benefits of pollen was found in *D. iulia*, despite the possibility of incidental amino acid uptake given the capacity of pollen to release free amino acids in nectar^36^. This suggests that *Heliconius* have not only evolved the ability to actively collect and digest pollen, but also the physiological adaptations necessary to fully exploit its nutritional benefits – adaptations that are absent in *D. iulia* and likely in the other non-pollen-feeding Heliconiini.

Previous work has suggested that *Heliconius’* lifespan extension is an exclusively plastic response to adult pollen intake, reporting that pollen-deprivation reduces *Heliconius* lifespan to that of their non-pollen-feeding Heliconiini relatives^14^. Contrary to this, our results demonstrate a 20-day increase in median lifespan from *D. iulia* to pollen-deprived *H. hecale*, showing that this lifespan extension is evolved and persists even in the absence of pollen. Life history theory predicts that increased investment in lifespan should come with concomitant costs to other traits, such as fecundity^37^. The disposable soma theory similarly places the evolution of ageing within a resource allocation framework, arguing that it is the compromise between allocation of limited resources to either reproduction or somatic maintenance that ultimately leads to senescence^38^. However, the boundaries of this framework may not be so tightly fixed if there is a change in the reserve of available resources. Both on a plastic, individual level^39^, and on an evolutionary timescale^7,40^, enhanced nutrition can increase an organism’s overall fitness by positively impacting one life history trait without incurring a complementary cost. The evolution of pollen-feeding in *Heliconius* seems to have loosened life history constraints by adding a valuable additional dietary resource, shifting the nutritional burden of reproduction to the adult stage^14^.

Support for this shift comes from isotopic evidence for transfer of essential amino acids from pollen to eggs^20^, as well as the finding that while larval nutrition and development time are similar across pollen-feeding and non-pollen-feeding Heliconiini^41^, the proportion of nitrogenous resources allocated to reproduction at eclosion is inversely correlated to expected adult nitrogen intake^42^. This suggests that in *Heliconius*, a greater proportion of larval-derived resources may instead be allocated to somatic maintenance and longevity, accounting for the extended lifespans of even pollen-deprived *H. hecale* relative to *D. iulia*. This is supported by the results of our grip strength assay, in which both pollen-fed and pollen-deprived *H. hecale* lack senescence in physiological condition, in contrast to ageing *D. iulia*, making it unlikely that the burden of somatic maintenance is shifted to adult-acquired nitrogenous reserves. This is further reinforced by the observed patterns of body mass decline, which is thought to result from the depletion of stored reproductive reserves^17^, and is attenuated in *H. hecale* as compared to *D. iulia* in accordance with their enhanced adult diet. Without the expected supplementation of nitrogenous reserves, pollen-deprived *H. hecale* suffer a steeper decline in body mass as reproductive reserves rapidly deplete. However, this decline is still intermediate between that of *D. iulia* and the pollen-fed *H. hecale*. Coupled with the lack of physiological senescence shown in pollen-deprived *H. hecale* and their extended lifespans relative to *D. iulia*, this suggests that some larval reserves are kept aside for somatic maintenance regardless of reproductive deficits imposed by a nitrogen-poor adult diet.

Placing these findings in an evolutionary context suggests that the transition to pollen-feeding in *Heliconius*, and the prolonged reproductive lifespan it permits^14^, may have caused the “selection shadow” central to evolutionary theories of ageing to retreat to higher reaches of the lifespan. This means that increased reproductive longevity will expose older ages to selection, favouring the evolution of pro-longevity mechanisms; for example, the proposed shift in resource allocation towards somatic maintenance suggested by our results in *H. hecale*. Such a shift in life-history strategy would have been favoured by the relative constancy of *Heliconius’* preferred pollen sources throughout the year^43^, as well as the year-round availability of host-plants for oviposition (despite evidence for partial seasonal defoliation in sub-tropical environments)^44^. Considering that the pollen-related extension of reproductive lifespan has thus far only been demonstrated for females^14^, and that males in any case generally collect less pollen^43^, our lack of evidence for a sex effect on *Heliconius* survival was unexpected. However, this may simply be because of correlated selection on male longevity due to positive genetic correlations across the sexes, or perhaps because the polyandrous mating system of the two *Heliconius* species for which we analysed sex-specific survival (*H. hecale* and *H. melpomene*)^45^ is thought to lead to stronger selection on male longevity^46^.

The prolonged reproductive lifespan permitted by *Heliconius’* pollen-feeding behaviour provides a robust evolutionary explanation for their three-fold increase in maximum lifespan over the non-pollen-feeding Heliconiini outgroups (Table 1 and Fig. 1a). Beyond maximum lifespan, estimated survival trajectories for 17 Heliconiini species also show differences in median lifespan, baseline mortality (*α*), and rate of ageing (*β*) across the tribe, as well as within the *Heliconius* genus. The transition to pollen-feeding corresponds to reduced baseline mortality (*α*) and rate of ageing (*β*), and in accordance with this, lengthened lifespans in *Heliconius*. However, we acknowledge that the singular evolution of this pollen-feeding behaviour makes it difficult to formally support causative associations due to a lack of statistical power that would be provided by repeat transitions. While several other insects, including bees^30^, hoverflies^31^, wasps^32^, and moths^47^ are also known to feed on pollen, most of these also suffer from a lack of comparative lifespan data, as well as distinctly different ecologies to *Heliconius*, preventing comparative inferences about the impact of pollen feeding on longevity more generally. We also lack lifespan data from the Aoede clade, the only non-pollen-feeding *Heliconius* (previously considered a separate genus, *Neruda*), where this trait appears to be secondarily lost^15^. However, particular support for an association with pollen-feeding and/or other derived *Heliconius* traits comes from our data from the non-pollen-feeding *Eueides*, as the genus most closely related to *Heliconius*^15^. Despite this closer phylogenetic proximity, the maximum reported lifespan for *Eueides isabella* of 50 days (from our own data in Table 3) places it comfortably within the range of maximum lifespans displayed by the other non-pollen-feeding Heliconiini outgroups, and well below that of all *Heliconius*.

An alternative explanation may be that the increase in longevity is linked to the chemical defences of *Heliconius*. Indeed, there is some experimental evidence that *Heliconius* adults are less palatable to predators than those of the other Heliconiini outgroups^48^, which some suggest may have strengthened selection for lengthened lifespans due to a reduction in extrinsic mortality^16^. Despite the intuitive power of this explanation^5^, evolutionary theorists have shown that such a reduction in extrinsic mortality would only select for longer life if this reduction specifically applied to older age classes^49^. In the juvenile stage, *Heliconius* larvae are also unpalatable^50^, but there is currently no experimental evidence to suggest that they are less so than adults, nor that any putative difference between the age classes is disproportionately greater than in the outgroup Heliconiini. While our data reflect a singular evolution of pollen-feeding at the base of *Heliconius*, we believe that the overlapping habitats, host-plants, common juvenile life histories across Heliconiini, and the diet-dependent effects of pollen on longevity, leave pollen-feeding as a clear ecological difference distinguishing *Heliconius* from outgroup genera, and one which could explain the observed shift in adult lifespan with a sound basis in life history theory. However, additional studies, such as predation experiments across age classes in *Heliconius* and the Heliconiini outgroups, would help to evaluate alternative possibilities.

Regardless of the evolutionary basis of this lifespan extension, our phylogenetically-broad survival analysis showed that increased longevity is widespread across the *Heliconius* genus. This provided the required background to narrow our focus to our two representative longer- and shorter-lived species, *H. hecale* and *D. iulia*, to better understand the underlying basis of this longevity. While lifespan data are informative, modelling age-specific mortality via parametric survival analysis can provide additional information on patterns of ageing, allowing for deeper insights into the biological underpinnings of such lifespan extensions^24^. An “extended lifespan” does not necessarily mean that senescence has been delayed, but may instead reflect a reduction in baseline mortality that results in greater survival irrespective of age, as has been shown for example, across mammalian females^51^. The results of the pollen-manipulation experiment show that while *H. hecale* and *D. iulia* show similar baseline mortality, *H. hecale* has evolved a slower rate of ageing, resulting in the observed lifespan extension. This slowed rate is maintained even under pollen-deprivation, suggesting that this parameter is unrelated to the short-term plastic effects of adult diet (Fig. 2b). Our results are of particular significance considering *D. iulia*’s position as one of the longest-lived non-pollen-feeding outgroups (Tables 1–3), as we find clear differences in metrics of ageing in our experiments despite the apparent capacity for lengthy lifespans in some individuals of the species. While the wide variation in both of these parameters across the tribe makes it unlikely that this pattern is precisely generalisable to all Heliconiini, our combined results across several cohorts consistently suggest a slowed rate of ageing as a major contributor to lengthened lifespan in *Heliconius*.

A reduction in the rate of ageing implies the presence of mechanisms that delay or slow physiological senescence^25^. This is illuminated by results from the grip strength assay, which show an age-related decline in grip strength in *D. iulia* and provide for a reliable index of physiological senescence in Heliconiini butterflies. This assay has been proposed to measure whole-organism performance in butterflies^52^, citing similar metrics that predict fitness in beetles^53^, and hand grip strength is also used as a biomarker of health in ageing humans^54^. In *D. iulia*, declining grip strength is likely an indicator of deteriorating muscular health, with age-related declines in muscular structure and function also reported in several other insects^55^. This decline in *D. iulia* provides a possible physiological basis for their faster actuarial senescence as compared to *H. hecale*, and this assay links actuarial and physiological senescence in this system, with a lower overall grip strength also associated with higher baseline mortality in pollen-deprived *H. hecale*. In contrast with *D. iulia*, ageing *H. hecale* showed no evidence of a comparable decline in grip strength, maintaining functional performance even at very advanced ages. That this deterioration is mitigated in *H. hecale* well beyond the limits of *D. iulia*’s lifespan suggests that, in accordance with their lifespan extension, *Heliconius* have also evolved a delay in physiological senescence, presenting a valuable opportunity for dissecting the mechanisms underlying healthy ageing.

Our data provide a rare longitudinal perspective on functional senescence in a long-lived butterfly genus, and present evidence for an evolved lifespan extension, slowed actuarial senescence, and delayed physiological senescence in *H. hecale*, independent of any short-term, plastic benefits of adult pollen intake. The applicability of these findings to other *Heliconius* species may depend on ecological factors such as the degree of pollen reliance^43^, and would be illuminated by further studies of senescence in other *Heliconius* species. Indeed, the wide variation in lifespan between *Heliconius* species, with maximum lifespans for some even falling within the range of the outgroup Heliconiini genera (Fig. 1a and Table 1), offers many testable hypotheses for putative pro-longevity mechanisms in this genus, possibly related to species-specific differences in ecology. It is possible that some of this variation may reflect differences in response to caged environments; however, a major strength of the work presented here is our combination of several very different sources of data on Heliconiini ageing, spanning records from semi-natural butterfly house exhibits and mark-release recapture experiments in the wild in addition to those from caged insectary populations. We believe that our triangulation of these complementary datasets helps to strengthen our overall conclusions, and makes our inferences robust to species-specific responses to any one experimental set-up. Our collation of multiple records per species across many studies (Tables 1–4 and Supplementary Data 2) also highlights the limitations of using maximum lifespan as the sole metric of ageing, as large intra-species differences suggest that these values are often driven by single long-lived individuals. Despite this, our data span multiple metrics of ageing and combine several independent sources to show that any intra-*Heliconius* variation is surpassed by the broader differences between *Heliconius* and the other Heliconiini (Fig. 1), making our in-depth findings in *H. hecale* a productive representation of this genus. Consistent evidence across the Heliconiini tribe points to conserved pro-longevity mechanisms in *Heliconius*, presenting a promising target for investigation and establishing *Heliconius* as a powerful new model for the study of extended longevity. In particular, the wealth of genomic tools available in this genus makes it a highly tractable system for mechanistic investigations. Future work exploring the proximate basis of *Heliconius*’ extended “healthspan” would provide a deeper understanding of the tools used by these butterflies to unpick the constraints imposed by ageing.

## Methods

### Heliconiini maximum reported lifespan data collation

Data on maximum reported lifespan for species across the Heliconiini tribe (Table 1 and Supplementary Data 2) were collated from a literature search within PubMed, Google Scholar, and Web of Science for long-term studies of *Heliconius* and other Heliconiini butterflies, using combinations of the search terms “*Heliconius*”, “Heliconiini”, “lifespan”, “longevity”, “survival”, “mark”, “release”, “recapture”, and “population dynamics”, first between January and April 2021, and then again in November 2025. However, considering the popularity of Heliconiini species in commercial butterfly houses, active exhibits listed on the International Association of Butterfly Exhibitors and Suppliers were also contacted to enquire if they had collected lifespan data. For many species, multiple records of maximum longevity were found, sourced from data from butterfly exhibitors, mark-release-recapture studies, and insectary populations (Supplementary Data 2). In all such instances, maximum longevity records from butterfly exhibitors eclipsed those from insectary populations and mark-release recapture studies in the wild. One study in particular (Kelson, unpublished) was responsible for many of the highest maximum longevity records; for further details of this study design, see Supplementary Note 5.

### Butterfly husbandry and survival data collection

All wild-caught Panamanian butterflies were collected under permit numbers SE/A-82-19 and SE/A-14-18.

### Survival data from a multi-species cognitive experiment cohort

Survival data were collected for *Heliconius hecale melicerta, Heliconius melpomene rosina*, *Dryadula phaetusa*, and *Agraulis vanillae* during a previous cognitive experiment by Young et al.^19^. Survival data for *Dryas iulia* were obtained during a similar cognitive experiment^56^ generated under the same experimental conditions. In both studies, all butterflies were captive-reared from stock populations at the Smithsonian Tropical Research Institute (STRI) outdoor insectaries in Gamboa, Panama. These populations were established from wild-caught individuals collected within a 2 km radius of Gamboa, between January-May 2019 (for *H. melpomene*, *H. hecale*, *D. phaetusa*, and *A. vanillae*), or between January-November 2022 (for *D. iulia*). Eggs were collected daily from host-plants within these stock cages, and hatched larvae were reared in mesh pop-up cages and fed *ad libitum* on leaves of their preferred *Passiflora* host-plants. *H. melpomene* was fed on *P. triloba*, *H. hecale* was fed on *P. vitifolia*, and *D. phaetusa*, *A. vanillae*, and *D. iulia* were fed on *P. biflora*.

Upon eclosion, butterflies were marked with a unique ID using a contrastingly-coloured marker and placed into 2 m (L) x 3 m (W) x 2 m (H) cages containing a rack of 24 artificial feeders arranged in a 4 × 6 grid and placed centrally in the cage. Feeders contained approximately 0.5 ml of a sucrose-protein solution (25% w/v sucrose and 5% w/v Vetark Critical Care Formula), replaced daily. A single non-flowering *Palicourea elata* plant was also placed in each cage as a roosting site. Adult butterflies were subjected to long-term memory assays based on the association of a colour with a food reward, which imposed regular bouts of food deprivation during testing periods as well as frequent handling. Further details of these assays may be found in Young et al.^19^. Upon completing the cognitive experiment, butterflies were maintained until the end of their natural lifespans. Cages were checked daily for dead individuals, death dates were recorded, and missing or predated individuals were noted for censorship in survival analysis at the age at which they were last seen alive. In total, survival data from both sexes were analysed for 175 individuals of *A. vanillae*, 263 individuals of *D. iulia*, 108 individuals of *D. phaetusa*, 120 individuals of *H. hecale*, and 66 individuals of *H. melpomene*.

### Semi-natural “mark-release-recapture” cohort

To obtain survival data across a broader sampling of Heliconiini, butterflies of 20 different species were released into a large semi-natural enclosure for a mark-release-recapture study. The cohort comprised all Heliconiini species available at the STRI insectaries during the study period, maximising phylogenetic coverage. All individuals were captive-reared from stock populations between January and November 2022 on leaves of their preferred *Passiflora* host-plants. Provenance of these stock populations varied depending on the species, with many established from local Panamanian collecting trips, and others established using pupae imported from butterfly farms in the years preceding data collection. Newly-eclosed butterflies were marked with a unique ID using a contrastingly-coloured marker and released into a large 11 m (L) x 11 m (W) x 6 m (H) cage located at the STRI insectaries. The cage was filled with trees, *Passiflora* host-plants, and other flowering plants, creating a semi-natural environment. Floral nectar sources were also supplemented by artificial feeders containing a 20% w/v sucrose solution, replaced every 2 days.

In total, 964 butterflies of 20 different Heliconiini species were released into the cage, including: *A. vanillae* (*n* = 12), *D. iulia* (*n* = 34), *Dione juno* (*n* = 45), *D. phaetusa* (*n* = 29), *Eueides isabella* (*n* = 59), *Heliconius atthis* (*n* = 33), *Heliconius charithonia* (*n* = 3), *Heliconius cydno* (*n* = 18), *Heliconius doris* (*n* = 32), *Heliconius erato* (*n* = 44), *H. hecale* (*n* = 47), *Heliconius hewitsoni* (*n *= 15), *Heliconius himera* (*n* = 2), *Heliconius ismenius* (*n* = 19), *H. melpomene* (*n* = 276), *Heliconius numata* (*n* = 92), *Heliconius pachinus* (*n* = 19), *Heliconius sapho* (*n* = 81), *Heliconius sara* (*n* = 71), and *Philaethria dido* (*n* = 13). All *Heliconius* included in this study are pollen-feeding species, and data for the four non-pollen feeding *Heliconius* in the Aoede clade are unfortunately not available. This reflects the understudied nature of these species, which occur at low densities and have a derived host plant and are therefore challenging to work with. The sample number reflects the availability of pupae. Both sexes were represented for all species.

Data collection was carried out twice per week, with some omissions due to time constraints, and usually by a single surveyor. Approximately 15 min were spent patrolling the cage and recording the IDs of any butterflies visually identified and alive on that date. To ensure minimal intervention, butterflies were not recaptured or handled. Cage cheques were typically conducted mid-morning, when Heliconiini butterflies are most active, but an effort was made to also check at other times to re-sight individuals where this differed. Of the 959 butterflies released into the cage, 448 individuals were re-sighted on at least one occasion. Due to the size of the cage and semi-natural conditions, the largest source of extrinsic mortality in the cages was predation due to spiders or ants. In the case of natural deaths, individuals were rarely recovered before being eaten by ants, or otherwise deteriorated. In the event that a dead individual’s body was recovered intact, it was assumed to have died that day, and so its death date was noted.

### Pollen-manipulation experiment cohort

All butterflies used in the longitudinal pollen-manipulation experiments were reared from stock populations at the STRI outdoor insectaries in Gamboa, Panama, between January and November 2022. Stock populations of *H. hecale* and *D. iulia*, selected for their local abundance and ease of rearing, were established from wild-caught individuals collected within a 2 km radius of Gamboa. New individuals were collected and added to stock populations bi-weekly to ensure genetic diversity, with stock populations of each species in total comprising approximately 60 females and 40 males across the 7-month study period. Stocks were maintained in 2 m (L) x 2 m (W) x 2 m (H) cages containing artificial feeders filled with a 20% w/v sucrose and 10% w/v organic, pesticide-free bee pollen solution, changed every 2 days. All stock cages contained flowering *Palicourea, Lantana*, and *Stachytarpheta* plants, and *H. hecale* stocks were also provided with fresh *Psiguria* flowers daily. Eggs were collected daily from host plants within these stock cages. Hatched larvae were reared in pop-up mesh cages and fed *ad libitum* on shoots of one of their preferred *Passiflora* host-plants, depending on availability: *P. biflora*, *P. auriculata, P. pittieri*, or *P. edulis* for *D. iulia*; and *P. nitida, P. riparia*, or *P. vitifolia* for *H. hecale*.

Upon eclosion, butterflies were sexed and weighed, their forewings were measured, and they were marked with a unique ID using a contrastingly coloured marker. They were then randomly assigned to either a pollen-fed or pollen-deprived treatment, with otherwise matched conditions and resources. The pollen-fed cage contained approximately 12 flowering *Palicourea*, *Lantana*, and *Stachytarpheta* plants, as well as fresh *Psiguria* flowers, replaced daily. It also contained 4 central artificial feeders filled with a 20% w/v sucrose and 10% w/v organic bee pollen solution, changed daily. The pollen-deprived cage contained approximately 12 non-flowering *Palicourea*, *Lantana*, and *Stachytarpheta* plants, as well as 4 central artificial feeders and approximately 20 red star-shaped feeders hung around the plant foliage, all filled with a 20% w/v sucrose solution, also changed daily. The number of artificial feeders in this cage was increased to match the number of flowers + feeders in the pollen-fed cage to ensure feeding opportunities were as standardised as possible between cages. Each cage included both species in roughly equal proportions, preventing confounding of species with treatment. Individuals were added to both cages in a staggered manner over the 7-month study period. Both cages measured approximately 4 m (L) x 3 m (W) x 2 m (H), and contained *P. biflora* and *P. vitifolia* host-plants.

Cages were checked daily for dead individuals, death dates were recorded, and missing or predated individuals were noted for censorship in survival analysis at the age at which they were last seen alive. In total, survival data from both sexes were collected across the lifespan of 96 individuals of *H. hecale* (*n*_pollen-fed_ = 47, *n*_pollen-deprived_ = 49) and 116 individuals of *D. iulia* (*n*_pollen-fed_ = 57, *n*_pollen-deprived_ = 57).

### Functional senescence assays

Butterflies from the pollen-manipulation experiment cohort (*n*_H. hecale_ = 96; *n*_D. iulia_ = 116) were measured every two weeks for indices of functional senescence (see Supplementary Fig. S1 for a schematic). Considering evidence from several insects for age-related declines in body mass^57^, and muscle function^55^, butterflies were first weighed using a Sartorius Entris balance and then assayed for grip strength using a method adapted from Davis et al.^52^. Grip strength provides a proxy for whole organism condition in butterflies^52^ and beetles^53^, and is a standard biomarker of health in humans^54^. A custom-built device consisting of a perch mounted on a lightweight base (see Supplementary Fig. S2 for a diagram) was placed on the balance, which was then tared. Butterflies were held by their wings and allowed to grasp the perch, then gently pulled upwards until they released it. The peak negative reading on the balance from this exercise was taken as a measure of grip strength, which we took to be a proxy for muscle function. Following the methodological approach of Davis et al.^52^, this was repeated five times for each individual, and the maximum reading was used for statistical analysis, as we were primarily interested in each individual’s maximum capacity. However, this metric was also found to correlate extremely highly with the mean reading for each individual; see Supplementary Note 6 for further details. Data on flight behaviour were also recorded and are presented in Supplementary Note 7; see Supplementary Methods for details of how these assays were performed.

### Statistical analyses

All statistical analyses were conducted using R v4.3.1^58^. For all individuals, “age” was measured beginning from the time of adult eclosion, and so does not reflect the larval and pupal stages. These generally last about 3 weeks in Heliconiini butterflies, with only minor differences (1-2 days) between species^41^. All survival analyses presented in this manuscript are therefore conducted using only adult lifespan, coinciding with the earliest age at reproduction, as is traditionally suggested when modelling senescence^5^.

### Non-parametric and semi-parametric survival analyses

Non-parametric and semi-parametric survival analyses for the pollen-manipulation experiment and multi-species cognitive experiment cohorts were conducted with the aid of the packages *survival* v3.5-5^59^ and *coxme* v2.2-18^60^. Cox proportional hazards models were created for each species, including adult eclosion mass, diet, and sex (pollen-manipulation experiment cohort) or just sex (multi-species cognitive experiment cohort) as fixed effects. Interspecific models were found during diagnostics to have violated the proportional hazards assumption, and so instead separate models for each species were created within each cohort.

### Parametric survival analyses

The package *flexsurv* v2.2.2^61^ was then used to create parametric survival models for these cohorts fit to a Gompertz distribution (see Supplementary Note 8 for an explanation of distribution selection). The Gompertz function models how mortality risk changes with age, and is described by the equation *µ*(*x*) *= αe*^*βx*^, where *µ*(*x*) is the instantaneous mortality rate at age *x*, *α* is the baseline mortality independent of age, and *β* is the age-dependent mortality rate (i.e., the relative change in mortality with age *x*), also known as the rate of ageing^24^ (but see Supplementary Note 9 for an alternative index of the rate of ageing). A value of *β* > 0 reflects an increase in mortality risk with age, confirming the presence of actuarial senescence, and reflecting the process of ageing. Graphically speaking, when age is plotted against the natural log (ln) of the mortality rate, the intercept is equal to ln(*α*) and the slope is equal to *β*, facilitating easy interpretation of these parameters: a higher intercept reflects an increase in baseline mortality risk, and a higher slope reflects an increased rate of ageing. The corresponding Gompertz survival function is given as $S(x)={e}^{\left(\right.\frac{\alpha }{\beta }(1-{e}^{\beta {{{\mathcal{x}}}}})}$, where *S*(*x*) is the probability of surviving to age *x*; this transformation facilitates the conversion in Figs. 1, 2 from the parametric survival curves overlaying the empirical survival data (Figs. 1b, 2a) to their corresponding log(Hazard) curves (Figs. 1c, 2b).

As the initial Cox proportional hazards models showed no evidence for effects of sex or eclosion mass on survival in the pollen-manipulation experiment and multi-species cognitive experiment cohorts, neither of these predictors were included in further parametric analyses. Instead, parametric models were fit, allowing either *α* – baseline mortality, *β* – rate of ageing, both, or neither to vary based on species (multi-species cognitive experiment cohort) or species, diet, and their interaction (pollen-manipulation cohort). The parametric model with the lowest AIC was then used to assess whether species or diet predicted either of these parameters. Bootstrapped estimates (1000 iterations) were generated for *α* and *β* for each group of interest, and the mean and 95% confidence intervals were extracted from these distributions. Estimates for these parameters were considered to be significantly different between groups if their 95% confidence intervals did not overlap. The *H. melpomene* dataset from the multi-species cognitive experiment cohort was subset to just those individuals surviving over 1 week due to high early mortality in this species (see Supplementary Note 10).

### Bayesian survival trajectory analysis

Longevity data from the semi-natural “mark-release-recapture” cohort were analysed using the R package *BaSTA* v1.9.5^62^, which facilitates the use of incomplete recapture data to provide estimates of age-specific survival under a Bayesian framework. *BaSTA* uses estimates of recapture probability based on the frequency of recaptures for each population to model survival trajectories. Considering that recapture probabilities likely differed between species due to factors such as behavioural differences, and given that census lengths differed for different species depending on when they were first introduced to the study, we created separate models in *BaSTA* for each species. Models were fit to a simple Gompertz distribution, running four parallel *BaSTA* simulations with 11,000 iterations, a burn-in of 1001, and a thinning rate of 200 to minimise serial autocorrelation. Median lifespan for each species was taken from estimates of survival probability in the *BaSTA* survQuant object. This produces estimates of survival probability for specified ages, increasing stepwise by 0.1 week each time, and so rarely included an age at which survival probability was exactly 50% (the median). Therefore, median survival was taken as the mean of the two ages for which predicted survival traversed 50%. Correlation between median lifespan estimates from *BaSTA* and existing reported maximum lifespans (Table 1) was assessed using Pearson’s correlation test. *BaSTA* also generates estimates for life expectancy as well as the Gompertz parameters b0 (equivalent to ln(*α*), or ln[baseline mortality]) and b1 (equivalent to *β*, or rate of ageing). These were also recorded for each species, with b0 back-transformed to *α* for ease of comparison with other cohorts. The value for maximum longevity for each species was taken as the highest age at which an individual of that species was observed alive (Table 3).

Due to the opportunistic nature of this study, data for many species suffered from low sample sizes and low recapture probabilities, which lead to wider credible intervals in *BaSTA* estimates and an underprediction of lifespan, respectively^62^. Given this, and the more thorough survival data from the other cohorts, the estimates for median lifespan for species in this cohort are almost certainly underestimates. However, these biases should apply uniformly to all species in the study. The broad patterns fit with expectations from the literature (Table 1), and we believe are still worthy of interpretation. There were several weeks over the course of this 9-month study in which cage cheques were not possible, and the reduced recapture effort for these weeks was accounted for in the models with the use of the recaptTrans argument within the basta() function. *Heliconius himera*, *Philaethria dido*, and *Heliconius charithonia* were excluded from *BaSTA*s due to low sample size and few re-sightings, and so maximum longevity was the only metric retained for these species. *BaSTA* also does not allow for recaptures during the “birth week”, and so 66 sightings which occurred in the first week of an individual’s life were excluded from analysis. Accounting for these exclusions, *BaSTA*s were performed using data from a total of 941 butterflies, with 959 recorded sightings of 378 different individuals. A full breakdown of these exclusions per species can be found in Supplementary Note 3.

### Feeding habit differences

The maximum reported lifespans from Table 1 as well as the final parameters generated from the multi-species cognitive experiment cohort and the semi-natural “mark-release-recapture” cohort, were used to assess broad differences in ageing parameters between pollen-feeders (*Heliconius* species) versus non-pollen-feeders (the outgroup Heliconiini). The Shapiro-Wilk test of normality showed all parameters derived from the multi-species cognitive experiment cohort to be normally distributed (median lifespan: *W* = 0.92, *p* = 0.530; maximum lifespan: *W* = 0.89, *p* = 0.382; Gompertz parameter *α*: *W* = 0.83, *p* = 0.129; Gompertz parameter *β*: *W* = 0.97, *p* = 0.862), and the F-test of equality of variances showed all measures to have equal variances between the two groups (median lifespan: *F*_2,1_ = 1.95, *p* = 0.903; maximum lifespan: *F*_2,1_ = 93.17, *p* = 0.146; *α*: *F*_2,1_ = 86.95, *p* = 0.151; *β*: *F*_2,1_ = 3.75, *p* = 0.686). Therefore, these parameters were assessed using a two-tailed Student’s *t* test to test for differences between feeding habits.

The Shapiro-Wilk test of normality showed median and maximum lifespans derived from the semi-natural “mark-release-recapture” cohort to be normally distributed (median: *W* = 0.94, *p* = 0.335; maximum: *W* = 0.93, *p* = 0.214), and the F-test of equality of variances showed both measures to have equal variances between the two groups (median: *F*_11,4_ = 5.24, *p* = 0.124; maximum: *F*_11,4_ = 4.90, *p* = 0.214), and so these were assessed using a two-tailed Student’s *t* test to test for differences between feeding habits. The Shapiro-Wilk test showed the Gompertz parameters *α* and *β* derived from results in this cohort to be non-normally distributed (*α*: *W* = 0.59, *p* < 0.001; *β*: *W* = 0.73, *p* < 0.001), so these were assessed using a two-tailed Mann-Whitney U test to test for differences between feeding habits.

The Shapiro-Wilk test showed maximum lifespan values from Table 1 to be normally distributed (*W* = 0.96, *p* = 0.298), and the F-test of equality of variances this measure to have unequal variances between the two groups (*F*_20,5_ = 6.72, *p* = 0.044), and so these were assessed using a two-tailed Welch’s *t* test to test for differences between feeding habits.

To control for phylogenetic relatedness, these comparisons were then repeated using the phylANOVA() function from the R package *phytools* v2.3-0^63^, run with 10,000 simulations using a trimmed phylogenetic tree taken from ref. ^15^. This package and tree were also used to create the phylogenetic tree in Fig. 1a, and to estimate phylogenetic signal as measured by Pagel’s *λ*. However, as this evolutionary transition only occurred once, we expect our results to be confounded by phylogeny, with limited power to disentangle these effects. As such, results from these phylogenetic ANOVAs are presented in Supplementary Note 1.

### Functional senescence analyses

For analysis of body mass and grip strength data from the pollen-manipulation experiment cohort, linear mixed-effects models were fit using the package *lme4* v1.1-34^64^ for two single-species datasets containing data for each species across their full lifespans (up to week 5 for *D. iulia* and up to week 17 for *H. hecale*), as well as for an interspecific dataset containing data for *H. hecale* and *D. iulia* up to week 5 (the oldest age at which there was data for *D. iulia*). All models included individual ID as a random effect to account for the non-independence of repeated measures within the same individual. All single-species “full” models included diet, age, sex, and their three-way interaction as candidate predictors. All interspecific “full” models included species, diet, age, and their three-way interaction as candidate predictors, as well as two-way interactions between species and any candidate predictors found to be significant in the single-species models. Additional candidate predictors included assay time (for body mass and grip strength), expressed as the fraction of the day elapsed since midnight, and eclosion mass (for grip strength), measured in grams. We were primarily interested in within-individual differences related to senescence, and so individual longevity was also included as a fixed effect to control for among-individual differences, ensuring unbiased estimates of the effect of age and thus accounting for the possibility of selective disappearance^65^. However, models without longevity as a fixed effect did not result in qualitatively different findings, and a brief summary of these results may be found in Supplementary Note 11.

For all models, stepwise backward model selection was implemented by performing an ANOVA on the model with and without a named predictor, to see if inclusion of the predictor significantly improved model fit. The final “best” model was then compared with an intercept-only “null” model to confirm its validity, also using an ANOVA. Age, diet, and species (for the inter-specific models) were always included in the final model as these were the effects of interest, as was longevity, which ensured unbiased age estimates. An ANOVA was then run on the final model to report the significance of named predictors. If an effect was not included in the final model, the nonsignificant result of the ANOVA from model selection resulting in its removal was instead reported.

### Reporting summary

Further information on research design is available in the Nature Portfolio Reporting Summary linked to this article.

## Supplementary information

Supplementary Information

Description of Additional Supplementary Files

Supplementary Data 1-9

Reporting Summary

Transparent Peer Review file

## Source data

Source Data

## Data Availability

All data generated in this study have been deposited in an associated figshare repository and may be accessed here: 10.6084/m9.figshare.31081597. This includes all lifespan data from Mr. Kelson’s butterfly house census, maximum lifespan records from the literature and other commercial butterfly houses, as well as lifespan and trait data for all other butterflies in this study. Source data are provided in this paper.

## Code availability

All R scripts used for analysis have been deposited in an associated figshare repository and may be accessed here: 10.6084/m9.figshare.31081597.
