# Supplementary material for: Evolution of increased longevity and slowed ageing in a genus of tropical butterfly
Source: Nat Commun. 2026 Jun 16;17:5077. doi: 10.1038/s41467-026-73635-7 (PMC13272878; doi:10.1038/s41467-026-73635-7)
Supplement: Supplementary file 1 — Supplementary Information [file 41467_2026_73635_MOESM1_ESM.pdf]

## Supplementary Material for:

Evolution of increased longevity and slowed ageing in a genus of tropical butterfly

Jessica Foley, Josie McPherson, Made Roger, Cruz Batista, Rémi Mauxion, Greta Hernández, Richard Kelson, Fletcher J. Young, W. Owen McMillan, Stephen H. Montgomery

## Table of Contents

1. Supplementary Methods ........ 2
   1.1 Additional details on functional senescence assays ........ 2
2. Supplementary Notes ........ 4
   2.1 Supplementary Note 1: Results of comparisons between feeding habits when controlling for phylogenetic relatedness ........ 4
   2.2 Supplementary Note 2: Details of parametric survival analysis for the multi-species cognitive experiment cohort ........ 5
   2.3 Supplementary Note 3: Details of survival analysis for the semi-natural "mark-release-recapture" cohort ........ 8
   2.4 Supplementary Note 4: Details of parametric survival analysis for the pollen-manipulation experiment cohort ........ 10
   2.5 Supplementary Note 5: Maximum reported lifespans from butterfly exhibitors ........ 12
   2.6 Supplementary Note 6: Correlation between maximum grip strength reading and mean across trials ........ 14
   2.7 Supplementary Note 7: Additional results on flight behaviour in ageing *H. hecale* and *D. iulia* ........ 15
   2.8 Supplementary Note 8: Hazard function distribution selection ........ 17
   2.9 Supplementary Note 9: Comparison of indices of actuarial senescence ........ 22
   2.10 Supplementary Note 10: *H. melpomene* early deaths exclusion ........ 24
   2.11 Supplementary Note 11: Functional senescence results without longevity included as a covariate ........ 26

References ........ 28

# 1. Supplementary Methods

### *1.1 Additional details on functional senescence assays*

Butterflies from the pollen-manipulation experiment cohort ($n_{H.\ hecale}$ = 96; $n_{D.\ iulia}$ = 116) were assayed with a battery of tests every two weeks, beginning one week after eclosion, and continuing until their natural death (Fig. S1). During testing, individuals were removed from their cages at approximately 10:00, and subjected first to a flight behaviour assay, as several insects show age-related declines in flight capacity[1-3], followed by the body mass and grip strength assays. The flight behaviour assay was performed in the outdoor insectaries, and the body mass and grip strength assays were performed in the neighbouring laboratory. Testing generally concluded by 13:00, after which butterflies were returned to their respective cages.

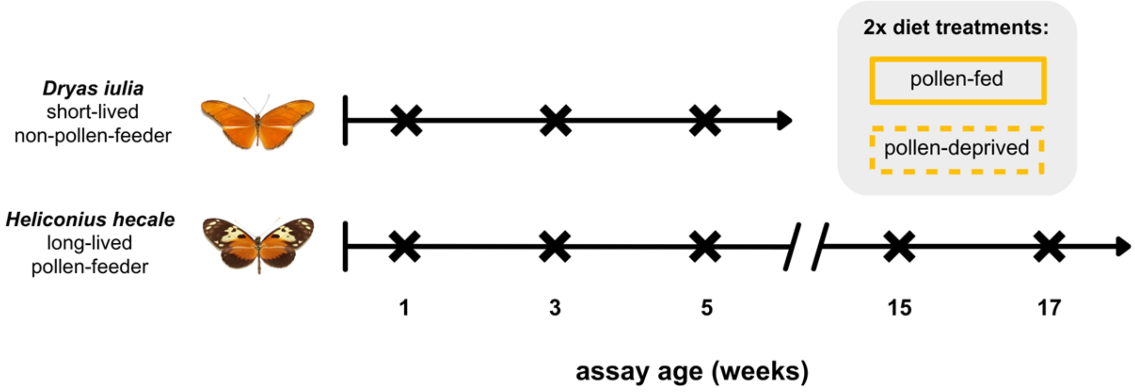


**Fig. S1: Experimental design.** Individuals of both *H. hecale* and *D. iulia* were maintained under either a pollen-fed or pollen-deprived treatment and assayed on a biweekly basis until death, beginning at day 7 post-eclosion and continuing every 2 weeks thereafter. Butterfly images reproduced and cropped from[25] under CC BY 4.0 (https://creativecommons.org/licenses/by/4.0/).

Grip strength was assayed using a custom-built device named *The Pullinator*, which consisted of a lightweight wooden base upon which was mounted a small wooden perch of diameter 9.5mm, wrapped in 400 grit sandpaper to standardise friction (Fig. S2).

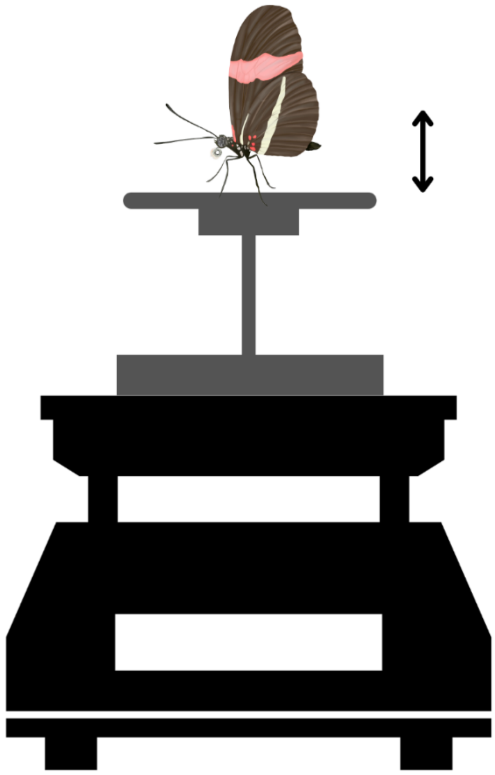

**Fig. S2:** ***The Pullinator*** **grip strength assay apparatus.** Figure created using Canva; butterfly illustration by Amaia Alcalde Antón, used with permission.

The flight behaviour assay was conducted by releasing each individual from a height of 1.5m at the end of a narrow flight arena measuring 13m (L) x 0.75m (W) x 2m (H). Individuals were monitored for a 45-second interval, and total time spent in active flight was recorded. All butterflies were assayed twice, with an interval of at least 5 minutes between each assay. Measurements taken from the assay with the maximum time spent in active flight were then used for statistical analysis. Light intensity (illuminance) within the flight arena was recorded before each assay using a digital light meter to account for any impact of weather on flight behaviour. Daily weather variables including the ultraviolet index (UVI) and whether it was raining were also noted at the time of experiments. Results from this assay are presented in Supplementary Note 7.

For analysis of flight behaviour data, generalised linear mixed models were fit with an *ordbeta* distribution[4], based on recommendations for the analysis of continuous proportion data[5], using the R package *glmmTMB* v1.1.7[6]. Additional candidate predictors included forewing length (measured in millimetres [mm]), illuminance (measured in lux), assay time, whether it was raining, and whether the butterfly had an intact wing apex (used as an index of wing-wear). Values for illuminance were centred and scaled to aid model convergence.

## 2. Supplementary Notes

### *2.1 Supplementary Note 1: Results of comparisons between feeding habits when controlling for phylogenetic relatedness*

In order to control for phylogenetic relatedness, all comparisons of ageing parameters between feeding habit (pollen-feeding versus non-pollen-feeding) were then tested using a phylogenetic ANOVA. This rendered the difference between feeding habits in maximum reported lifespans (Table 1) nonsignificant ($F$ = 13.55, $p$ = 0.210), while the difference between feeding habits in median ($F$ = 19.16, $p$ = 0.078) and maximum ($F$ = 23.39, $p$ = 0.064) lifespans from the smaller multi-species cognitive experiment cohort also showed nonsignificant trends. When applied to the larger semi-natural “mark-release-recapture” cohort, the difference between feeding habits in maximum lifespan was no longer significant ($F$ = 9.31, $p$ = 0.240). We conclude that because this evolutionary transition to pollen-feeding only occurred once at the base of a monophyletic clade, there is insufficient information to disentangle effects of phylogeny and pollen-feeding. It is notable, however, that differences between feeding habits in median lifespan estimates ($F$ = 36.28, $p$ = 0.03) and baseline mortality ($\alpha$) ($F$ = 29.57, $p$ = 0.048) in the semi-natural “mark-release-recapture” cohort remained significant even when controlling for phylogenetic relatedness, while the trends in other analyses are consistent with our wider interpretations. As such, while we cannot discount phylogenetic effects as a factor in our analyses, the pronounced shifts we observed are unlikely to have evolved under neutral evolution.

## *2.2 Supplementary Note 2: Details of parametric survival analysis for the multi-species cognitive experiment cohort*

The best-supported parametric survival model for the multi-species cognitive experiment cohort allowed both baseline mortality ($\alpha$) and the rate of ageing ($\beta$) to vary by species (Table S1). Both parameters showed trends towards reduced ageing in pollen-feeding *Heliconius*, with baseline mortality ($\alpha$) highest in *A. vanillae* (non-pollen-feeding), followed by *D. iulia* (non-pollen-feeding), *D. phaetusa* (non-pollen-feeding), *H. hecale* (pollen-feeding), and finally *H. melpomene* (pollen-feeding); however, statistical differences did not partition with respect to feeding habit, with baseline mortality ($\alpha$) for *A. vanillae* and *D. iulia* significantly higher to that of *D. phaetusa*, *H. hecale*, and *H. melpomene* (see Table S2 for details of overlapping confidence intervals and Table S3 for full contrasts). Similarly, rate of ageing ($\beta$) was highest in *D. phaetusa* (non-pollen-feeding)¸ followed by *A. vanillae* (non-pollen-feeding), *D. iulia* (non-pollen-feeding), *H. melpomene* (pollen-feeding), and finally *H. hecale* (pollen-feeding); however, the only statistically significant differences were between *D. phaetusa* versus *D. iulia*, *H. hecale*, and *H. melpomene*; as well as between *H. hecale* versus both *A. vanillae* and *D. iulia* (Tables S2 and S4). When parameters were pooled for pollen-feeders ($n$ = 2) and non-pollen-feeders ($n$ = 3), neither baseline mortality ($\alpha$) ($t_3$ = 1.60, $p$ = 0.208) nor the rate of ageing ($\beta$) ($t_3$ = 2.30, $p$ = 0.105) segregated statistically by feeding habit, likely related to the lack of power in this comparison. Despite this, both median and maximum lifespan segregated by feeding habit in statistical analysis (see Results).

**Table S1:** Comparison with Akaike information criterion (AIC) of parametric survival models fit to data from each species in the multi-species cognitive experiment cohort using a Gompertz distribution. Different models allowed either the rate parameter, the shape parameter, both, or neither, to vary by species. The rate parameter is the Gompertz parameter $\alpha$, or baseline mortality, and the shape parameter is the Gompertz parameter $\beta$, or rate of ageing. $n_{A.\ vanillae}$ = 175, $n_{D.\ iulia}$ = 263, $n_{D.\ phaetusa}$ = 108, $n_{H.\ hecale}$ = 120, $n_{H.\ melpomene}$ = 66.

| Model specification | ΔAIC | Degrees of freedom |
|---|---|---|
| rate~Species, shape~Species | 0 | 10 |
| rate~Species, shape~1 | 24.12 | 6 |
| rate~1, shape~Species | 68.63 | 6 |
| rate~1, shape~1 | 344.39 | 2 |

**Table S2:** Bootstrapped parameter estimates and confidence intervals for each species in the multispecies cognitive experiment cohort, based on the final model: rate~Species, shape~Species. Results here are presented to 2 significant figures rather than 2 decimal places due to the small size of several estimates. $n_{A.\ vanillae}$ = 175, $n_{D.\ iulia}$ = 263, $n_{D.\ phaetusa}$ = 108, $n_{H.\ hecale}$ = 120, $n_{H.\ melpomene}$ = 66. CI = confidence interval.

| Species | Feeding habit | Parameter | Parameter estimate | Lower 95% CI | Upper 95% CI |
|---|---|---|---|---|---|
| *A. vanillae* | non-pollen-feeding | *α* – baseline mortality | 0.032888 | 0.024787 | 0.043253 |
| | | *β* – rate of ageing | 0.043864 | 0.027995 | 0.058818 |
| *D. phaetusa* | non-pollen-feeding | *α* – baseline mortality | 0.00746 | 0.004405 | 0.011608 |
| | | *β* – rate of ageing | 0.067497 | 0.05166 | 0.083497 |
| *D. iulia* | non-pollen-feeding | *α* – baseline mortality | 0.023793 | 0.018824 | 0.029448 |
| | | *β* – rate of ageing | 0.039938 | 0.030494 | 0.049916 |
| *H. hecale* | pollen-feeding | *α* – baseline mortality | 0.006959 | 0.004133 | 0.010645 |
| | | *β* – rate of ageing | 0.017819 | 0.008539 | 0.027959 |
| *H. melpomene* | pollen-feeding | *α* – baseline mortality | 0.005005 | 0.002372 | 0.009322 |
| | | *β* – rate of ageing | 0.028706 | 0.014067 | 0.042447 |

**Table S3:** Inter-species contrasts for estimates of the *α* / rate parameter (baseline mortality) in the final inter-specific model for the multi-species cognitive experiment cohort: rate~Species, shape~Species. An asterisk denotes significant differences between pairs of species, based on non-overlapping confidence intervals as shown in Table S2. $n_{A.\ vanillae}$ = 175, $n_{D.\ iulia}$ = 263, $n_{D.\ phaetusa}$ = 108, $n_{H.\ hecale}$ = 120, $n_{H.\ melpomene}$ = 66. PF = pollen-feeding, NPF = non-pollen-feeding.

| | *A. vanillae* (NPF) | *D. phaetusa* (NPF) | *D. iulia* (NPF) | *H. hecale* (PF) | *H. melpomene* (PF) |
|---|---|---|---|---|---|
| *A. vanillae* (NPF) | | * | | * | * |
| *D. phaetusa* (NPF) | | | * | | |
| *D. iulia* (NPF) | | | | * | * |
| *H. hecale* (PF) | | | | | |
| *H. melpomene* (PF) | | | | | |

**Table S4:** Inter-species contrasts for estimates of the *β* / shape parameter (rate of ageing) in the final inter-specific model for the multi-species cognitive experiment cohort: rate~Species, shape~Species. An asterisk denotes significant differences between pairs of species, based on non-overlapping confidence intervals as shown in Table S2. $n_{A.\ vanillae}$ = 175, $n_{D.\ iulia}$ = 263, $n_{D.\ phaetusa}$ = 108, $n_{H.\ hecale}$ = 120, $n_{H.\ melpomene}$ = 66. PF = pollen-feeding, NPF = non-pollen-feeding.

| | *A. vanillae* (NPF) | *D. phaetusa* (NPF) | *D. iulia* (NPF) | *H. hecale* (PF) | *H. melpomene* (PF) |
|---|---|---|---|---|---|
| *A. vanillae* (NPF) | | | | * | |
| *D. phaetusa* (NPF) | | | * | * | * |
| *D. iulia* (NPF) | | | | * | |
| *H. hecale* (PF) | | | | | |
| *H. melpomene* (PF) | | | | | |

## *2.3 Supplementary Note 3: Details of survival analysis for the semi-natural "mark-release-recapture" cohort*

Results from Bayesian survival trajectory analysis on the semi-natural "mark-release-recapture" cohort showed lower baseline mortality ($\alpha$) in pollen-feeding species ($W = 0$, $p < 0.001$), but not rate of ageing ($\beta$) ($W = 17$, $p = 0.195$), although this trended lower in non-pollen-feeders. These results are presented graphically here to aid interpretation (Fig. S3B). Estimates for median lifespan from this cohort are also presented graphically here (Fig. S3A) and appear to be underestimated when compared with survival data from the other, more closely-tracked cohorts (Tables 2 and 4). However, these showed a strong positive correlation with maximum reported lifespan for each species as presented in Table 1 ($r = 0.62$; $t_{15} = 3.07$, $p = 0.008$). The correlation plot is also presented here to aid interpretation (Fig. S3C). Full details of sample size and parameter estimates for this cohort are presented in Table S5.

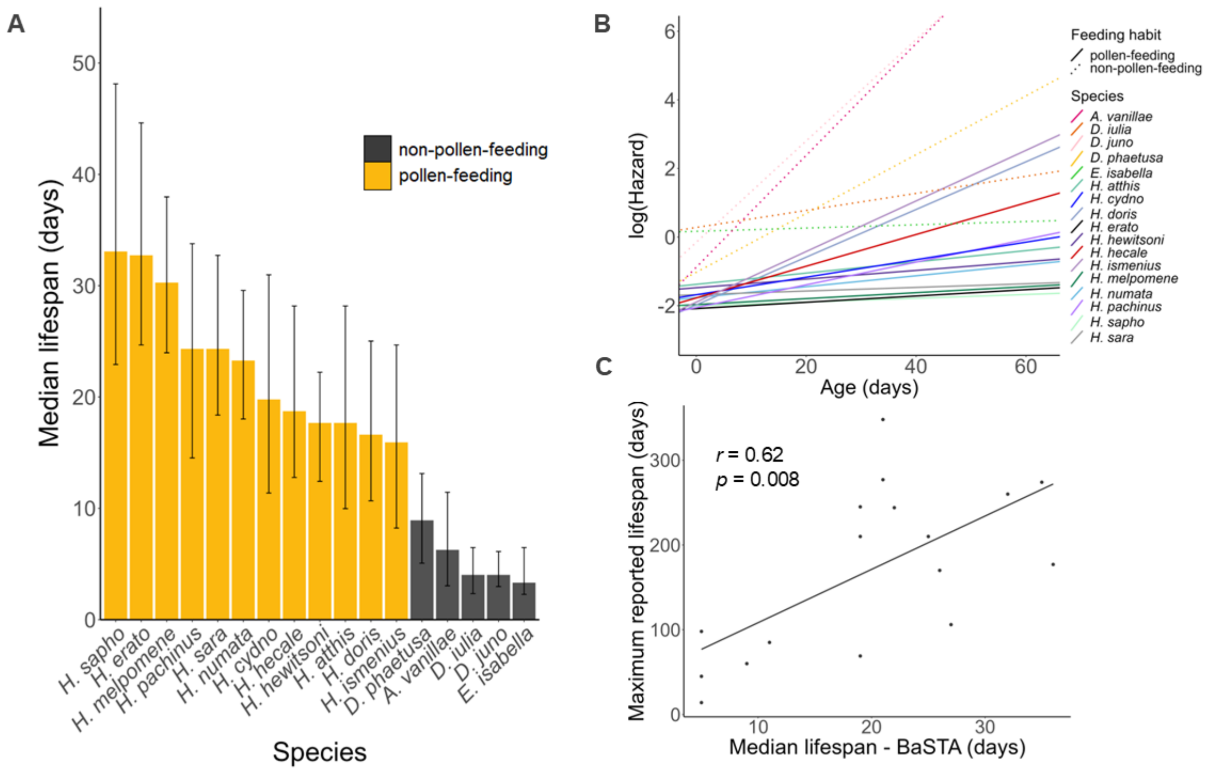


**Fig. S3: Results of *BaSTA* analysis in the semi-natural "mark-release-recapture" cohort and their relationship to existing lifespan data. A** Median lifespan estimates with 95% predictive intervals for each species in the cohort, coloured according to feeding habit. **B** log(Hazard) curves fit to a Gompertz distribution for all species in the semi-natural "mark-release-recapture" cohort, based on estimates for $\alpha$ and $\beta$ derived from *BaSTA* analysis (see Table S5 for full details). An increase in the intercept on this graph represents an increase in baseline mortality ($\alpha$); an increase in the slope represents an increase in the rate of ageing ($\beta$). **C** Correlation between median lifespan estimates derived from *BaSTA* analysis for species in this cohort and maximum reported lifespans for each species as listed in Table 1. $r$ = Pearson's correlation coefficient. $n$ numbers for each species in this cohort may be found in the Methods and Table S5. Source data are provided as a Source Data file.

**Table S5: Sample size, recorded sightings, and longevity metrics from display cage *BaSTA* results.** Species are presented in order of descending *n* sighted (original). *N* placed refers to the number of individuals marked and placed into the display cage for the study. *n* sighted (original) refers to the number of individuals that were re-sighted at least once, and *n* sighted (retained) refers to the number of these retained after excluding those sighted within the first week of life (see Supplementary Methods for details). Total detections refers to the total number of sightings for that species (many individuals were re-sighted more than once). Non-pollen-feeders are coloured in red for emphasis (see Table 3). *H. himera*, *P. dido*, and *H. charithonia* were excluded from the analysis due to low sample size. Results for $\beta$ are presented to 2 significant figures rather than 2 decimal places due to the small size of several estimates. CI = credible interval, PI = predictive interval.

| Species | *N* placed | *n* sighted (original) | *n* sighted (retained) | Total detections | $\alpha$ | | | $\beta$ | | | Median lifespan (days) | | | Maximum recorded lifespan (days) |
|---|---|---|---|---|---|---|---|---|---|---|---|---|---|---|
| | | | | | estimate | lower 95% CI | upper 95% CI | estimate | lower 95% CI | upper 95% CI | estimate | lower 95% PI | upper 95% PI | |
| *H. melpomene* | 276 | 116 | 90 | 261 | 0.14 | 0.10 | 0.19 | 0.0087 | 0.0018 | 0.016 | 30.28 | 23.98 | 37.98 | 102 |
| *H. numata* | 92 | 65 | 63 | 110 | 0.17 | 0.12 | 0.25 | 0.016 | 0.0053 | 0.025 | 23.28 | 18.03 | 29.58 | 106 |
| *H. sapho* | 81 | 50 | 40 | 183 | 0.13 | 0.09 | 0.19 | 0.0056 | 0.00038 | 0.012 | 32.73 | 24.68 | 44.63 | 143 |
| *H. sara* | 71 | 49 | 45 | 134 | 0.19 | 0.13 | 0.26 | 0.0053 | 0.00045 | 0.013 | 24.33 | 18.38 | 32.73 | 107 |
| *H. erato* | 44 | 25 | 21 | 43 | 0.12 | 0.06 | 0.21 | 0.0092 | 0.00061 | 0.023 | 33.08 | 22.93 | 48.13 | 94 |
| *H. doris* | 32 | 24 | 22 | 51 | 0.14 | 0.05 | 0.31 | 0.070 | 0.022 | 0.12 | 17.68 | 12.43 | 22.23 | 35 |
| *H. hecale* | 47 | 21 | 18 | 33 | 0.23 | 0.12 | 0.37 | 0.013 | 0.00077 | 0.032 | 18.73 | 12.78 | 28.18 | 78 |
| *H. atthis* | 33 | 16 | 15 | 34 | 0.25 | 0.11 | 0.45 | 0.016 | 0.00069 | 0.043 | 16.63 | 10.68 | 25.03 | 53 |
| *H. pachinus* | 19 | 14 | 14 | 31 | 0.13 | 0.05 | 0.31 | 0.033 | 0.0065 | 0.066 | 24.33 | 14.53 | 33.78 | 58 |
| *H. cydno* | 18 | 13 | 9 | 23 | 0.19 | 0.06 | 0.41 | 0.026 | 0.0021 | 0.060 | 19.78 | 11.38 | 30.98 | 55 |
| *D. phaetusa* | 29 | 12 | 10 | 14 | 0.36 | 0.10 | 0.91 | 0.086 | 0.010 | 0.19 | 8.93 | 5.08 | 13.13 | 17 |
| *D. juno* | 45 | 9 | 5 | 5 | 0.86 | 0.22 | 1.91 | 0.15 | 0.0097 | 0.38 | 4.03 | 2.35 | 6.48 | 11 |
| *H. hewitsoni* | 15 | 9 | 9 | 13 | 0.17 | 0.03 | 0.46 | 0.046 | 0.0051 | 0.12 | 17.68 | 9.98 | 28.18 | 41 |
| *E. isabella* | 59 | 8 | 4 | 4 | 1.18 | 0.79 | 1.74 | 0.0047 | 0.00010 | 0.016 | 4.03 | 2.98 | 6.13 | 50 |
| *H. ismenius* | 19 | 8 | 8 | 14 | 0.15 | 0.01 | 0.54 | 0.074 | 0.0054 | 0.19 | 15.93 | 8.23 | 24.68 | 24 |
| *A. vanillae* | 12 | 3 | 3 | 3 | 0.42 | 0.07 | 1.51 | 0.16 | 0.012 | 0.36 | 6.27 | 3.05 | 11.45 | 11 |
| *D. iulia* | 34 | 2 | 2 | 3 | 1.32 | 0.63 | 2.29 | 0.025 | 0.00032 | 0.086 | 3.33 | 2.28 | 6.48 | 25 |
| *H. himera* | 2 | 2 | | | | | | | | | | | | 17 |
| *P. dido* | 13 | 1 | | | | | | | | | | | | 7 |
| *H. charithonia* | 3 | 1 | | | | | | | | | | | | 22 |

### *2.4 Supplementary Note 4: Details of parametric survival analysis for the pollen-manipulation experiment cohort*

The best-supported inter-specific parametric survival model for the pollen-manipulation experiment cohort allowed the rate of ageing ($\beta$) to vary by species, and baseline mortality ($\alpha$) to vary by a two-way species:diet interaction (Table S6). The meaning of this interaction is illuminated by the two single-species models, where the best supported model in *H. hecale*, but not *D. iulia*, to vary by diet (Table S7). Table S8 shows bootstrapped parameter estimates derived from the inter-specific model for each species/diet combination.

**Table S6:** Comparison with Akaike information criterion (AIC) of parametric survival models fit to data from both species in the pollen-manipulation experiment cohort using a Gompertz distribution. Different models allowed either the rate parameter, the shape parameter, both, or neither, to vary by species, diet, and their two-way interaction. The rate parameter is the Gompertz parameter *α*, or baseline mortality, and the shape parameter is the Gompertz parameter *β*, or rate of ageing. In total, data was analysed for 96 individuals of *H. hecale* ($n_{\text{pollen-fed}}$ = 47, $n_{\text{pollen-deprived}}$ = 49) and 116 individuals of *D. iulia* ($n_{\text{pollen-fed}}$ = 57, $n_{\text{pollen-deprived}}$ = 57).

| Model specification | ΔAIC | Degrees of freedom |
|---|---|---|
| rate~Species*Diet, shape~Species | 0 | 6 |
| rate~Species*Diet, shape~Species+Diet | 1.52 | 7 |
| rate~Species*Diet, shape~Species*Diet | 3.50 | 8 |
| rate~Species*Diet, shape~1 | 25.14 | 5 |
| rate~Species*Diet, shape~Diet | 26.26 | 6 |
| rate~Species+Diet, shape~1 | 31.26 | 4 |
| rate~Species, shape~1 | 33.15 | 3 |
| rate~Diet, shape~1 | 83.54 | 3 |
| rate~1, shape~1 | 86.52 | 2 |

**Table S7:** Comparison with Akaike information criterion (AIC) of parametric survival models fit to data from both species in the pollen-manipulation cohort using a Gompertz distribution. Different models allowed either the rate parameter, the shape parameter, both, or neither, to vary by diet. The rate parameter is the Gompertz parameter *α*, or baseline mortality, and the shape parameter is the Gompertz parameter *β*, or rate of ageing. In total, data was analysed for 96 individuals of *H. hecale* ($n_{\text{pollen-fed}}$ = 47, $n_{\text{pollen-deprived}}$ = 49) and 116 individuals of *D. iulia* ($n_{\text{pollen-fed}}$ = 57, $n_{\text{pollen-deprived}}$ = 57).

| Species | Model specification | ΔAIC | Degrees of freedom |
|---|---|---|---|
| *H. hecale* | rate~Diet, shape~1 | 0 | 3 |
| | rate~1, shape~Diet | 0.32 | 3 |
| | rate~Diet, shape~Diet | 1.52 | 4 |
| | rate~1, shape~1 | 6.10 | 2 |
| *D. iulia* | rate~1, shape~1 | 0 | 2 |
| | rate~Diet, shape~1 | 1.90 | 3 |
| | rate~1, shape~Diet | 1.95 | 3 |
| | rate~Diet, shape~Diet | 3.88 | 4 |

**Table S8:** Bootstrapped parameter estimates and confidence intervals for each species/diet combination in the pollen-manipulation experiment cohort, based on the final model: rate~Species*Diet, shape~Species. Results here are presented to 2 significant figures rather than 2 decimal places due to the small size of several estimates. In total, data was analysed for 96 individuals of *H. hecale* ($n_{\text{pollen-fed}}$ = 47, $n_{\text{pollen-deprived}}$ = 49) and 116 individuals of *D. iulia* ($n_{\text{pollen-fed}}$ = 57, $n_{\text{pollen-deprived}}$ = 57). CI = confidence interval.

| Species | Diet | Parameter | Parameter estimate | Lower 95% CI | Upper 95% CI |
|---|---|---|---|---|---|
| *H. hecale* | pollen-fed | $\alpha$ | 0.0044 | 0.0023 | 0.0076 |
| | | $\beta$ | 0.025 | 0.016 | 0.034 |
| | pollen-deprived | $\alpha$ | 0.0087 | 0.0052 | 0.014 |
| | | $\beta$ | 0.025 | 0.016 | 0.034 |
| *D. iulia* | pollen-fed | $\alpha$ | 0.0077 | 0.0042 | 0.012 |
| | | $\beta$ | 0.081 | 0.063 | 0.10 |
| | pollen-deprived | $\alpha$ | 0.0072 | 0.0039 | 0.012 |
| | | $\beta$ | 0.081 | 0.063 | 0.10 |

### *2.5 Supplementary Note 5: Maximum reported lifespans from butterfly exhibitors*

For many species, multiple records of maximum longevity were found, sourced from data from butterfly exhibitors, mark-release-recapture studies, and insectary populations (Table 1; Supplementary Data 2). In all such instances, maximum lifespan records from butterfly exhibitors eclipsed those from insectary populations and mark-release recapture studies in the wild. One dataset in particular (Kelson, unpublished) was responsible for many of the highest maximum lifespan records for various species. This was provided by Mr. Richard Kelson, the entomologist running the Butterfly Habitat at Six Flags Discovery Kingdom in Vallejo, California, which was first opened in 1988 and has been running continuously under Mr. Kelson's supervision ever since. Mr. Kelson has been compiling a dataset of maximum lifespan for over 180 butterfly species kept at the exhibit since 1989, and last published on these findings in 2008[7]; the maximum lifespans presented in this paper are based on an additional 17 years of data collection.

The dimensions of the Butterfly Habitat are 30m (L) x 15m (W) x 6m (H), and floral nectar/pollen sources include (among others) *Pentas lanceolata*, *Lantana montevidensis*, *Hamelia patens*, *Stachytarpheta jamaicensis*, and *Russelia equisetiformis*. Floral nectar is supplemented with artificial feeders containing a 20% v/v honey solution, as well as bananas, papayas, and mangoes, although these are rarely visited by Heliconiini butterflies. Predation in this exhibit comes primarily from spiders, ants, and the human visitors. Humidity is 80% or higher, and temperatures range between 16-27°C from December-February, and 18-32°C from March-November. The exhibit does not keep any host-plants, meaning that females do not have the opportunity to lay (although there is occasional oviposition on non-host substrates). All pupae are sourced externally, with Heliconiini pupae typically obtained from butterfly farms in Central and South America.

The methodology for this data collection involves a) ordering pupae in batches, and recording when the last individual of each species ecloses; b) conducting a weekly species census to know which species are still alive, being especially careful to note sightings for species with only a few remaining living individuals; and c) calculating maximum longevity by subtracting the latest possible adult eclosion date from the last date an individual of that species was seen alive (so this dataset is right-censored). As an example, the *Heliconius hewitsoni* individual with a maximum recorded lifespan of 348 days came from a delivery of pupae that had all eclosed by August 30$^{th}$, 2010. The last individual seen alive from this batch was spotted during the weekly census on August 13$^{th}$, 2011.

This dataset includes many non-Heliconiini butterfly species, and an up-to-date version presented in full in Supplementary Data 1 for other researchers interested in butterfly lifespan. Species are listed alphabetically according to names used by the U.S. Department of Agriculture; where current taxonomic names are different, these are reported in parentheses. References to Watts, 2004[8] are made where that study found greater lifespans.

### *2.6 Supplementary Note 6: Correlation between maximum grip strength reading and mean across trials*

We chose to analyse maximum grip strength for each individual as a proxy for whole organism condition, as has been done previously[9-11], as we were interested in the effects of age and diet on maximal performance in these butterflies. However, we are aware that the maximum reading may be sensitive to outliers. In order to assess these concerns, we used Pearson's correlation test to measure correlation between maximum and mean grip strength across all 5 trials for each biweekly assay. We found the two measures to be highly correlated, both for *H. hecale* ($r = 0.92$, $p < 0.001$) and for *D. iulia* ($r = 0.92$, $p < 0.001$; Figure S4). We also updated the models to remove any observations with studentised residuals > 3, which is a common technique for identifying potential outliers. These models returned the same main findings, with reduced grip strength in pollen-deprived *H. hecale* ($F_{1, 73.52} = 4.60$, $p = 0.035$), but no difference between diets in *D. iulia* ($F_{1, 81.76} = 3.24$, $p = 0.076$), and a decline in grip strength with age in *D. iulia* ($F_{1, 99.48} = 11.92$, $p = 0.001$) but not *H. hecale* ($F_{1, 225.11} = 1.65$, $p = 0.201$).

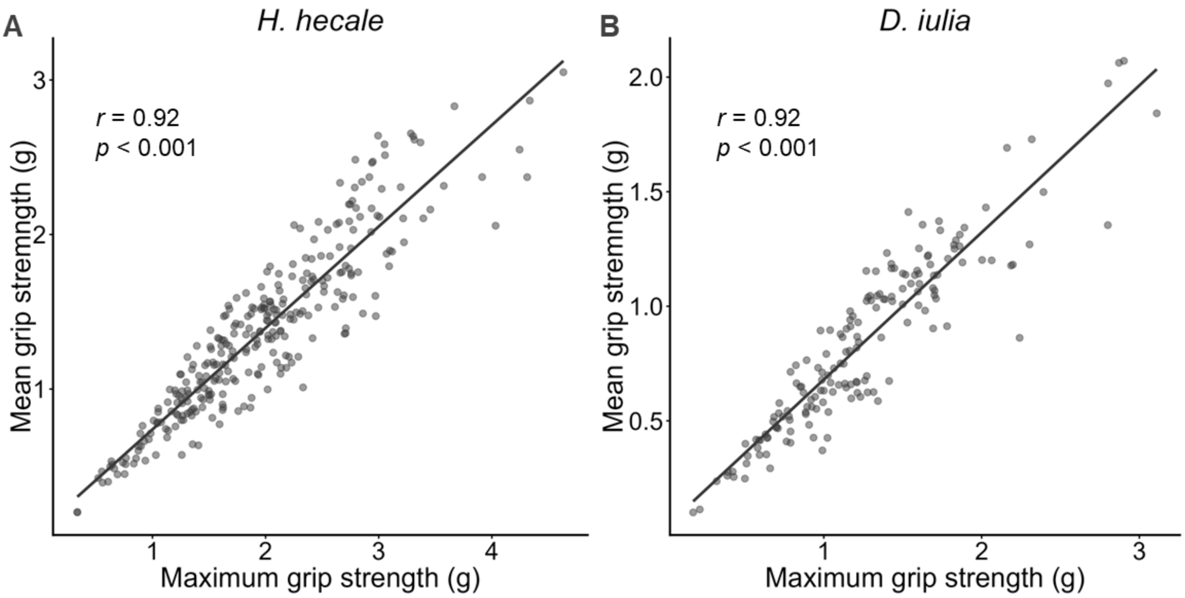


**Fig. S4: Correlation between maximum and mean grip strength.** Dots represent readings from individuals for each biweekly grip strength assay, showing the correlation between the maximum grip strength reading and the mean grip strength across all 5 trials for **A** *H. hecale* ($N = 295$) and **B** *D. iulia* ($N = 167$). Source data are provided as a Source Data file.

## *2.7 Supplementary Note 7: Additional results on flight behaviour in ageing* **H. hecale** ***and*** **D. iulia**

While *H. hecale* spent less time flying later in the day ($\chi^2_1$ = 3.95, $p$ = 0.047), in contrast to the grip strength assay, neither age ($\chi^2_1$ = 2.50, $p$ = 0.114) nor diet ($\chi^2_1$ = 0.11, $p$ = 0.735) was found to be a significant predictor of time spent in active flight in this species (Fig. S5A). In *D. iulia*, there was an interaction between age, sex, and diet ($\chi^2_1$ = 4.89, $p$ = 0.027) such that in females, pollen-fed butterflies spent more time in active flight as they aged, whereas pollen-deprived butterflies spent less time in active flight as they aged (Fig. S5B); an unexpected result perhaps indicative of a sex-specific response to the presence of natural floral cues, which would need further investigation to interrogate fully. Environment also had an impact on flight behaviour in *D. iulia*, with butterflies spending less time flying if it was raining ($\chi^2_1$ = 5.18, $p$ = 0.023). Broadly, these results suggest a lack of senescence in either butterfly in the flight activity assay, which is surprising considering the evidence for reduced flight capacity with age in other insects[2], including *Drosophila*[1] and the butterfly *Pieris napi*[3]. This is likely because in the short (45-second) assay presented here, differences in flight activity seem to reflect behavioural choices rather than physiological capacity, given that individuals flew less later in the day (*H. hecale*), or when it was raining (*D. iulia*) – behaviour which aligns with common knowledge about when these butterflies are most active[12]. Considering that many individuals were spotted flying vigorously around the cages just hours before being found dead (personal observation), it seems unlikely that a reduction in total flight capacity in ageing individuals would be observed within the 45-second assay. These results do not preclude the possibility that observed reductions in flight at the metabolic level in other butterflies[13, 14] might be also recapitulated in Heliconiini. Further investigation would help to determine if similar patterns of differential senescence between *Heliconius* and the shorter-lived Heliconiini may be identified in other aspects of their physiology.

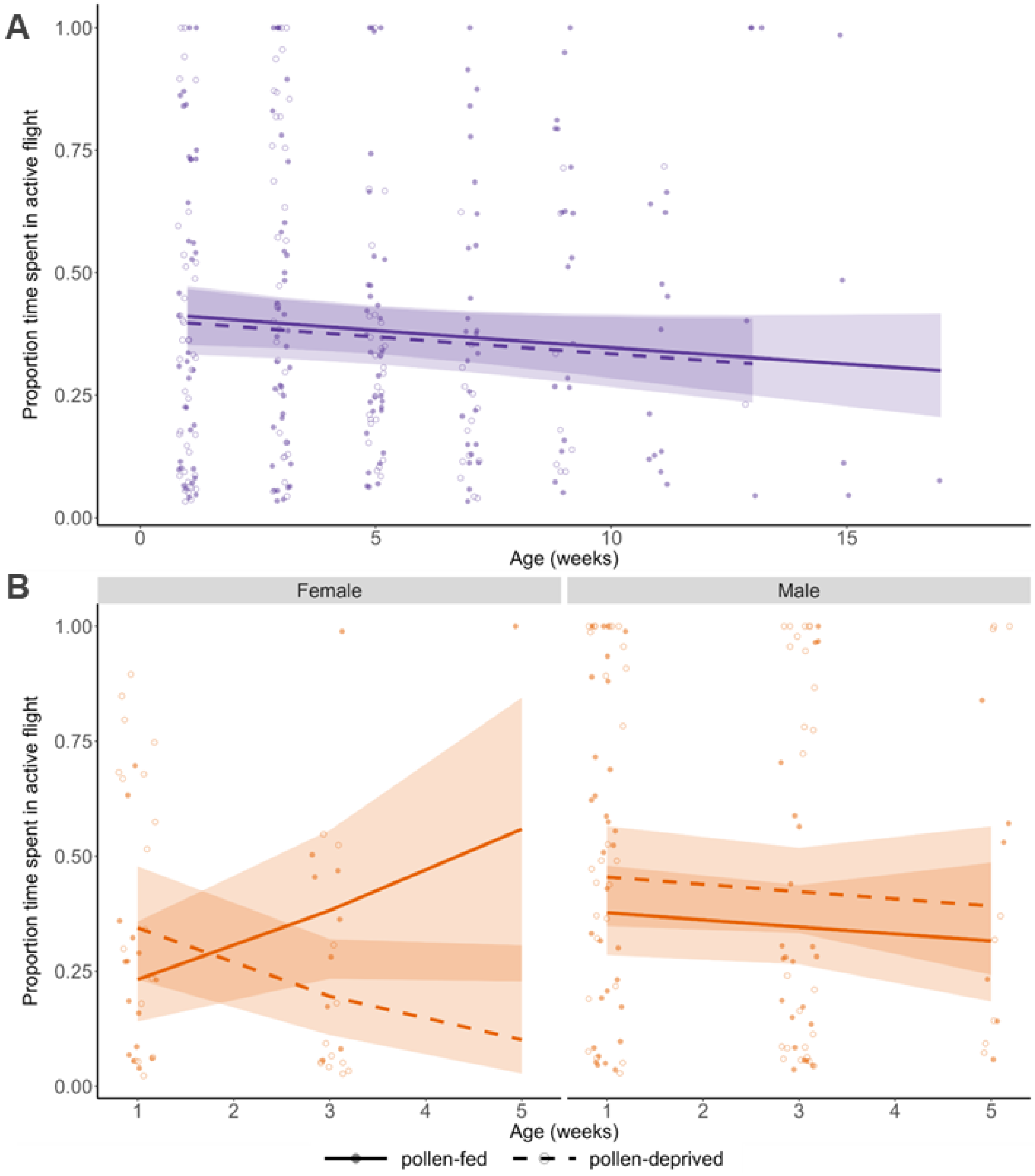


**Fig. S5: Flight behaviour results.** Dots represent individual data points indicating the proportion of time an individual spent in active flight during a 45-second assay period, for both for **A** *H. hecale* and **B** *D. iulia.* Lines represent model-predicted mean values for proportion of time spent in active flight (centre) derived from generalised linear mixed-effects models for each species / diet combination, with the sexes separated into facets for *D. iulia* due to the presence of a three-way age:sex:diet interaction. Shaded areas indicate 95% confidence intervals around the predicted mean. In total, data was analysed for 96 individuals of *H. hecale* ($n_{\text{pollen-fed}}$ = 47, $n_{\text{pollen-deprived}}$ = 49) and 116 individuals of *D. iulia* ($n_{\text{pollen-fed}}$ = 57, $n_{\text{pollen-deprived}}$ = 57). Source data are provided as a Source Data file.

### *2.8 Supplementary Note 8: Hazard function distribution selection*

Intercept-only parametric survival models were created by fitting survival data for each species to exponential, Weibull (Accelerated Failure Time [AFT]), Gompertz, gamma, lognormal, log-logistic, and generalised gamma distributions. For each species, the hazard functions from these (parametric) models were plotted alongside a smoothed estimate of the hazard function generated non-parametrically using the kernel density estimator from the R package *muhaz* v1.2.6.4[15]. The models could then be compared visually to assess how well they fit the data, as well as statistically using their Akaike information criterion (AIC) values.

Support for distributions was mixed, and the best-fitting distribution both visually and in terms of the lowest AIC value was different for different species (Figs. S6 and S7; Tables S9 and S10). This may be in part due to sample size, as some sources suggest a minimum of 100 individuals per group to distinguish between mortality functions[16], which was not true for all groups analysed, though groups did largely reach the suggested minimum of 50 individuals each to estimate age-specific mortality without introducing significant bias[17]. Choosing a distribution based on prior knowledge was also problematic, with few studies focusing on actuarial senescence in Lepidoptera; but those that do indicate that the Gompertz and Weibull models are among the best-fitting[18-20].

Considering all available information, the Gompertz distribution emerged as the best-supported overall. In particular, data from the pollen-manipulation experiment cohort, which is likely the best reflection of natural senescence, fit the Gompertz distribution extremely well (Fig. S7, Table S10). Survival data from the multi-species cognitive experiment cohort, while useful, is likely to more poorly reflect natural ageing patterns considering the strenuous conditions of this experiment, where survival was conditional upon learning a colour association, and butterflies were subjected to frequent handling and regular bouts of food deprivation. Therefore further statistical analysis for all cohorts was carried out using parametric survival models fit to a Gompertz distribution.

**Table S9:** Comparison of Akaike information criterion (AIC) values for parametric survival models fit to various mortality distributions for survival data from the multi-species cognitive experiment cohort. Distributions for each species are presented in order of most- to least-supported. $n_{A.\ vanillae}$ = 175, $n_{D.\ iulia}$ = 263, $n_{D.\ phaetusa}$ = 108, $n_{H.\ hecale}$ = 120, $n_{H.\ melpomene}$ = 66. Weibull (AFT) = Weibull (accelerated failure time).

| Species | Distribution | ΔAIC | Degrees of freedom |
|---|---|---|---|
| *A. vanillae* | Weibull (AFT) | 0 | 2 |
| | Gompertz | 0.62 | 2 |
| | Gamma | 1.01 | 2 |
| | Generalised gamma | 1.35 | 3 |
| | Lognormal | 9.65 | 2 |
| | Log-logistic | 20.41 | 2 |
| | Exponential | 26.87 | 1 |
| *D. phaetusa* | Generalised gamma | 0 | 3 |
| | Gompertz | 0.05 | 2 |
| | Weibull (AFT) | 21.89 | 2 |
| | Gamma | 35.03 | 2 |
| | Log-logistic | 55.07 | 2 |
| | Lognormal | 58.30 | 2 |
| | Exponential | 67.77 | 1 |
| *D. iulia* | Weibull (AFT) | 0 | 2 |
| | Generalised gamma | 0.57 | 3 |
| | Gamma | 5.40 | 2 |
| | Gompertz | 7.70 | 2 |
| | Lognormal | 32.36 | 2 |
| | Log-logistic | 33.65 | 2 |
| | Exponential | 60.19 | 1 |
| *H. hecale* | Generalised gamma | 0 | 3 |
| | Gompertz | 8.12 | 2 |
| | Exponential | 18.53 | 1 |
| | Weibull (AFT) | 19.61 | 2 |
| | Gamma | 20.35 | 2 |
| | Log-logistic | 32.34 | 2 |
| | Lognormal | 33.57 | 2 |
| *H. melpomene* | Weibull (AFT) | 0 | 2 |
| | Gamma | 0.61 | 2 |
| | Log-logistic | 1.62 | 2 |
| | Generalised gamma | 1.98 | 3 |
| | Gompertz | 2.44 | 2 |
| | Lognormal | 3.53 | 2 |
| | Exponential | 15.01 | 1 |

**Table S10:** Comparison of Akaike information criterion (AIC) values for parametric survival models fit to various mortality distributions for survival data from the pollen-manipulation experiment cohort. Distributions for each species are presented in order of most- to least-supported. $n_{H.\ hecale}$ = 96, $n_{D.\ iulia}$ = 116. Weibull (AFT) = Weibull (accelerated failure time).

| Species | Distribution | ΔAIC | Degrees of freedom |
|---|---|---|---|
| *H. hecale* | Gompertz | 0 | 2 |
| | Generalised gamma | 3.51 | 3 |
| | Weibull (AFT) | 9.87 | 2 |
| | Gamma | 15.97 | 2 |
| | Exponential | 24.01 | 1 |
| | Log-logistic | 28.46 | 2 |
| | Lognormal | 42.53 | 2 |
| *D. iulia* | Gompertz | 0 | 2 |
| | Generalised gamma | 4.04 | 3 |
| | Weibull (AFT) | 11.21 | 2 |
| | Gamma | 24.09 | 2 |
| | Log-logistic | 31.86 | 2 |
| | Lognormal | 49.12 | 2 |
| | Exponential | 62.77 | 1 |

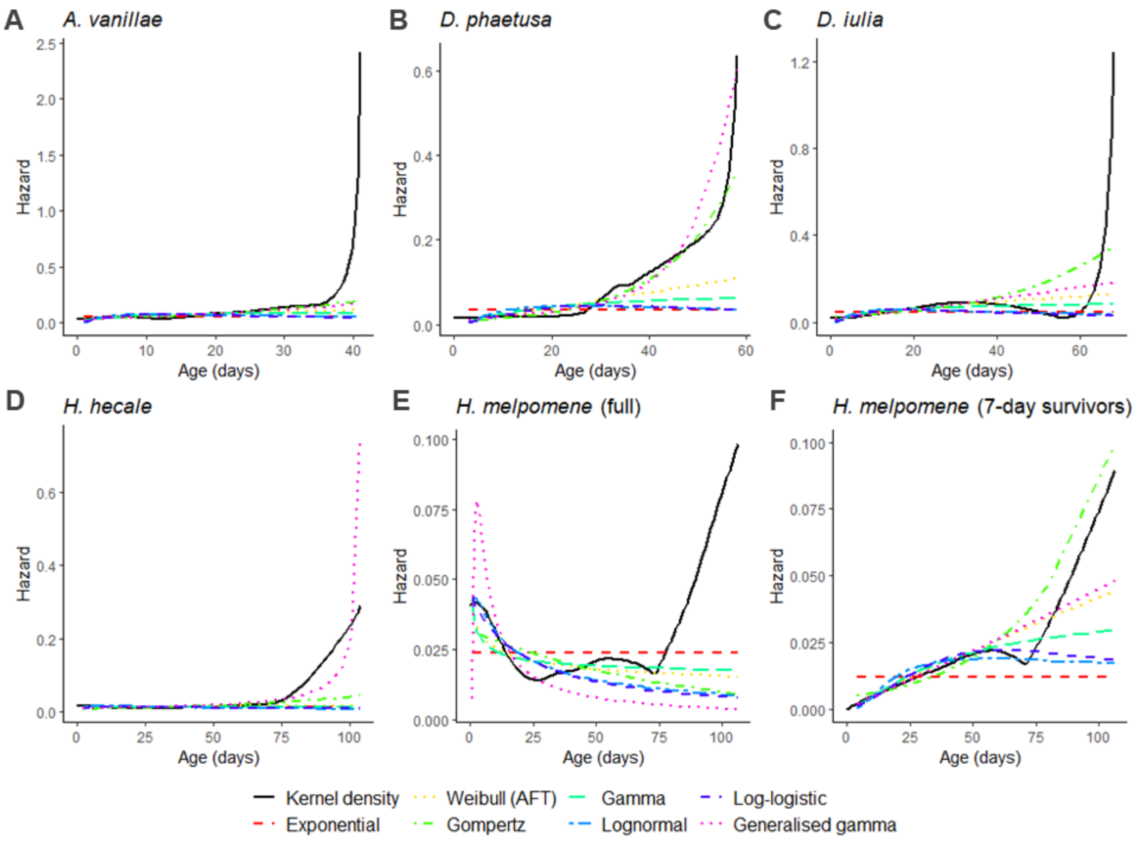


**Fig. S6: Hazard rate curves fit to various mortality distributions for the multi-species cognitive experiment cohort.** The kernel density estimate is used to compare fit of the various models to the actual data from the experiment. Both the full *H. melpomene* dataset (**E**; *n* = 103) and the 7-day survivors subset (**F;** *n* = 66) are plotted to justify the use of the latter for further statistical analysis (see Supplementary Note 10). $n_{A.\ vanillae}$ = 175, $n_{D.\ iulia}$ = 263, $n_{D.\ phaetusa}$ = 108, $n_{H.\ hecale}$ = 120. Source data are provided as a Source Data file.

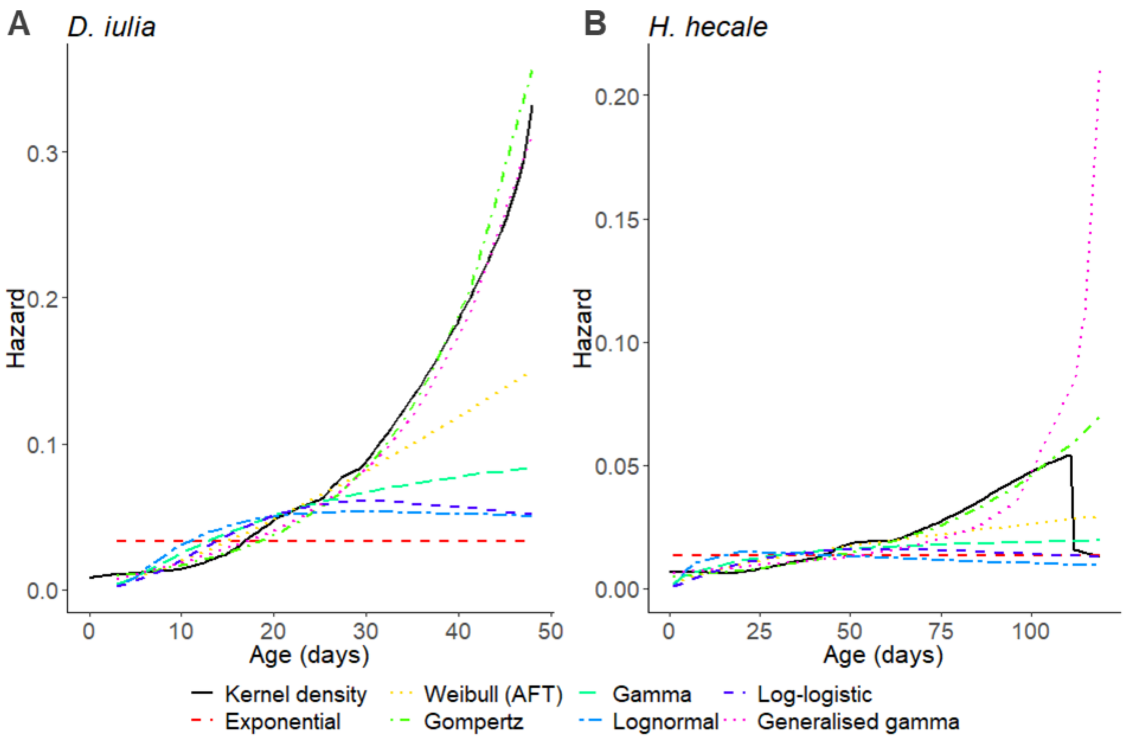


**Fig. S7: Hazard rate curves fit to various mortality distributions for butterflies in the pollen-manipulation experiment cohort.** The kernel density estimate is used to compare fit of the various models to the actual data from the experiment. $n_{D.\ iulia}$ = 116, $n_{H.\ hecale}$ = 96. Source data are provided as a Source Data file.

### *2.9 Supplementary Note 9: Comparison of indices of actuarial senescence*

While the use of the $\beta$ parameter from Gompertz models is widely used as a scaleless index of actuarial senescence (i.e. rate of ageing), there has been some debate about this, with some authors instead proposing the use of an index that combines the $\alpha$ and $\beta$ parameters, taking the root of their product[21]. For completeness, we include this index below in revised versions of Tables 2, 3, and 4 (Tables S11, S12, and S13, respectively), to allow for comparison between these indices. Rate of ageing as measured by this combined index ($\sqrt{\alpha\beta}$) is still almost exclusively lower in *Heliconius* species than in the Heliconiini outgroups, with the exception of *Eueides isabella*, which has a rate of ageing lower than several *Heliconius* species as estimated from the semi-natural "mark-release-recapture" cohort. To aid assessment, species are presented in descending order of the combined index ($\sqrt{\alpha\beta}$) in Tables S11 and S12.

**Table S11: Ageing parameters for the multi-species cognitive experiment cohort.**

| Species | Feeding habit | Median lifespan (days) | Maximum lifespan (days) | Baseline mortality ($\alpha$) | Rate of ageing ($\beta$) | Rate of ageing ($\sqrt{\alpha\beta}$) |
|---|---|---|---|---|---|---|
| *Agraulis vanillae* | non-pollen-feeding | 17 | 41 | 0.033 | 0.044 | 0.038 |
| *Dryas iulia* | non-pollen-feeding | 21 | 68 | 0.024 | 0.040 | 0.031 |
| *Dryadula phaetusa* | non-pollen-feeding | 34 | 58 | 0.0075 | 0.067 | 0.022 |
| *Heliconius melpomene* | pollen-feeding | 52 | 106 | 0.0050 | 0.029 | 0.012 |
| *Heliconius hecale* | pollen-feeding | 61 | 104 | 0.0070 | 0.018 | 0.011 |

**Table S12: Ageing parameters for the semi-natural "mark-release-recapture" cohort.**

| Species | Feeding habit | Median lifespan (days) | Maximum lifespan (days) | Baseline mortality ($\alpha$) | Rate of ageing ($\beta$) | Rate of ageing ($\sqrt{\alpha\beta}$) |
|---|---|---|---|---|---|---|
| *Dione juno* | non-pollen-feeding | 4.03 | 11 | 0.86 | 0.15 | 0.36 |
| *Agraulis vanillae* | non-pollen-feeding | 6.27 | 11 | 0.42 | 0.16 | 0.26 |
| *Dryadula phaetusa* | non-pollen-feeding | 8.93 | 17 | 0.36 | 0.086 | 0.18 |
| *Dryas iulia* | non-pollen-feeding | 3.33 | 25 | 1.32 | 0.025 | 0.18 |

| | | | | | | |
|---|---|---|---|---|---|---|
| *Heliconius ismenius* | pollen-feeding | 15.93 | 24 | 0.15 | 0.074 | 0.11 |
| *Heliconius doris* | pollen-feeding | 17.68 | 35 | 0.14 | 0.07 | 0.099 |
| *Heliconius hewitsoni* | pollen-feeding | 17.68 | 41 | 0.17 | 0.046 | 0.088 |
| *Eueides isabella* | non-pollen-feeding | 4.03 | 50 | 1.18 | 0.0047 | 0.075 |
| *Heliconius cydno* | pollen-feeding | 19.78 | 55 | 0.19 | 0.026 | 0.070 |
| *Heliconius pachinus* | pollen-feeding | 24.33 | 58 | 0.13 | 0.033 | 0.066 |
| *Heliconius atthis* | pollen-feeding | 16.63 | 53 | 0.25 | 0.016 | 0.063 |
| *Heliconius hecale* | pollen-feeding | 18.73 | 78 | 0.23 | 0.013 | 0.055 |
| *Heliconius numata* | pollen-feeding | 23.28 | 106 | 0.17 | 0.016 | 0.052 |
| *Heliconius melpomene* | pollen-feeding | 30.28 | 102 | 0.14 | 0.0087 | 0.035 |
| *Heliconius erato* | pollen-feeding | 33.08 | 94 | 0.12 | 0.0092 | 0.033 |
| *Heliconius sara* | pollen-feeding | 24.33 | 107 | 0.19 | 0.0053 | 0.032 |
| *Heliconius sapho* | pollen-feeding | 32.73 | 143 | 0.13 | 0.0056 | 0.027 |

**Table S13: Ageing parameters for the pollen-manipulation experiment cohort.**

| **Species** | **Diet treatment** | **Median lifespan (days)** | **Maximum lifespan (days)** | **Baseline mortality ($\alpha$)** | **Rate of ageing ($\beta$)** | **Rate of ageing ($\sqrt{\alpha\beta}$)** |
|---|---|---|---|---|---|---|
| *Heliconius hecale* | pollen-fed | 63 | 119 | 0.0044 | 0.025 | 0.011 |
| | pollen-deprived | 47 | 106 | 0.0087 | 0.025 | 0.015 |
| *Dryas iulia* | pollen-fed | 27 | 48 | 0.0077 | 0.081 | 0.025 |
| | pollen-deprived | 29 | 50 | 0.0071 | 0.081 | 0.024 |

### *2.10 Supplementary Note 10:* **H. melpomene** ***early deaths exclusion***

The learning and memory assays performed on individuals from the multi-species cognitive experiment cohort necessitated frequent handling of the butterflies, as well as regular bouts of food-deprivation during the colour choice assays, introducing stressors which likely impacted survival. One example of this was the high early mortality demonstrated in *H. melpomene*, with a substantial number of individuals ($n$ = 37; 35.92% of the 103 total butterflies) dying in the first week. Inclusion of this early spike in mortality in survival analysis greatly biased estimates of median survival time for this species, and also produced uninformative estimates of other ageing parameters, including a conclusion of negative senescence which is not supported by any other data in this paper (Fig. S8). Visual inspection confirmed that removal of these early deaths allowed a much better fit of the data to standard parametric survival distributions (Fig. S6). This incongruity fits with the hypothesis that the high early mortality observed was likely due to an inability to adapt to the contrived conditions in the experimental cages, rather than a reflection of senescence in natural conditions. Therefore only individuals of *H. melpomene* that survived for longer than a week were included in further statistical analysis ($n$ = 66). Subsetting the other species in this dataset to just individuals who survived the first 7 days of the experiments resulted in qualitatively similar findings (Fig. 1, Fig. S8). However, there was no support for performing this exclusion in these species (Fig. S6), and so further statistical analysis for all other species was carried out on the full group.

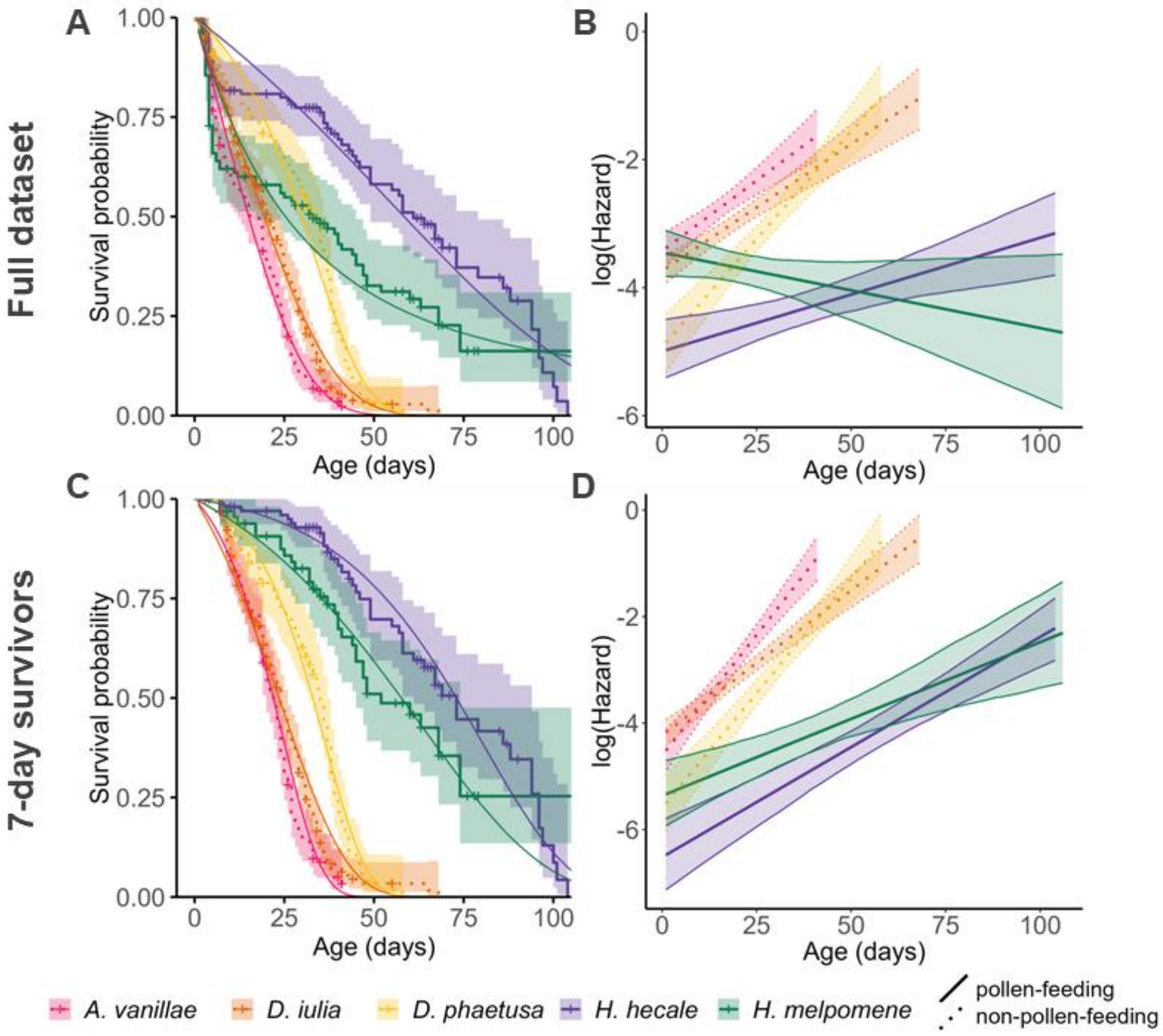


**Fig. S8: Survival and log(Hazard) curves for butterflies in the multi-species cognitive experiment cohort for (A-B) the full dataset and (C-D) only those individuals surviving at least to 7 days. A** and **C**: Kaplan-Meier survival estimates (stepped lines) and 95% confidence intervals (shaded regions) overlaid with the corresponding parametric survival curves (smooth curves) for the multi-species cognitive experiment cohort. "+" indicates a censored data point. **B** and **D**: Model-estimated log(Hazard) (equivalent to log[likelihood of death]) curves (lines) and 95% confidence intervals (shaded regions) resulting from the transformation of the corresponding parametric survival function shown in **A** and **C** to the hazard function. An increase in the intercept on this graph represents an increase in baseline mortality ($\alpha$); an increase in the slope represents an increase in the rate of ageing ($\beta$). $n_{A.\ vanillae}$ = 175, $n_{D.\ iulia}$ = 263, $n_{D.\ phaetusa}$ = 108, $n_{H.\ hecale}$ = 120, $n_{H.\ melpomene:\ \mathbf{A,\ B}}$ = 103, $n_{H.\ melpomene:\ \mathbf{C,\ D}}$ = 66. The unlikely conclusion of negative senescence in *H. melpomene* as demonstrated in **B** as well as the poor fit of survival data for this species to any hazard function as shown in Fig. S6 provided justification for the subsetting of the dataset for this species to only individuals surviving at least to 7 days for further analysis in this chapter. There was not similar support for performing this exclusion on any other species in this dataset, but **C-D** are plotted to show that doing so results in qualitatively similar conclusions (comparison with Fig. 1). Source data are provided as a Source Data file.

### *2.11 Supplementary Note 11: Functional senescence results without longevity included as a covariate*

Selective disappearance is a phenomenon whereby compositional differences arise between age classes as a result of phenotypically “poorer” individuals dying earlier[22]. This can result in the appearance of negligible (functional) senescence, simply because the remaining older individuals are primarily composed of the phenotypically “better” individuals that managed to survive longer. This is usually corrected for in analysis either by statistically separating within- from between-individual effects[23], or by including individual longevity as a covariate, allowing for unbiased estimates of individual senescence[24]. As we were primarily interested in within-individual effects related to senescence, and eager to avoid any potential biases introduced by selective disappearance, we included individual longevity as a covariate in our functional senescence models. However, models without this covariate resulted in qualitatively similar findings. We present a summary of these results below for comparison with corresponding age-related statistics from the main Results section, as well as Supplementary Note 7 (for flight behaviour results).

All butterflies showed an age-related decline in body mass, but there was a significant interaction between species, diet, and age ($F_{1,\,377.32} = 9.53$, $p = 0.002$). Pollen-deprived *H. hecale* showed a steeper body mass decline with age than the pollen-fed group, an effect which persisted across the full *H. hecale* lifespan ($F_{1,\,300.61} = 63.65$, $p < 0.001$). In *D. iulia* there was no difference in rate of body mass decline with age between diet treatments ($\chi^2_1 = 0.53$, $p = 0.467$). Across both species, there was also a significant interaction between age and sex, with body mass in males declining more steeply with age than in females ($F_{1,\,377.58} = 18.13$, $p < 0.001$), an effect which persisted across the full *H. hecale* lifespan ($F_{1,\,301.41} = 4.18$, $p = 0.042$).

Grip strength weakened with age in *D. iulia* ($F_{1,\,126.82} = 4.79$, $p = 0.030$), with week 5 butterflies pulling an estimated 0.26g less than week 1 butterflies, a reduction of 19.73%. However, this was not true of *H. hecale*, which did not show signs of weakening grip strength with age, even across the full lifespan ($F_{1,\,283.47} = 0.75$, $p = 0.386$). Diet did not have an impact on grip strength in *D. iulia* ($F_{1,\,86.42} = 0.97$, $p = 0.326$), but in *H. hecale*, pollen-deprived butterflies were weaker than the pollen-fed group ($F_{1,\,77.62} = 9.99$, $p = 0.002$), pulling an estimated 0.32g less, a reduction of 15.30% compared to the pollen-fed group. However, there was no interaction between age and diet in *H. hecale* ($\chi^2_1 = 2.49$, $p = 0.115$), meaning that the impact of pollen-deprivation was consistent across the full lifespan.

Age was still not found to be a significant predictor of time spent in active flight in *H. hecale* ($\chi^2_1$ = 1.04, $p$ = 0.309). In *D. iulia*, there was an interaction between age, sex, and diet ($\chi^2_1$ = 4.15, $p$ = 0.042) such that in females, pollen-fed butterflies spent more time in active flight as they aged, whereas pollen-deprived butterflies spent less time in active flight as they aged.

## References


1. Lane, S.J., Frankino, W.A., Elekonich, M.M. & Roberts, S.P. The effects of age and lifetime flight behavior on flight capacity in *Drosophila melanogaster*. *J. Exp. Biol.* **217**, 1437–1443 (2014).
2. Sohal, R.S. Aging in Insects. in *Biochemistry* (eds. Kerkut, G.A. & Gilbert, L.I.) 595–631 (Pergamon, 1985).
3. Åhman, M. & Karlsson, B. Flight endurance in relation to adult age in the green-veined white butterfly *Pieris napi*. *Ecol. Entomol.* **34**, 783–787 (2009).
4. Kubinec, R. Ordered Beta Regression: A Parsimonious, Well-Fitting Model for Continuous Data with Lower and Upper Bounds. *Polit. Anal.* 1–18 (2022).
5. Douma, J.C. & Weedon, J.T. Analysing continuous proportions in ecology and evolution: A practical introduction to beta and Dirichlet regression. *Methods Ecol. Evol.* **10**, 1412–1430 (2019).
6. Brooks, M.E. *et al.* glmmTMB balances speed and flexibility among packages for zero-inflated generalized linear mixed modeling. *R J.* **9**, 378–400 (2017).
7. Kelson, R. Searching for Methuselah: Butterfly Longevity Revisited. in *Proceedings of the 2008 Invertebrates in Education and Conservation Conference* 51–57 (Sonoran Arthropod Studies Institute, 2008).
8. Watts, J.R. Longevity studies in a tropical conservatory: are you getting your money's worth? in *Proceedings of the 2004 Invertebrates in Education and Conservation Conference* 39–45 (Sonoran Arthropod Studies Institute, 2004).
9. Davis, A.K., Smith, F.M. & Ballew, A.M. A poor substitute for the real thing: captive-reared monarch butterflies are weaker, paler and have less elongated wings than wild migrants. *Biol. Lett.* **16**, 20190922 (2020).
10. Lailvaux, S.P., Hathway, J., Pomfret, J. & Knell, R.J. Horn size predicts physical performance in the beetle *Euoniticellus intermedius* (Coleoptera: Scarabaeidae). *Funct. Ecol.* **19**, 632–639 (2005).
11. Bohannon, R.W. Grip Strength: An Indispensable Biomarker For Older Adults. *Clin. Interv. Aging* **14**, 1681–1691 (2019).
12. Dalbosco Dell'Aglio, D., McMillan, W. O. & Montgomery, S. H. Using motion-detection cameras to monitor foraging behaviour of individual butterflies. *Ecol. Evol.* **14**, (2024).
13. Niitepõld, K. & Boggs, C.L. Carry-over effects of larval food stress on adult energetics and life history in a nectar-feeding butterfly. *Ecol. Entomol.* **47**, 391–399 (2022).
14. Niitepõld, K., Perez, A. & Boggs, C.L. Aging, life span, and energetics under adult dietary restriction in lepidoptera. *Physiol. Biochem. Zool.* **87**, 684–694 (2014).
15. Hess, K. & Gentleman, R. muhaz: Hazard Function Estimation in Survival Analysis. (2021).
16. Wilson, D.L. The analysis of survival (mortality) data: Fitting Gompertz, Weibull, and logistic functions. *Mech. Ageing Dev.* **74**, 15–33 (1994).
17. Pletcher, S.D. Model fitting and hypothesis testing for age-specific mortality data. *J. Evol. Biol.* **12**, 430–439 (1999).
18. Carroll, J., Korshikov, E. & Sherratt, T.N. Post-reproductive senescence in moths as a consequence of kin selection: Blest's theory revisited. *Biol. J. Linn. Soc.* **104**, 633–641 (2011).
19. Carroll, J. & Sherratt, T.N. Actuarial senescence in laboratory and field populations of Lepidoptera. *Ecol. Entomol.* **42**, 675–679 (2017).
20. Sielezniew, M., Kostro-Ambroziak, A. & Kőrösi, Á. Sexual differences in age-dependent survival and life span of adults in a natural butterfly population. *Sci. Rep.* **10**, (2020).

21. Ricklefs, R.E. & Scheuerlein, A. Biological Implications of the Weibull and Gompertz Models of Aging. *J. Gerontol. A Biol. Sci. Med. Sci.* **57**, B69–B76 (2002).
22. Nussey, D.H. *et al*. Patterns of body mass senescence and selective disappearance differ among three species of free-living ungulates. *Ecology* **92**, 1936–1947 (2011).
23. Van De Pol, M. & Verhulst, S. Age-Dependent Traits: A New Statistical Model to Separate Within- and Between-Individual Effects. *Am. Nat.* **167**, 766–773 (2006).
24. Hayward, A.D. *et al.* Reproductive senescence in female Soay sheep: variation across traits and contributions of individual ageing and selective disappearance. *Funct. Ecol.* **27**, 184–195 (2013).
25. Young, F.J. *et al*. Enhanced long-term memory and increased mushroom body plasticity in Heliconius butterflies. *iScience* **27**, 108949 (2024).
