# Supplementary material for: Evolution of increased longevity and slowed ageing in a genus of tropical butterfly
Source: Nat Commun. 2026 Jun 16;17:5077. doi: 10.1038/s41467-026-73635-7 (PMC13272878; doi:10.1038/s41467-026-73635-7)
Supplement: Supplementary file 2 — Description of Additional Supplementary Files [file 41467_2026_73635_MOESM2_ESM.pdf]

**Description of Additional Supplementary Files**

**File Name:** Supplementary Data 1
**Description:** Full list of maximum reported lifespan records for all butterflies (including many non-Heliconiini species) in Richard Kelson's long-running Butterfly Habitat census.

**File Name:** Supplementary Data 2
**Description:** Full list of all available maximum reported lifespan records for Heliconiini species.

**File Name:** Supplementary Data 3
**Description:** Survival data for the multi-species cognitive experiment cohort.

**File Name:** Supplementary Data 4
**Description:** Individual data for the semi-natural "mark-release-recapture" cohort.

**File Name:** Supplementary Data 5
**Description:** Sightings data for the semi-natural "mark-release-recapture" cohort.

**File Name:** Supplementary Data 6
**Description:** Survival data for the pollen-manipulation experiment cohort.

**File Name:** Supplementary Data 7
**Description:** Weight data for the pollen-manipulation experiment cohort.

**File Name:** Supplementary Data 8
**Description:** Grip strength data for the pollen-manipulation experiment cohort.

**File Name:** Supplementary Data 9
**Description:** Flight behaviour data for the pollen-manipulation experiment cohort.
