# Supplementary material for: Evolution of increased longevity and slowed ageing in a genus of tropical butterfly
Source: Nat Commun. 2026 Jun 16;17:5077. doi: 10.1038/s41467-026-73635-7 (PMC13272878; doi:10.1038/s41467-026-73635-7)
Supplement: Supplementary file 4 — Reporting Summary [file 41467_2026_73635_MOESM4_ESM.pdf]

nature portfolio

Corresponding author(s): Jessica Foley
Stephen H. Montgomery

Last updated by author(s): Jan 5, 2026

Reporting Summary

Nature Portfolio wishes to improve the reproducibility of the work that we publish. This form provides structure for consistency and transparency in reporting. For further information on Nature Portfolio policies, see our Editorial Policies and the Editorial Policy Checklist.

Statistics

For all statistical analyses, confirm that the following items are present in the figure legend, table legend, main text, or Methods section.

n/a	Confirmed	
☐	☒	The exact sample size (*n*) for each experimental group/condition, given as a discrete number and unit of measurement
☐	☒	A statement on whether measurements were taken from distinct samples or whether the same sample was measured repeatedly
☐	☒	The statistical test(s) used AND whether they are one- or two-sided<br>*Only common tests should be described solely by name; describe more complex techniques in the Methods section.*
☐	☒	A description of all covariates tested
☐	☒	A description of any assumptions or corrections, such as tests of normality and adjustment for multiple comparisons
☐	☒	A full description of the statistical parameters including central tendency (e.g. means) or other basic estimates (e.g. regression coefficient) AND variation (e.g. standard deviation) or associated estimates of uncertainty (e.g. confidence intervals)
☐	☒	For null hypothesis testing, the test statistic (e.g. *F*, *t*, *r*) with confidence intervals, effect sizes, degrees of freedom and *P* value noted<br>*Give P values as exact values whenever suitable.*
☒	☐	For Bayesian analysis, information on the choice of priors and Markov chain Monte Carlo settings
☒	☐	For hierarchical and complex designs, identification of the appropriate level for tests and full reporting of outcomes
☐	☒	Estimates of effect sizes (e.g. Cohen's *d*, Pearson's *r*), indicating how they were calculated

Our web collection on statistics for biologists contains articles on many of the points above.

Software and code

Policy information about availability of computer code

Data collection	No software was used for data collection.
Data analysis	R v4.3.1; R packages used included survival v3.5-5, coxme v2.2-18, flexsurv v2.2.2, BaSTA v1.9.5, phytools v2.3-0, and lme4 v1.1-34.

For manuscripts utilizing custom algorithms or software that are central to the research but not yet described in published literature, software must be made available to editors and reviewers. We strongly encourage code deposition in a community repository (e.g. GitHub). See the Nature Portfolio guidelines for submitting code & software for further information.

Data

Policy information about availability of data

All manuscripts must include a data availability statement. This statement should provide the following information, where applicable:

- Accession codes, unique identifiers, or web links for publicly available datasets
- A description of any restrictions on data availability
- For clinical datasets or third party data, please ensure that the statement adheres to our policy

All data generated in this study and R scripts used for analysis of this data have been deposited in an associated figshare repository and may be accessed here: https://doi.org/10.6084/m9.figshare.31081597. This includes all lifespan data from Mr. Kelson's butterfly house census, maximum lifespan records from the literature and other commercial butterfly houses, as well as lifespan and trait data for all other butterflies in this study. Source data are provided with this paper.

Research involving human participants, their data, or biological material

Policy information about studies with human participants or human data. See also policy information about sex, gender (identity/presentation), and sexual orientation and race, ethnicity and racism.

Reporting on sex and gender	*Use the terms sex (biological attribute) and gender (shaped by social and cultural circumstances) carefully in order to avoid confusing both terms. Indicate if findings apply to only one sex or gender; describe whether sex and gender were considered in study design; whether sex and/or gender was determined based on self-reporting or assigned and methods used. Provide in the source data disaggregated sex and gender data, where this information has been collected, and if consent has been obtained for sharing of individual-level data; provide overall numbers in this Reporting Summary. Please state if this information has not been collected. Report sex- and gender-based analyses where performed, justify reasons for lack of sex- and gender-based analysis.*
Reporting on race, ethnicity, or other socially relevant groupings	*Please specify the socially constructed or socially relevant categorization variable(s) used in your manuscript and explain why they were used. Please note that such variables should not be used as proxies for other socially constructed/relevant variables (for example, race or ethnicity should not be used as a proxy for socioeconomic status). Provide clear definitions of the relevant terms used, how they were provided (by the participants/respondents, the researchers, or third parties), and the method(s) used to classify people into the different categories (e.g. self-report, census or administrative data, social media data, etc.) Please provide details about how you controlled for confounding variables in your analyses.*
Population characteristics	*Describe the covariate-relevant population characteristics of the human research participants (e.g. age, genotypic information, past and current diagnosis and treatment categories). If you filled out the behavioural & social sciences study design questions and have nothing to add here, write "See above."*
Recruitment	*Describe how participants were recruited. Outline any potential self-selection bias or other biases that may be present and how these are likely to impact results.*
Ethics oversight	*Identify the organization(s) that approved the study protocol.*

Note that full information on the approval of the study protocol must also be provided in the manuscript.

Field-specific reporting

Please select the one below that is the best fit for your research. If you are not sure, read the appropriate sections before making your selection.

☐ Life sciences ☐ Behavioural & social sciences ☒ Ecological, evolutionary & environmental sciences

For a reference copy of the document with all sections, see nature.com/documents/nr-reporting-summary-flat.pdf

Ecological, evolutionary & environmental sciences study design

All studies must disclose on these points even when the disclosure is negative.

Study description

The study consisted of 3 cohorts of butterflies; a repurposed dataset from a previous multi-species cognitive experiment (5x species), a novel "mark-release recapture" cohort (20x species), and a novel pollen-manipulation cohort (2x species). Both sexes were represented and approximately equally distributed for all cohorts. Below I list sample sizes and design for each cohort:

Multi-species cognitive experiment:

This comprised of semi-parametric survival models for each species for which the only predictor tested was sex. When sex was not found to be a significant predictor of survival these were followed by inter-species parametric survival models for which the only predictor tested was species. Outputs from these models were then compared statistically to find differences related to feeding habit.

In total, survival data from both sexes was analysed for 175 individuals of Agraulis vanillae (12 censored observations), 263 individuals of Dryas iulia (38 censored observations), 108 individuals of Dryadula phaetusa (10 censored observations), 120 individuals of Heliconius hecale (56 censored observations), and 103 individuals of Heliconius melpomene (31 censored observations).

Mark-release-recapture cohort:

This comprised of parametric survival models for each species, the outputs from which were then compared statistically to find differences related to feeding habit. In total, 959 butterflies of 20 different Heliconiini species were released into the cage, including: Agraulis vanillae (n = 12), Dryas iulia (n = 34), Dione juno (n = 45), Dryaula phaetusa (n = 30), Eueides isabella (n = 59), Heliconius atthis (n = 33), Heliconius charithonia (n = 3), Heliconius cydno (n = 19), Heliconius doris (n = 32), Heliconius erato (n = 46), Heliconius hewitsoni (n = 15), Heliconius hecale (n = 47), Heliconius himera (n = 2), Heliconius ismenius (n = 20), Heliconius melpomene (n = 285), Heliconius numata (n = 94), Heliconius pachinus (n = 19), Heliconius sapho (n = 81), Heliconius sara (n = 70), and Philaethria dido (n = 13).

Pollen-manipulation cohort:

This comprised of semi-parametric survival models for each species with candidate predictors including eclosion mass, diet, sex, and the two-way diet:sex interaction. These were followed by parametric survival models for each species individually with diet as a candidate predictor, and then both species together with diet and species as candidate predictors.

For the functional senescence analyses, all single-species “full” models included diet, age, sex, and their three-way interaction as candidate predictors. All interspecific “full” models included species, diet, age, and their three-way interaction as candidate predictors, as well as two-way interactions between species and any candidate predictors found to be significant in the single-species

models. All models also included longevity as a candidate predictor, to account for the possibility of selective disappearance and ensure unbiased estimates of the effect of age. For body mass, additional candidate predictors included assay time (measured as the fraction of the day elapsed since midnight). For grip strength, additional candidate predictors included assay time and eclosion mass (measured in grams [g]). For flight behaviour, additional candidate predictors included forewing length (measured in millimetres [mm]), illuminance (measured in lux), assay time, whether it was raining, and whether the butterfly had an intact wing apex (used as an index of wing-wear).

In total, data from both sexes was collected across the lifespan of 96 individuals of Heliconius hecale (47 pollen-fed, 49 pollen-deprived; 26 censored observations) and 116 individuals of Dryas iulia (57 pollen-fed, 57 pollen-deprived; 36 censored observations).

Research sample

The sample consisted of a wide range of species from across the Heliconiini tribe, chosen to assist with a comprehensive investigation of the degree and extent of the lifespan extension of species of the pollen-feeding Heliconius genus over their non-pollen-feeding relatives in the Heliconiini tribe. These species include: Agraulis vanillae, Dryas iulia, Dione juno, Dryadula phaetusa, Eueides isabella, Heliconius atthis, Heliconius charithonia, Heliconius cydno, Heliconius doris, Heliconius erato, Heliconius hewitsoni, Heliconius hecale, Heliconius himera, Heliconius ismenius, Heliconius melpomene, Heliconius numata, Heliconius pachinus, Heliconius sapho, Heliconius sara, and Philaethria dido.

More in-depth analyses were conducted on H. hecale and D. iulia as representative longer- and shorter-lived species largely for their local abundance and ease of rearing, but this choice is validated by the demonstration of their similar baseline mortality but distinctive rates of ageing as shown in Figure 2.

Sampling strategy

Sampling procedures consisted of recording death or last-seen-alive dates for survival analyses, and measuring body mass / grip strength / flight behaviour for functional senescence analyses. We reared as many individuals of each species as possible for these experiments within the 10-month field season, and so sample size in theory had no upper limit, but was restricted by rearing capacity. We were aiming to reach the suggested minimum of 50 individuals in each treatment group to estimate age-specific mortality without introducing significant bias (Pletcher, 1999), which we largely succeeded in doing.

Data collection

Data was primarily collected by Jessica Foley, Josie McPherson, Made Roger, Cruz Batista, and Fletcher J. Young. For survival data from the insectary cohorts, cages were checked daily for dead individuals, death dates were recorded, and missing or predated individuals were censored at the age at which they were last seen alive. For survival data for the mark-release-recapture cohort, approximately 15 minutes were spent patrolling the cage and recording the IDs of any butterflies visually identified and alive on that date. For the functional senescence assays, individuals were removed from their cages at approximately 10:00, and subjected first to the flight behaviour assay, followed by the body mass and grip strength assays. The flight behaviour assays were performed in the outdoor insectaries, and the body mass and grip strength assays were performed in the neighbouring laboratory. Testing generally concluded by 13:00, after which butterflies were returned to their respective cages.

Timing and spatial scale

Survival data for the multi-species cognitive experiment cohort was collected between January-May 2019 (for H. melpomene, H. hecale, D. phaetusa, and A. vanillae), or between January-November 2022 (for D. iulia). This is because this for all species aside from D. iulia, this data was repurposed from a prior field season conducted by a colleague in the lab (Fletcher J. Young); however, his D. iulia data could not be used for survival analysis as all individuals of this species were dissecting upon completing the experiment, thus violating the assumption of uninformative censoring for survival analysis. All other data was collected in one field season between January-November 2022. All data was collected at the STRI Heliconius insectaries in Gamboa, Panama.

Sampling for survival data was conducted daily. Sampling for functional senescence assays was conducted every 2 weeks for each butterfly. It was not possible to sample any more frequently than this due to the number of experimental individuals (212) and the length of time required to conduct the battery of assays.

Data exclusions

Some data were excluded from final analyses, and details of this exclusion (which was decided after conducting analyses) are reported in Supplementary Note 8. We recommend reviewing this to see the figures referenced below, which are particularly critical in justifying this exclusion.

H. melpomene early deaths exclusion:

The learning and memory assays performed on individuals from the multi-species cognitive experiment cohort necessitated frequent handling of the butterflies, as well as regular bouts of food-deprivation during the colour choice assays, introducing stressors which likely impacted survival. One example of this was the high early mortality demonstrated in H. melpomene, with a substantial number of individuals (n = 37; 35.92%) dying in the first week. Inclusion of this early spike in mortality in survival analysis greatly biased estimates of median survival time for this species, and also produced uninformative estimates of other ageing parameters, including a conclusion of negative senescence which is not supported by any other data in this paper (Fig. S7). Visual inspection confirmed that removal of these early deaths allowed a much better fit of the data to standard parametric survival distributions (Fig. S4). This incongruity fits with the hypothesis that the high early mortality observed was likely due to an inability to adapt to the contrived conditions in the experimental cages, rather than a reflection of senescence in natural conditions. Therefore only individuals of H. melpomene that survived for longer than a week were included in further statistical analysis (n = 66, with 31 censored observations). Subsetting the other species in this dataset to just individuals who survived the first 7 days of the experiments resulted in qualitatively similar findings (Fig. 1, Fig. S7). However, there was no support for performing this exclusion in these species (Fig. S4), and so further statistical analysis for all other species was carried out on the full group.

Reproducibility

Every attempt was made to ensure accurate data collection, to control for confounding variables, and to assess all findings in an unbiased framework. However, considering the time- and resource-intensive data collection period (10 months in the field with very few days off), we did not attempt to replicate our results. We would welcome and happily assist any groups who would like to do so. All R scripts used for analysis have been deposited in the associated figshare repository for any individuals interested in reproducing our analyses and may be accessed here: https://doi.org/10.6084/m9.figshare.31081597.

Randomization

Individuals were randomly allocated into groups (pollen-deprived/pollen-fed) using a coin-toss.

Blinding

Blinding was largely not possible for survival analyses because species were easily identified, and different diet treatments were maintained in separate cages. However, for the functional senescence assays, individuals were assayed in a random order, and so observers were at least blind to diet treatment as it was impossible to tell based on their physical appearance which butterflies had been allocated to which treatment.

Did the study involve field work? [x] Yes [ ] No

Field work, collection and transport

Field conditions	The majority of the data was collected in Gamboa, Panama between January and November 2022. The traversed the dry (December-April) and rainy (May-November) seasons in Panama, and with rainfall averaging 2235mm for the year. Temperatures averaged 25.3'C.
Location	The sampling and experiments were conducted at or near Gamboa, Panama, latitude: 9.1167, longitude: -79.7000, elevation ~40 metres.
Access & import/export	All plants and animals were collected with permission from the Ministerio del Ambiente in Panama, under collection permits SE/A-14-18 and SE/A-82-19.
Disturbance	Butterfly sampling was never very intensive (in total collecting around 60 females and 40 males across the 10-month field season from a relatively wide local radius) and thus we believe that our study did not cause any meaningful disturbance to local ecosystems.

Reporting for specific materials, systems and methods

We require information from authors about some types of materials, experimental systems and methods used in many studies. Here, indicate whether each material, system or method listed is relevant to your study. If you are not sure if a list item applies to your research, read the appropriate section before selecting a response.

Materials & experimental systems

n/a	Involved in the study	
[x]	[ ]	Antibodies
[x]	[ ]	Eukaryotic cell lines
[x]	[ ]	Palaeontology and archaeology
[ ]	[x]	Animals and other organisms
[x]	[ ]	Clinical data
[x]	[ ]	Dual use research of concern
[ ]	[x]	Plants

Methods

n/a	Involved in the study	
[x]	[ ]	ChIP-seq
[x]	[ ]	Flow cytometry
[x]	[ ]	MRI-based neuroimaging

Animals and other research organisms

Policy information about studies involving animals; ARRIVE guidelines recommended for reporting animal research, and Sex and Gender in Research

Laboratory animals	The study did not involve laboratory animals.
Wild animals	For the semi-natural mark-release-recapture experiment, stocks of non-Panamanian species were previously established by STRI scientists prior to our arrival, often from pupae from butterfly farms across Central and South America. For all other experiments, wild-caught individuals of unknown age were used to establish stocks of each species. Butterflies were caught using butterfly nets within a 2km radius of Gamboa, placed into either glassine envelopes or small mesh pop-up cages and transported back to the Gamboa insectaries, where they were released into stock cages. Progeny of these wild-caught stock butterflies were then individually reared, marked with unique IDs upon eclosion, and used for all experiments. Butterflies were maintained in captivity until their natural deaths.
Reporting on sex	Butterflies were sexed by examining genitalia of freshly-eclosed adults. All cohorts include approximately equal distributions of both sexes. Sex was included as a candidate predictor in all semi-parametric survival models and all functional senescence models, both as a main effect, and as an interaction with other main effects where appropriate (see Supplementary Material, "Statistical analyses"). However, sex was very rarely found to be a significant predictor of any of our variables of interest, and was also not a covariate of interest for us (we were focused on species, age, and diet). Thus, in accordance with our stepwise backward model selection, sex was dropped from models when not found to be a significant predictor in order to facilitate better interpretation of main effects of interest. Because no semi-parametric survival models found sex to be a significant predictor, further analyses with parametric survival models were conducted without sex as a candidate predictor. In the instances in which sex was found to be a significant predictor, it was retained in the models; for example, in the significant interaction between age and sex in body mass analyses (see Results).
Field-collected samples	Experimental cages were outdoors in the STRI Heliconius insectaries, and so temperature was the ambient outdoor temperature (~25'C) and the photoperiod was the Gamboa tropical daylength of approximately 12 hours (~6AM - ~6PM). However, cages were covered with a roof, so butterflies were not directly impacted by rain (although the floors would frequently flood). Butterflies from the insectary cohorts were maintained in 2M x 2M x 2M experimental cages and supplied with sucrose or sucrose/pollen solutions, flowering plants for nectar sources, non-flowering plants for roosting, and host-plants for laying as appropriate. Butterflies from the semi-natural "mark-release-recapture" cohort were kept in a much larger 11M x 11M x 6M cage filled with trees, host-plants, and

	other flowering plants, designed to mimic a natural environment. Butterflies were maintained in experimental cages until the end of their natural lifespans.
Ethics oversight	No ethical approval was required as experiments were conducted on unregulated invertebrate species.

Note that full information on the approval of the study protocol must also be provided in the manuscript.

Dual use research of concern

Policy information about dual use research of concern

Hazards

Could the accidental, deliberate or reckless misuse of agents or technologies generated in the work, or the application of information presented in the manuscript, pose a threat to:

No	Yes	
☒	☐	Public health
☒	☐	National security
☒	☐	Crops and/or livestock
☒	☐	Ecosystems
☒	☐	Any other significant area

Experiments of concern

Does the work involve any of these experiments of concern:

No	Yes	
☒	☐	Demonstrate how to render a vaccine ineffective
☒	☐	Confer resistance to therapeutically useful antibiotics or antiviral agents
☒	☐	Enhance the virulence of a pathogen or render a nonpathogen virulent
☒	☐	Increase transmissibility of a pathogen
☒	☐	Alter the host range of a pathogen
☒	☐	Enable evasion of diagnostic/detection modalities
☒	☐	Enable the weaponization of a biological agent or toxin
☒	☐	Any other potentially harmful combination of experiments and agents

Plants

Seed stocks	Passiflora host-plants for caterpillar rearing and flowering plants used as nectar/pollen sources for adult butterflies were taken from stocks established at the Heliconius insectaries at the Smithsonian Tropical Research Institute in Gamboa, Panama. These stocks were established from local plants collected on an as-needed basis from the field within a 5km radius of Gamboa over the time period in which the insectaries have been operational (10+ years).
Novel plant genotypes	No novel plant genotypes were produced.
Authentication	Identification of field-collected Passiflora plants was conducted by experts with decades of experience working with Passiflora.
