# Supplementary material for: Evolution of increased longevity and slowed ageing in a genus of tropical butterfly
Source: Nat Commun. 2026 Jun 16;17:5077. doi: 10.1038/s41467-026-73635-7 (PMC13272878; doi:10.1038/s41467-026-73635-7)
Supplement: Supplementary file 5 — Transparent Peer Review file [file 41467_2026_73635_MOESM5_ESM.pdf]

nature portfolio

Peer Review File

Evolution of increased longevity and slowed ageing in a genus of tropical butterfly

Corresponding Author: Dr Jessica Foley

This file contains all reviewer reports in order by version, followed by all author rebuttals in order by version.

Version 0:

Reviewer comments:

Reviewer #1

(Remarks to the Author)
Referee report on "Evolution of increased longevity and slowed ageing in a genus of tropical butterfly"

In this study the authors investigated the longevity and senescence rates of adult Neotropical butterflies of the genus Heliconius, which have long been recognized for their variation in lifespan and also their extreme longevity. To their credit, the authors combined several very different sources of data to help build up an understanding of actuarial and physiological aging in this group. Thus, the authors present a literature survey of the maximum reported lifespans of heliconids, an analysis of senescence rates of several species reared in insectary cages as part of a previous "cognitive" experiment, a "semi-natural" mark-release-recapture experiment on 20 species in a large cage, and an experiment comparing the survival and grip strength of Heliconius hecale (a pollen-feeder) and Dryas iulia (which does not feed on pollen) with and without access to pollen. After fitting a range of tailored parametric and non-parametric models to these data, the authors conclude, amongst other things, that pollen-feeding Heliconius species have longer lifespans and reduced ageing parameters relative to non-pollen feeding relatives.

I enjoyed reading this paper and think it provides the most comprehensive overview to date of senescence in this celebrated group. I do however have a number of comments and concerns that I hope will be of use when revising this multi-faceted paper.

1. Pollen-feeding appears to have evolved just once and is common to all Heliconius with the exception of H. aodes (which appears to have subsequently lost pollen feeding, although it was unavailable for this current study). Since Heliconius may be different from other butterflies in more than one respect, it is risky to infer that pollen feeding is the reason for their remarkable longevity. One possibility is that it is down to their unpalatability and/or warning signals (aposematism). It has long been argued that aposematic (unpalatable and warningly coloured) lepidoptera (such as Heliconius) tend to live longer than cryptic species (Blest 1963. Nature, 197, 1183–1186). Neotropical Ithomiine butterflies are highly unpalatable to birds, they are arguably aposematic and are also known for their extended adult lifespans; likewise aposematic migrating monarchs can live 7-9 months. The authors mention Euphaedra medon as a long-lived species but that species may also be aposematic (Molleman et al. 2020 J Anim Ecol 89, 716-729). By contrast Dryas iulia (one of the non-pollen feeders in the data set) is thought to be relatively palatable to birds (e.g. Benson 1971, American Naturalist, 105, 213-226) while Eueides isabela (another of the non-pollen feeders) is also relatively palatable (Chouteau et al. 2019 Animal Behaviour 153, 49-59). Thus, who is to say the pattern is not driven by palatability? Indeed, the conclusion "evolved lifespan extension, slowed actuarial senescence, and delayed physiological senescence in H. hecale, INDEPENDENT of any short-term, plastic benefits of adult pollen intake" rather makes one wonder how important pollen is! Testing the alternate palatability hypothesis may be challenging but since palatability has probably evolved many times in lepidoptera, it may allow a stronger basis for inference. At very least some discussion is needed.

2. It has been postulated that differential survival rates of male and female lepidoptera may be related to their mating system, notably their degree of protandry (see Wiklund et al. 2003 Proc B, 270, 1823–1828). Specifically, it has been argued that butterflies with monandrous mating systems should evolve higher rates of senescence in males, whereas polyandrous species should have similar rates of senescence between males and females. My understanding is that Heliconius are highly protandrous. Would this help explain why there is less evidence of a sex effect in the current analysis?

3. Much is made of the 25-fold variation in lifespan within the Heliconius genus, but very little space has been devoted to help explain it. Lack of line numbers prevent me from giving the precise location of text, but the authors simply state on page 16 “Depending on the timing of this dietary innovation, such mechanisms may have evolved with varying degrees of efficacy in different Heliconius lineages, possibly related to species-specific differences in ecology. This could explain the wide variation in lifespan between Heliconius species…”. This is not at very clear at all! Can one test the timing hypothesis with a time-calibrated phylogeny? What differences in efficiency and ecology are being conjectured here? If there is an idea here, then it should be presented more explicitly.

4. I may have missed it, but it is not clear to me how many replicate cages were used per diet treatment. The Supplementary Methods describe “pollen-fed cages” and “pollen-deprived cages”, but I cannot find specific details. For example, if only one cage per treatment was used, then clearly diet is confounded with cage effects (microclimate, plant distribution etc.). If multiple replicate cages were used (as I suspect) then it would help to report the number of replicate cages, how individuals were distributed across cages, and include cage as a random effect in all fitted models where appropriate etc.

5. The authors use the beta coefficient from a Gompertz to reflect the (scaleless) change in mortality risk with age. This is reasonable but note there has been some debate about this. In particular, Ricklefs & Scheuerlein, 2002 The Journal of Gerontology B 57, B69–B76) argue that an index which combines both the alpha and beta (specifically the root of the product of these parameters) is a better measure of actuarial senescence. On a similar note, when fitting the Gompertz, the estimates of the two parameters will not be independent (the data may be consistent with a higher beta if one assumes a lower alpha etc) and they will also have associated uncertainty. Would it be possible to fit a more general model across all species and compare the fit of models (with AIC or LRT) with species-specific parameters vs fixed parameters?

6. I really like the use of grip strength. Here the grip strength is taken as the maximum of five pulls. Using a maximum is understandable if the aim is to estimate maximal performance, but it can be sensitive to outliers or occasional strong trials. It would help to justify the use of max rather than mean or median, or show that conclusions are robust to using alternative summary metrics. Also, I’m guessing the measure may vary with experimenter. Did the same experimenter do all trials?

7. From the Supplementary file I see that the authors used an “ordbeta” distribution in glmmTMB for their flight analysis. Were there any 0’s or 1’s in the data? If so, how were they handled?

8. Including longevity as a covariate to account for selective disappearance may be appropriate, but the interpretation can be tricky (including longevity may control for among-individual differences that are part of the biological process of interest). Could the authors provide more justification for this, or present results with and without longevity as a covariate so readers can see how much selective disappearance affects age effects.

9. The paper is a very helpful compendium of what we know about aging in Heliconius. I note however that data collection stopped in April 2021. One has to stop somewhere, but I wonder whether it be possible to bring the literature survey more up to date?

10. Reading the abstract, it is not entirely clear what the take-home message should be, but I appreciate that the work is part descriptive and part hypothesis testing. Its only about half-way down the abstract do we see that the work about Heliconius. Did the authors really show “evidence of evolved, heritable mechanisms of slowed ageing in Heliconius” (abstract). What mechanisms were uncovered? How heritable were they (all we know is that there is species variation)? This is pedantic, but do we know that aging has been slowed in heliconids, as opposed to accelerated in the non-heliconids?

Reviewer #2

(Remarks to the Author)
This is an interesting manuscript on lifespan in a particularly long-lived group of butterflies, Heliconius. The authors quantify lifespans of several species using experimental methods and datasets collected by commercial butterfly houses. In addition, the authors experimentally examine the hypothesis of pollen feeding serving as a causal agent that may explain the extended lifespan of some species. The main results include documenting exceptionally long median and maximum lifespans for a group of insects that may have been previously overlooked in the field of ageing research. While pollen feeding was found to be strongly associated with long lifespan at the interspecies level, experimental pollen feeding did not extend lifespan in a non-pollen feeding species, and a naturally pollen feeding species retained long lifespan even in the absence of pollen in the experimental diet. The findings point to the conclusion that long lifespans in Heliconius butterflies have evolved in tandem with long-term investment in reproduction, as Heliconius butterflies have continuous access to nutrient-rich pollen during their adult lives.

As one aim of the manuscript is to present the study system to the wider audience of the ageing research community, I would find it important to point out that what was measured here was adult lifespan of butterflies. For insect scientists this is common knowledge, but this is also an aspect where direct comparisons between taxonomical groups become complicated. While the adult lifespan of Heliconius butterflies may be close to 365 days, there are many Lepidoptera where the full life cycle, including the egg, larval and pupal stages, is two full years or significantly longer. The Arctic woolly bear moth (Gynaephora groenlandica) may live up to 7 years, of which the adult lifespan covers just some weeks. The development of the North American periodical cicadas famously takes even longer, 13 or 17 years. Hibernating adult butterflies, too, can reach lifespans similar to the lifespans of Heliconius butterflies reported here. So, I think the authors should clearly state what makes the lifespans of Heliconius special, and indeed use the term ‘adult lifespan’ where appropriate.

The grip strength assay was novel and really interesting (go Pullinator!), but I wonder what other components are at play, apart from brute muscle strength? Duplouy & Hanski (2013, Biol Lett) performed an experiment measuring grip strength in the Glanville fritillary (Melitaea cinxia) using a flat wooden surface and a strong air current (produced by a hair dryer). They attributed the difference in grip between individuals from island and mainland populations to tarsal claw morphology; more curved claws were correlated with better grip. I would therefore be a bit cautious in interpreting the findings of the grip strength assay, and potential other explanations apart from muscle strength should be discussed.

I must say I'm a bit surprised by the study design of the semi-natural mark-release-recapture experiment: 11 x 11 x 6 metres, trees and bushes, almost a thousand butterflies, and surveyors spent about 15 minutes in the cage once a week? What are the odds of finding a particular butterfly under those conditions? I understand that you will each time find a good number of individuals, but does this kind of sampling give good enough resolution for recording lifespans? The variation in estimated lifespan must have been very large, but I suppose with enough data the median should nevertheless give a decent approximation of true lifespan. However, when performing the study using multiple species, you're assuming they all have the same probability of being found. Is this really a valid assumption? I can think of a number of biases ranging from behavioural differences to differences in butterfly colouration, not forgetting neuropsychological search image biases by the observer that could vary between weeks. The use of the present methodology should be justified, and more details should be provided. How were the individual butterflies marked? How many surveyors were there in the cage? Did they use tools such as binoculars? What were the likely causes of mortality in the cage? (Taking into account that less than a half of the released individuals were resighted.)

Furthermore, longevity data from commercial butterfly houses served as a valuable source of estimated maximum lifespans in a good number of species. I think the idea of using these data is fresh and valuable, but there are some caveats. Do you have any specifications of the conditions in the butterfly houses? We know that ectothermic insects can survive for long periods if kept in cool temperatures. Is there any way to know if there have been unusual conditions during the life of those commercially kept butterflies? In either case, at least some description of the rearing conditions should be provided.

Minor points

Line 16: "hewitsoni longer than any" ---- I think an em dash or some other punctuation is missing here.

Line 100: There might be some newer work on this subject, check Pinheiro et al. 2025: 10.24072/pcjournal.546 - Peer Community Journal, Volume 5 (2025), article no. e53

Line 138: "All butterflies were captive-reared from stock populations at the Smithsonian Tropical Research Institute (STRI) outdoor insectaries in Gamboa, Panama. All Panamanian butterflies were collected under permit number SE/A-14-18 and SE/A-82-19." ---- I don't fully understand this section, were the butterflies of captive origin or field-captured?

Line 154 & 191: The use of the word 'censored' in this context may sound confusing for the non-expert reader.

Reviewer #3

(Remarks to the Author)
Foley et al. investigate the evolution of increased longevity and slowed ageing in Heliconius butterflies. Heliconius butterflies are rather special as they represent a singular evolution of pollen-feeding in butterflies, and as a likely consequence (direct or indirect), show a remarkable increase in longevity. Data presented in this manuscript builds upon several earlier studies showing pollen-feeding and life-history consequences in Heliconius, and presents data across several pollen- and non-pollen feeding species in the Heliconiini tribe using different approaches. Overall, I think the manuscript is extremely well written, by placing a remarkable natural history phenomenon in a solid evolutionary context; arguments are well developed, and statistics seem appropriate. Having said that, I do have some comments.

Singular evolution of pollen-feeding in this clade does pose some problems, but are there other examples that show similar shifts in life history? Authors cite a study on bees, yet some Lepidoptera (Gelechiidae moths), wasps and hoverflies, can feed on pollen. Is there comparable evidence of increased longevity in these groups? On a related note, while studying remarkable clades such as Heliconius – which represent the singular evolution of an unusual trait across butterflies – can provide unique insights, such examples may introduce bias (i.e. ascertainment bias) and may not necessarily reflect general evolutionary mechanisms. The authors could acknowledge this in the manuscript.

It seems like the constant supply of amino acids weakens the trade-off between reproductive effort and longevity (which the authors discuss as well). Moreover, compared to non-pollen feeders, Heliconius seem to adopt a 'slow' life-history strategy (higher investment in offspring, sustained reproductive effort, longer lifespan). What I am getting at is, except from increased longevity/slower ageing, do non-pollen and pollen-feeders differ in other traits, for example, egg size, egg numbers or egg survival? Did the authors quantify these traits? I am trying to understand if the dramatic shift in life-history strategy in pollen-feeding Heliconius (especially longevity) involves correlated changes in different components of reproductive traits.

Similarly, sustained reproductive effort throughout the long lifespan (at least that's what earlier papers showed) is no doubt beneficial, but do certain environmental conditions favour persistence of such a life-history strategy in Heliconius? In other

words, is the pollen they feed on from specific species available throughout the year? Moreover, how seasonal are host plants? I am wondering if the plants are not around, and if butterflies cannot lay eggs, there would be a mismatch between the life-history strategy and ecological conditions.

I do think the lifespan dataset from butterfly farms is great, but I was hoping to find more details on how the butterflies were kept, in what conditions, and especially for Heliconius, what diet were they fed on? I believe these details are generally important, but also because there is a strong emphasis on a single individual surviving for 348 days.

In their mark-recapture experiment, do the authors know if the females were laying eggs or dissected the females after death to check the ovarioles to assess reproductive activity?

How variable were the grip strength measurements, as the authors report that only the maximum value was used in the analyses?

Other comments:
73-76: unclear which are these traits are and which traits are the targets of selection that have having indirect effect on longevity.
84-85: Authors say this, but there is a heavy emphasis on a single individual from a single species.
115-117: Is this true? I thought the studies already showed this (e.g. Dunlap-Pianka et al. 1977)
147-148: Did the artificial protein solution mimic that of the pollen?
469-470: I am unsure what it means by ‘retreat to higher reaches of the lifespan’
482-485: The Authors say this, but there is heavy emphasis on a single individual that survived for 348 days

Version 1:

Reviewer comments:

Reviewer #1

(Remarks to the Author)
I think authors have responded thoroughly and constructively to my earlier comments. My main original concern was that the inference linking slowed senescence and extended lifespan in Heliconius to pollen feeding did not sufficiently consider alternative explanations, notably aposematism/palatability. The authors have now added some discussion of this issue, including relevant theory on extrinsic mortality and senescence, and have moderated some of their language. The authors have also responded well to some methodological and analytical queries. In particular, they have clarified the design of the diet experiment, provided additional justification and sensitivity analyses for the ageing parameters and grip-strength metrics, clarified modelling decisions, and updated the literature survey. These revisions increase confidence in the robustness of the results and improve transparency for readers. One limitation that remains inherent to the design is that the diet manipulation was conducted at the cage level, but I note that the fact that the different species shared a cage and the experiment was conducted in batches over months. Overall, I believe the manuscript is a valuable and comprehensive contribution to the study of lifespan and senescence in insects and will be of broad interest.

Reviewer #2

(Remarks to the Author)
Thank you for your hard work on the revision! To me it looks like all comments and concerns have been answered and the manuscript now reads exceptionally well. Despite my best efforts, I was unable to find even a single typo.

The only comment I have has to do with the added sentence on liness 237-240: The maximum reported lifespan of 348 days for Heliconius hewitsoni also exceeds any butterfly previously recorded in the scientific literature, and is within range of Myscelia cyaniris (380 days), which we now present as the longest-lived butterfly species to date based on data from butterfly exhibitors (Supplementary Data 1).

-- The wording is a bit confusing, as the non-Heliconius Myscelia cyaniris is mentioned here for the first time and its relationship with Heliconius hewitsoni isn’t clear. I’d suggest splitting the sentence in two to make it clearer to understand.

Open Access This Peer Review File is licensed under a Creative Commons Attribution 4.0 International License, which permits use, sharing, adaptation, distribution and reproduction in any medium or format, as long as you give appropriate credit to the original author(s) and the source, provide a link to the Creative Commons license, and indicate if changes were made.
In cases where reviewers are anonymous, credit should be given to 'Anonymous Referee' and the source.
The images or other third party material in this Peer Review File are included in the article's Creative Commons license, unless indicated otherwise in a credit line to the material. If material is not included in the article's Creative Commons license and your intended use is not permitted by statutory regulation or exceeds the permitted use, you will need to obtain permission directly from the copyright holder.
To view a copy of this license, visit https://creativecommons.org/licenses/by/4.0/

Response to Reviewers

Reviewer #1 (Remarks to the Author):

Referee report on “Evolution of increased longevity and slowed ageing in a genus of tropical butterfly”

In this study the authors investigated the longevity and senescence rates of adult Neotropical butterflies of the genus Heliconius, which have long been recognized for their variation in lifespan and also their extreme longevity. To their credit, the authors combined several very different sources of data to help build up an understanding of actuarial and physiological aging in this group. Thus, the authors present a literature survey of the maximum reported lifespans of heliconids, an analysis of senescence rates of several species reared in insectary cages as part of a previous “cognitive” experiment, a “semi-natural” mark-release-recapture experiment on 20 species in a large cage, and an experiment comparing the survival and grip strength of Heliconius hecale (a pollen-feeder) and Dryas iulia (which does not feed on pollen) with and without access to pollen. After fitting a range of tailored parametric and non-parametric models to these data, the authors conclude, amongst other things, that pollen-feeding Heliconius species have longer lifespans and reduced ageing parameters relative to non-pollen feeding relatives.

I enjoyed reading this paper and think it provides the most comprehensive overview to date of senescence in this celebrated group. I do however have a number of comments and concerns that I hope will be of use when revising this multi-faceted paper.

Thank you for your positive comments and considered review.

1. Pollen-feeding appears to have evolved just once and is common to all Heliconius with the exception of H. aodes (which appears to have subsequently lost pollen feeding, although it was unavailable for this current study). Since Heliconius may be different from other butterflies in more than one respect, it is risky to infer that pollen feeding is the reason for their remarkable longevity. One possibility is that it is down to their unpalatability and/or warning signals (aposematism). It has long been argued that aposematic (unpalatable and warningly coloured) lepidoptera (such as Heliconius) tend to live longer than cryptic species (Blest 1963. Nature, 197, 1183–1186). Neotropical Ithomiine butterflies are highly unpalatable to birds, they are arguably aposematic and are also known for their extended adult lifespans; likewise aposematic migrating monarchs can live 7-9 months. The authors mention Euphaedra medon as a long-lived

species but that species may also be aposematic (Molleman et al. 2020 J Anim Ecol 89, 716-729). By contrast Dryas iulia (one of the non-pollen feeders in the data set) is thought to be relatively palatable to birds (e.g. Benson 1971, American Naturalist, 105, 213-226) while Eueides isabela (another of the non-pollen feeders) is also relatively palatable (Chouteau et al. 2019 Animal Behaviour 153, 49-59). Thus, who is to say the pattern is not driven by palatability? Indeed, the conclusion "evolved lifespan extension, slowed actuarial senescence, and delayed physiological senescence in H. hecale, INDEPENDENT of any short-term, plastic benefits of adult pollen intake" rather makes one wonder how important pollen is! Testing the alternate palatability hypothesis may be challenging but since palatability has probably evolved many times in lepidoptera, it may allow a stronger basis for inference. At very least some discussion is needed.

We appreciate this thorough comment and are happy to provide some discussion as requested. Your points are salient, and we had initially also been interested in the palatability argument, considering several lines of evidence that *Heliconius* are less palatable than the Heliconiini outgroups (your references; also [1]). However, we had ultimately rejected this hypothesis, largely because of the mounting arguments from mathematical biologists that there is no solid theoretical basis for Williams' 1957 prediction [2] that reduced extrinsic (i.e. age-independent) mortality should lead to selection for reduced senescence [3-7]. That is, if extrinsic mortality is reduced across all age classes, there should be no effect on senescence. Instead, they argue that the empirical evidence which appears to support this prediction, and has indeed been invoked as such for decades (including, as you mention, Blest, 1963), rely on a misunderstanding of how mortality shapes evolution, and can be explained through alternate interpretations [6]. Mathematical theory instead predicts that age-specific changes in extrinsic mortality can lead to selection on senescence depending on the age classes it impacts, with reduced senescence predicted when extrinsic mortality is reduced for older age classes in comparison to younger age classes, and increased senescence predicted when the reverse is true [3-7].

Therefore the evidence for increased unpalatability of *Heliconius* in comparison with the Heliconiini outgroups could indeed lead to selection for reduced senescence and longer lifespans, but only if this unpalatability increases survival of *Heliconius* adults more than the juvenile stages, and if this discrepancy is more pronounced than that of the outgroup Heliconiini adults v.s. juveniles. *Heliconius* larvae are certainly widely thought to be unpalatable, as they sequester cyanogens from their toxic *Passiflora* host-plants [8]; however, we can find no evidence from predation experiments that they are necessarily less so than *Heliconius* adults. This could be inferred from several studies showing that mature adults of *Heliconius melpomene* have more cyanogenic glucosides than any other developmental stage [9, 10], and that there is some evidence for the accumulation of toxins with age in *Heliconius erato* [11]. However, levels of these compounds do not

necessarily translate directly to unpalatability; for example, despite the evidence for *Heliconius*' increased unpalatability compared to the Heliconiini outgroups outgroups [1, 12, 13], their levels of cyanogenic glucosides are not significantly higher [14]. To support the unpalatability argument, it would be necessary to show not only that extrinsic mortality is specifically reduced in *Heliconius* adults relative to juveniles, but also that this reduction is disproportionately greater than in outgroup species. At present, comparative data on predation levels across the Heliconiini tribe is lacking for juveniles.

Considering these caveats, we felt that the robust evidence for an extended reproductive lifespan in pollen-feeding *Heliconius* as compared to non-pollen-feeding Heliconiini [15-18] is more consistent with the available data than the possibility of an increase in age-specific unpalatability. This interpretation is further supported by a phylogenetically broad comparative analysis of butterfly lifespans, which found that aposematism is indeed associated with increased lifespan, but is less important than adult feeding strategy (specifically pollen and fruit feeding versus nectar feeding), which has larger effect sizes and is retained in most 95% confidence sets of models, whereas aposematism is retained in about half [19]. There is also the suggestion that pollen-fed *Heliconius* are more toxic than *Heliconius* deprived of pollen [20], although evidence for this is conflicting [21]. If so, this could make the case for the effects of pollen-feeding on lifespan being mediated through an age-specific increase in chemical defences (in the adult stage); although again, this does not necessarily translate to increased unpalatability, and conflicting evidence prohibits us from drawing this conclusion.

However, we do appreciate your point, and agree that other readers may have the same questions. As requested, we have therefore added a discussion of the unpalatability argument, and the theory behind extrinsic mortality and senescence, to the Discussion section (lines 3591-375). We also agree with your point, which aligns with the opinion of Reviewer #3, that with an *n* of 1 for the evolution of pollen-feeding, we cannot definitively confirm that the changes in *Heliconius* are the direct result of a transition to pollen-feeding – although we do think that the pollen-feeders' extended reproductive lifespan, which remains a theoretically sound basis for selection for longer lifespans, makes ours a compelling argument. Our experimental data also clearly shows that pollen feeding does impact survival and condition, providing evidence of a biological link. We have edited the Discussion accordingly.

2. It has been postulated that differential survival rates of male and female lepidoptera may be related to their mating system, notably their degree of protandry (see Wiklund et al. 2003 Proc B, 270, 1823–1828). Specifically, it has been argued that butterflies with monandrous mating systems should evolve higher rates of senescence in males,

whereas polyandrous species should have similar rates of senescence between males and females. My understanding is that Heliconius are highly protandrous. Would this help explain why there is less evidence of a sex effect in the current analysis?

Thank you for this interesting reference. Because sex was not the primary interest of this study, we had not initially planned to discuss our lack of evidence for sex effects. However, in light of this comment, we have updated the Discussion (lines 329-336) to give some brief reference to it, including this potential explanation you have helpfully provided.

3. Much is made of the 25-fold variation in lifespan within the Heliconius genus, but very little space has been devoted to help explain it. Lack of line numbers prevent me from giving the precise location of text, but the authors simply state on page 16 "Depending on the timing of this dietary innovation, such mechanisms may have evolved with varying degrees of efficacy in different Heliconius lineages, possibly related to species-specific differences in ecology. This could explain the wide variation in lifespan between Heliconius species…". This is not at very clear at all! Can one test the timing hypothesis with a time-calibrated phylogeny? What differences in efficiency and ecology are being conjectured here? If there is an idea here, then it should be presented more explicitly.

We appreciate that this wording is vague, but this reflects the lack of available data to test specific hypotheses. We think there are many interesting questions that could be asked and answered with finer-scale ecological detail about longer- and shorter-lived *Heliconius* species. For example, different species occupy different habitats, with some for example found at higher altitudes, which may lead to differing lifespans in these ectothermic butterflies (as pointed out by Reviewer #2). However, these altitudinal differences often vary within species, and are also often confounded by other potentially important differences, e.g. whether the species exhibits pupal mating [22], making it difficult to draw conclusions here. Another possible effect is from larval gregariousness, which might be expected to lead to life-history differences [23]; notably, larval gregariousness is exhibited by our longest-lived species, *Heliconius hewitsoni*, but also by one of the shortest-lived species, *Heliconius doris* (with short lifespans reported across 2 separate studies, see Table 1 and Table 3). The inconsistent directionality once again prevents us from further meaningful speculation. We suggest therefore that testing these more fine-scale hypotheses would require substantial, specifically designed data collection and field experiments, and as such we are unable to include them here.

We believe that our paper lays the groundwork for interesting hypothesis testing comparing these lifespans with finer-scale ecological data, which we hope may lead to

future projects for other biologists. However, we feel that with our current data, such conjecture is beyond the scope of this manuscript. We have updated the abstract to remove reference to this 25-fold variation (which is between Heliconiini; the variation in *Heliconius* is 5-fold, see Table 1) to reduce emphasis and avoid expectation that we plan to discuss it further. We have also edited the wording in the Discussion to remove reference to the "timing hypothesis", which was intended to loosely refer to the lack of agreement among *Heliconius* biologists over which key innovation (with pollen-feeding being one candidate) drove the *Heliconius* adaptive radiation [24-27].

4. I may have missed it, but it is not clear to me how many replicate cages were used per diet treatment. The Supplementary Methods describe "pollen-fed cages" and "pollen-deprived cages", but I cannot find specific details. For example, if only one cage per treatment was used, then clearly diet is confounded with cage effects (microclimate, plant distribution etc.). If multiple replicate cages were used (as I suspect) then it would help to report the number of replicate cages, how individuals were distributed across cages, and include cage as a random effect in all fitted models where appropriate etc.

We apologise for the lack of clarity, which we have now rectified in the Methods and the Supplementary Methods. Due to practical and space constraints in the insectaries, which are shared with other lab groups, and the large size of the cages we used ($12m^3$), these experiments were performed in a single cage per treatment. We standardised conditions between the two cages, ensuring similar plant distributions/quality, the same number of food resources, and the same regular checks for predators. The cages were also adjacent to each other, making it unlikely that there would be meaningful microclimatic differences. We favored a large, open cage over multiple small cages, as Heliconiini do not behave naturally in enclosed spaces, which leads to higher death rates due to wing damage caused by flying against the cage walls.

We of course understand the limitations of this approach. However, we would like to highlight two points that are salient to the interpretation of the data: first, each cage always contained butterflies of both species, in equal ratios, and so if there was a clear cage effect (e.g. some microclimatic difference meaning survival was lower in the pollen-deprived cage) it would impact survival in *D. iulia* as well as *H. hecale.* Because the effects on survival are only seen in *H. hecale*, this would have to imply the presence of a species-specific cage effect, and considering the broad physiological similarities between these two species, including larval hostplants and adult habitats, we can think of no explanatory factor other than the dietary difference. Second, it is important to note these experiments were not done in a single batch, but conducted over an 8-month period, with butterflies continuously added to the cages throughout (with the first butterfly introduced to these cages on 12/3/22, and the last dying on 15/11/22), with

data analyzed at an individual level. This again makes the suggestion of a non-diet-related cage effect seem improbable as such an effect would have had to be consistent across the whole experiment – which, for example, spanned climatically variable wet and dry seasons, dwarfing any minute differences between neighboring cages. As such, within the practical constraints of the facilities, we are confident that our approach, data and inferences are robust.

5. The authors use the beta coefficient from a Gompertz to reflect the (scaleless) change in mortality risk with age. This is reasonable but note there has been some debate about this. In particular, Ricklefs & Scheuerlein, 2002 The Journal of Gerontology B 57, B69–B76) argue that an index which combines both the alpha and beta (specifically the root of the product of these parameters) is a better measure of actuarial senescence. On a similar note, when fitting the Gompertz, the estimates of the two parameters will not be independent (the data may be consistent with a higher beta if one assumes a lower alpha etc) and they will also have associated uncertainty. Would it be possible to fit a more general model across all species and compare the fit of models (with AIC or LRT) with species-specific parameters vs fixed parameters?

We prefer to use the beta coefficient because it appears to be much more widely used in the field. However, we have now also calculated this combined index of actuarial senescence for all 3 cohorts for which we derived Gompertz parameters, and presented them side-by-side in Supplementary Note 9 for interested readers to review; we have updated the Methods (line 593) to refer to this. Consistent with our main analyses, the rate of ageing as measured by this combined index is still almost exclusively lower in *Heliconius* as compared with the Heliconiini outgroups.

With regards to your second point, this is indeed what we did, at least for the multi-species cognitive cohort and the pollen-manipulation cohort. For the semi-natural mark-release-recapture cohort it was not possible to fit general models across all species in *BaSTA* because of a) the different recapture probability for each species and b) the different census lengths for each species (see our response to Reviewer #2, and the updated details on *BaSTA* model fitting in the Methods and Supplementary Methods for more information). For the other two groups, we fit models that allowed either the alpha or beta parameter, or both, or neither, to vary by species (for the multi-species cognitive cohort), or species / diet (for the pollen-manipulation cohort), and compared the fixed models to models with species-specific parameters using AIC as you suggest. We have updated the text in the Methods section to clarify this. Please see Supplementary Notes 2 and 4 for the tables with these model comparisons.

6. I really like the use of grip strength. Here the grip strength is taken as the maximum of five pulls. Using a maximum is understandable if the aim is to estimate maximal performance, but it can be sensitive to outliers or occasional strong trials. It would help to justify the use of max rather than mean or median, or show that conclusions are robust to using alternative summary metrics. Also, I'm guessing the measure may vary with experimenter. Did the same experimenter do all trials?

Thank you for your enthusiasm for these experiments. We were indeed aiming to estimate maximal performance, and were following the methodological approach of another paper [28], which also selected the maximum reading out of 5 trials. We have updated the Methods (lines 568-572) to justify this decision. We also note that occasionally individuals did not properly "grip" the perch in the trials, leading to unrealistically low readings, but we were unconcerned about this when conducting the assays because we had decided *a priori* to use the maximum out of 5 readings, and so these lower readings would not have affected downstream analyses. It is therefore likely that the mean or the median would be an underestimation of the true capability of these butterflies. However, we can understand your concerns re: the sensitivity of the maximum to outliers, and so we went back to our data to further justify our use of this metric. We first found that the maximum reading for grip strength was highly correlated with the mean reading across all 5 trials for each biweekly assay, both for *H. hecale* (r = 0.92, p < 0.001) and for *D. iulia* (r = 0.92, p < 0.001), as shown in Figure 1. We have now included these details in Supplementary Note 6 for any interested readers. We also updated the models to remove any observations with studentised residuals > 3, which is a common technique for identifying potential outliers. These models returned the same main findings, with reduced grip strength in pollen-deprived *H. hecale* ($F_{1, 73.52}$ = 4.60, p = 0.035), but no difference between diets in *D. iulia* ($F_{1, 81.76}$ = 3.24, p = 0.076), and a decline in grip strength with age in *D. iulia* ($F_{1, 99.48}$ = 11.92, p = 0.001) but not *H. hecale* ($F_{1, 225.11}$ = 1.65, p = 0.201).

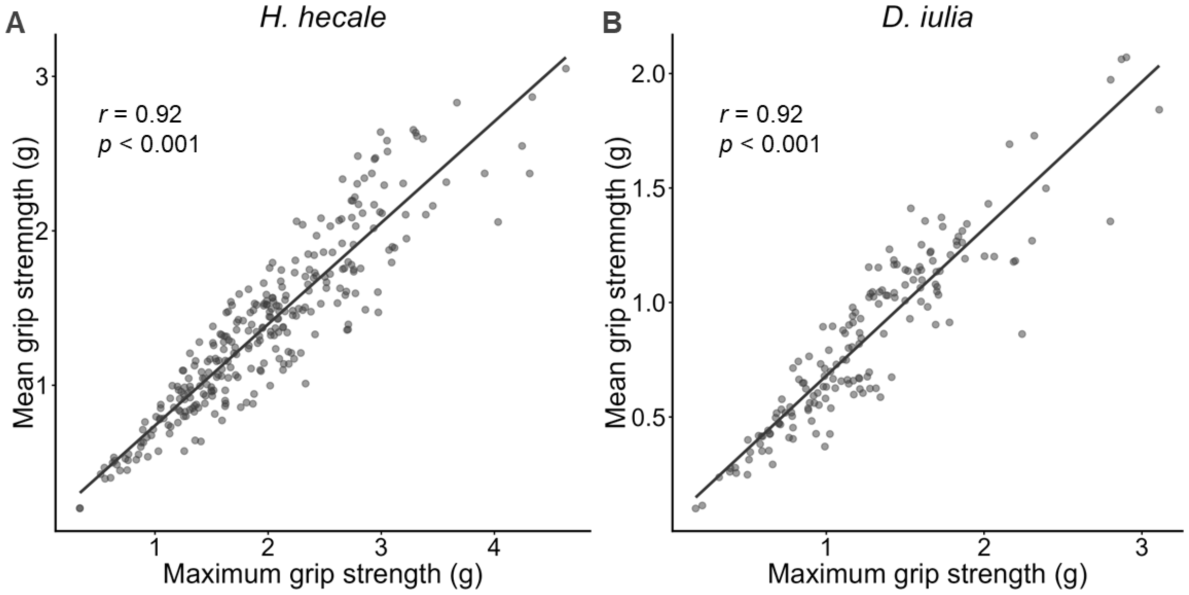

Figure 1

With respect to your second question, lead author Jessica Foley conducted or supervised almost all of the grip strength trials across the 10-month field season. There were rare occasions when they were conducted by some of the other co-authors after training by JF, which occurred randomly across the time period, individuals, species and treatment groups (i.e. without any consistent distribution that could bias the results).

7. From the Supplementary file I see that the authors used an "ordbeta" distribution in glmmTMB for their flight analysis. Were there any 0's or 1's in the data? If so, how were they handled?

The ordered beta regression model fit using "ordbeta" in glmmTMB is a relatively recently developed model [29] which combines both beta regression and ordered logit, meaning it can be fit to both the continuous middle part of proportion data as well as the 0s and 1s at the bounds of the scale. Therefore it can handle 0s and 1s in the data, of which there were many; this was our main reason for selecting this model.

8. Including longevity as a covariate to account for selective disappearance may be appropriate, but the interpretation can be tricky (including longevity may control for among-individual differences that are part of the biological process of interest). Could

the authors provide more justification for this, or present results with and without longevity as a covariate so readers can see how much selective disappearance affects age effects.

We had included longevity as a covariate because we were primarily interested in within-individual differences related to the process of senescence. We have now provided more justification for this in the Methods (lines 634-639). However, we have also run the models for body weight, grip strength, and flight behaviour without longevity included as a fixed effect, and these resulted in qualitatively similar findings. We have now presented these results in Supplementary Note 10 for any interested readers.

9. The paper is a very helpful compendium of what we know about aging in Heliconius. I note however that data collection stopped in April 2021. One has to stop somewhere, but I wonder whether it be possible to bring the literature survey more up to date?

We have since repeated our literature search and found a single new maximum lifespan record for *Heliconius erato* of 107 days [11]. This does not eclipse our previous record for *H. erato*, but we have added it to our larger table of all available maximum lifespan sources in Supplementary Data 2. We also requested some data from a *Heliconius himera* mark-release-recapture preprint from our collaborators [30], who helpfully provided us with a new maximum observed lifespan of 94 days for this species (now added to Supplementary Data 2). However, we then realised that our butterfly house dataset from Mr. Kelson did in fact also have data for *H. himera*, with a maximum recorded lifespan of 155 days. It had initially been misclassified as an *erato* subspecies in this dataset, and so we had missed it when originally collating data on maximum observed lifespan. This additional species maximum has now been added to Table 1 as well as the phylogeny in Figure 1, and we have recalculated statistics related to maximum lifespans in the first paragraph of the results, as well as Supplementary Note 1. However, addition of this species did not meaningfully change our findings; pollen-feeding *Heliconius* species still show longer maximum reported lifespans (mean: 177.36 days) than non-pollen-feeding Heliconiini outgroups (mean: 57.67 days) ($t_{21.88} = 5.81$, $p < 0.001$). Therefore we did not make any changes to our Discussion related to this update.

10. Reading the abstract, it is not entirely clear what the take-home message should be, but I appreciate that the work is part descriptive and part hypothesis testing. Its only about half-way down the abstract do we see that the work about Heliconius. Did the authors really show "evidence of evolved, heritable mechanisms of slowed ageing in Heliconius" (abstract). What mechanisms were uncovered? How heritable were they (all

we know is that there is species variation)? This is pedantic, but do we know that aging has been slowed in heliconids, as opposed to accelerated in the non-heliconids?

We have shortened the abstract so that reference to *Heliconius* appears earlier. We also agree that use of the word “mechanisms” is misleading; we believe our findings are evidence that such mechanisms exist, but we have not identified these in this paper, and so we have removed this word from the abstract. We also would like to argue that species variation implies heritable, genetic differences, especially as we show evidence for effects that are not environment-dependent. As noted in the abstract, the take-home message is that while dietary pollen provides direct benefits to *Heliconius* lifespan, their lengthened lifespan even under pollen-deprivation is evidence for an evolved lifespan extension and slowed ageing in this genus. Consistent evidence across the Heliconiini tribe points to conserved pro-longevity mechanisms that now present a promising target for mechanistic analysis, highlighting *Heliconius* as a powerful system for the study of extended longevity.

With respect to the point about ageing being slowed in heliconids as opposed to accelerated in the non-heliconids, it is clear from the Heliconiini phylogeny that an explanation invoking slowed aging in *Heliconius* is far better supported by the data. *Heliconius* form a single monophyletic clade, whereas groups of the non-heliconids are non-monophyletic. A more distant phylogenetic view also supports this; the closely-related *Speyeria mormonia*, which is also in the larger Heliconiinae subfamily (see [27], Fig. 1 for phylogenetic position), has an adult lifespan of approximately 15 days [31]. Based on this evidence, slowed ageing in *Heliconius* seems by far the most likely explanation.

Reviewer #2 (Remarks to the Author):

This is an interesting manuscript on lifespan in a particularly long-lived group of butterflies, Heliconius. The authors quantify lifespans of several species using experimental methods and datasets collected by commercial butterfly houses. In addition, the authors experimentally examine the hypothesis of pollen feeding serving as a causal agent that may explain the extended lifespan of some species. The main results include documenting exceptionally long median and maximum lifespans for a group of insects that may have been previously overlooked in the field of ageing research. While pollen feeding was found to be strongly associated with long lifespan at the interspecies level, experimental pollen feeding did not extend lifespan in a non-pollen feeding species, and a naturally pollen feeding species retained long lifespan even in the absence of pollen in the experimental diet. The findings point to the conclusion that long lifespans in Heliconius butterflies have evolved in tandem with long-term investment in reproduction, as Heliconius butterflies have continuous access to nutrient-rich pollen during their adult lives.

Thank you for your positive comments and considered review.

As one aim of the manuscript is to present the study system to the wider audience of the ageing research community, I would find it important to point out that what was measured here was adult lifespan of butterflies. For insect scientists this is common knowledge, but this is also an aspect where direct comparisons between taxonomical groups become complicated. While the adult lifespan of Heliconius butterflies may be close to 365 days, there are many Lepidoptera where the full life cycle, including the egg, larval and pupal stages, is two full years or significantly longer. The Arctic woolly bear moth (Gynaephora groenlandica) may live up to 7 years, of which the adult lifespan covers just some weeks. The development of the North American periodical cicadas famously takes even longer, 13 or 17 years. Hibernating adult butterflies, too, can reach lifespans similar to the lifespans of Heliconius butterflies reported here. So, I think the authors should clearly state what makes the lifespans of Heliconius special, and indeed use the term 'adult lifespan' where appropriate.

Thank you for pointing this out. We have updated the Methods section (lines 576-581) to clarify that we are specifically measuring adult lifespan, disregarding the larval and pupal stages. We have also pointed out that these are largely uniform across all Heliconiini species, to emphasise that *Heliconius*' longevity comes from an extension of adult lifespan (which is also referred to at points in the Discussion referring to Heliconiini development and common juvenile life histories). We have also further emphasized this

and referenced other long lepidopteran lifespans in the Introduction (lines 54-60) to highlight what sets *Heliconius* apart from these other examples.

The grip strength assay was novel and really interesting (go Pullinator!), but I wonder what other components are at play, apart from brute muscle strength? Duplouy & Hanski (2013, Biol Lett) performed an experiment measuring grip strength in the Glanville fritillary (Melitaea cinxia) using a flat wooden surface and a strong air current (produced by a hair dryer). They attributed the difference in grip between individuals from island and mainland populations to tarsal claw morphology; more curved claws were correlated with better grip. I would therefore be a bit cautious in interpreting the findings of the grip strength assay, and potential other explanations apart from muscle strength should be discussed.

Thank you for your enthusiasm for the Pullinator, and for the recommendation of another paper with unconventional methodology for measuring grip strength. We agree with your point about differences in claw morphology potentially influencing grip strength, which is why we did not interpret inter-species differences between *H. hecale* and *D. iulia*. Instead, we were primarily focused on either intra-species differences, i.e. between adult diet treatments, or on intra-individual differences with increasing age (controlling for repeated measures using ID as a random effect). As tarsal claw morphology is determined at pupation, we do not expect that either differing adult diets or increasing age would affect claw morphology and lead to biases in our comparisons of interest.

I must say I'm a bit surprised by the study design of the semi-natural mark-release-recapture experiment: 11 x 11 x 6 metres, trees and bushes, almost a thousand butterflies, and surveyors spent about 15 minutes in the cage once a week? What are the odds of finding a particular butterfly under those conditions? I understand that you will each time find a good number of individuals, but does this kind of sampling give good enough resolution for recording lifespans? The variation in estimated lifespan must have been very large, but I suppose with enough data the median should nevertheless give a decent approximation of true lifespan. However, when performing the study using multiple species, you're assuming they all have the same probability of being found. Is this really a valid assumption? I can think of a number of biases ranging from behavioural differences to differences in butterfly colouration, not forgetting neuropsychological search image biases by the observer that could vary between weeks. The use of the present methodology should be justified, and more details should be provided. How were the individual butterflies marked? How many surveyors were

there in the cage? Did they use tools such as binoculars? What were the likely causes of mortality in the cage? (Taking into account that less than a half of the released individuals were resighted.)

We have included further details in the manuscript (lines 501-532) on how butterflies were marked (with a marker of contrasting colour), possible causes of mortality (predation by spiders and ants), and how many surveyors were in the cage (in most cases, just one at a time). The surveyors did not use tools such as binoculars. We had initially quoted weekly visits in the Methods, but we have since double-checked the data and in actual fact completed 102 patrols over 42-week period, and so have updated the manuscript to reference a twice-weekly cage-check. Nonetheless, we of course agree that increased sampling rates would have been beneficial, but this experiment occurred concurrently with our main diet-treatment experiment, and rearing and experimental time costs are substantial in this system, preventing more intensive sampling. Despite this, we point to the significant correlation between median lifespan estimates from this cohort and maximum reported lifespan as shown in Table 1 (r = 0.62; t_{15} = 3.07, p = 0.008; see Supplementary Note 3 for a plot of this correlation) as a compelling detail supporting the validity of this experiment.

We also agree that it is highly unlikely that each species would have the same probability of being found. However, this is accounted for in our statistical analysis. The *BaSTA* package estimates recapture probability for each cohort based on the data (i.e. how frequently individuals were resighted), and uses this to estimate age-specific survival for that cohort under a Bayesian framework [32]. Primarily for this reason, but also because census lengths differed between species (e.g. while all data collection ceased on 9/1/23, we placed our first *H. erato* into the cage on 2/3/22, but not our first *Agraulis vanillae* until 3/6/22), we constructed a new *BaSTA* model for each species. This used the species-specific sighting data to estimate a new recapture probability for each species, and used that figure to construct the predicted survival trajectory for each species separately. Therefore differences in recapture probability between species should not be a source of bias. We have updated the Methods (lines 609-613) to refer to this, and have also included additional details on *BaSTA* model fitting in the Supplementary Methods.

Furthermore, longevity data from commercial butterfly houses served as a valuable source of estimated maximum lifespans in a good number of species. I think the idea of using these data is fresh and valuable, but there are some caveats. Do you have any specifications of the conditions in the butterfly houses? We know that ectothermic insects can survive for long periods if kept in cool temperatures. Is there any way to

know if there have been unusual conditions during the life of those commercially kept butterflies? In either case, at least some description of the rearing conditions should be provided.

We appreciate your enthusiasm about our use of these data. While we collected data from several butterfly exhibitors for this section, all of the highest butterfly house records for maximum lifespan for each species (Table 1) were obtained from the Butterfly Habitat exhibit at Six Flags Discovery Kingdom in Vallejo, California, where coauthor Richard Kelson is the lead entomologist. We provided some details on data collection for this dataset in the Supplementary Material, but we recognize that we neglected to include information on conditions in the exhibit, and so we have now rectified this in Supplementary Note 5. In brief, the dimensions of the exhibit are 30m (L) x 15m (W) x 6m (H), many floral nectar/pollen resources are provided (including Heliconiini favourites such as *Lantana* and *Stachytarpheta*), and these are supplemented with artificial feeders containing a 20% v/v honey solution.

In response to your question about temperatures, they range between 16-32°C throughout the year. We appreciate that seasonal cooler periods may affect lifespan, but many of the longer-lived species listed in Table 1 (which presumably are the ones in question re: potentially unnaturally extended lifespan) have maximum lifespans of over 6 months, and so it seems unlikely that these figures were artefacts of a transiently cool environment. These temperatures are also often well within the range of many species' natural habitat (*Heliconius hewitsoni*, for example, has an altitudinal range in Costa Rica of sea-level to 3500m [33]).

We believe that while each of the datasets may have their limitations, a major strength of our study is the triangulation of several different sources of data allows for complementary supplementation of these pitfalls, and ultimately supports our overall conclusions. The available data from the literature and commercial butterfly houses provided the justification for the study, but study conditions were highly variable and these data only offered insight into one metric of ageing (maximum lifespan). Our repurposed cognitive cohort allowed for individual level-tracking of survival across several Heliconiini species, but may have introduced biases due to the stressful learning and memory assays, and provided no information about the importance of dietary pollen. Our targeted pollen-manipulation cohort provided precise, carefully-collected survival data allowing us to interrogate the role of pollen, but was necessarily limited to two species due to the intensity of workload for that experiment. The mark-release-recapture study allowed us to collect at least some survival data from a much wider breadth of species across the Heliconiini phylogeny to generalise some of the smaller-scale findings to the larger tribe. Again, we accept that none of the datasets is perfect, but we believe that this triangulation offers a compelling case for our overall conclusions.

Minor points

Line 16: "hewitsoni longer than any" ---- I think an em dash or some other punctuation is missing here.

We have removed this clause from the abstract.

Line 100: There might be some newer work on this subject, check Pinheiro et al. 2025: 10.24072/pcjournal.546 - Peer Community Journal, Volume 5 (2025), article no. e53

Thank you for pointing this out. This paper does indeed support the evidence for reproductive senescence in pollen-deprived *Heliconius*, and so we have edited the citation in line 105 to reflect this.

Line 138: "All butterflies were captive-reared from stock populations at the Smithsonian Tropical Research Institute (STRI) outdoor insectaries in Gamboa, Panama. All Panamanian butterflies were collected under permit number SE/A-14-18 and SE/A-82-19." ---- I don't fully understand this section, were the butterflies of captive origin or field-captured?

All experimental butterflies were captive-reared from stock populations established at STRI, and these stocks were established using field-captured butterflies. These stocks were established either by our lab group during our field seasons, or by STRI scientists maintaining the insectaries in the years preceding our arrival. We have edited the Methods section to clarify this, adding an additional few lines (475-476; 482-487; 507-509; 537-540) for each sub-section, as stock provenance differs for each experiment.

Line 154 & 191: The use of the word 'censored' in this context may sound confusing for the non-expert reader.

We have edited these lines (495 & 552) to instead read "noted for censorship in survival analysis", so that it is clear to any non-expert reader that this is a characteristic of this statistical methodology.

Reviewer #3 (Remarks to the Author):

Foley et al. investigate the evolution of increased longevity and slowed ageing in Heliconius butterflies. Heliconius butterflies are rather special as they represent a singular evolution of pollen-feeding in butterflies, and as a likely consequence (direct or indirect), show a remarkable increase in longevity. Data presented in this manuscript builds upon several earlier studies showing pollen-feeding and life-history consequences in Heliconius, and presents data across several pollen- and non-pollen feeding species in the Heliconiini tribe using different approaches. Overall, I think the manuscript is extremely well written, by placing a remarkable natural history phenomenon in a solid evolutionary context; arguments are well developed, and statistics seem appropriate. Having said that, I do have some comments.

Thank you for your encouraging comments and considered review.

Singular evolution of pollen-feeding in this clade does pose some problems, but are there other examples that show similar shifts in life history? Authors cite a study on bees, yet some Lepidoptera (Gelechiidae moths), wasps and hoverflies, can feed on pollen. Is there comparable evidence of increased longevity in these groups? On a related note, while studying remarkable clades such as Heliconius – which represent the singular evolution of an unusual trait across butterflies – can provide unique insights, such examples may introduce bias (i.e. ascertainment bias) and may not necessarily reflect general evolutionary mechanisms. The authors could acknowledge this in the manuscript.

Thank you for pointing this out. As far as we were aware at the time of writing the first draft, there was a relative paucity of data on life history traits (in particular, longevity) for other insects which have evolved pollen-feeding. In response to this comment, we have completed an exhaustive literature search and arrived at much the same conclusion. Broadly speaking, while there is occasional experimental evidence for slight increases in longevity when providing dietary pollen to insects known to have evolved this behaviour (e.g. in the hoverfly *Episyrphus balteatus* [34, 35], or in the parasitoid wasp *Scambus buolianae* [36], see below for further detail), we cannot find any evidence for an evolved lifespan extension as a result of this enhanced diet (i.e., comparative lifespan data between closely-related pollen-feeding and non-pollen-feeding taxa). This is despite the apparent benefits of pollen to egg production in several insects, in particular in hoverflies, where it is essential for egg maturation throughout the adult lifespan [37, 38]. The hoverfly case offers something of a parallel with what we see in *Heliconius*, and it may therefore seem surprising that we do not find benefits to lifespan in these insects. However, again, the paucity of available lifespan data for these species, and in particular the lack of comparative lifespan data for closely-related pollen-feeders and non-pollen-feeders, makes it impossible to reasonably draw any inferences. We note, however, that a dietary resource change does not in itself provide a selection pressure to change longevity, but may provide a context for those resources to be used in that

way. The listed species have different life histories and ecologies, and may extract different resources from pollen itself, or allocate these resources to different tissues, and as such we feel it is simplistic to expect a similar response in each group. Regardless, evidently what is first needed is a comparative analysis for each clade in which pollen-feeding arose, comparing lifespan of pollen-feeders and non-pollen feeders to assess any differences in longevity, which we note is not a trivial task. We provide a more detailed review of all literature surveyed with respect to pollen-feeding and lifespan in other insects below.

We found little data on lifespan in pollen-feeding moths. Gelechiidae species which feed on pollen as adults (*Deltophora* spp.) are reportedly not observed in the adult stages outside of the 3-month flowering season of their *Phyllanthus* pollen sources, and can enter pupal diapause in the interim [39]. This suggests that adult lifespan in these species is bounded by the seasonality of their hosts-plants, in agreement with your comment below. The moth *Batrachedra nuciferae*, which feeds on palm flower pollen as a caterpillar, has been reported to have a maximum adult longevity of 18 days [40], which could hardly be assumed to be evidence of an extended lifespan even if there were available lifespan data for its non-pollen-feeding close relatives (which we could not find). However, the restriction of this behaviour to the larval phase, where pollen appears to be this species' primary food source [41], suggests a possible substitution rather than addition of nitrogenous material (as in *Heliconius*), and therefore would not be expected to lead to benefits to lifespan.

In hoverflies, which are known to feed on pollen as adults, pollen-derived nitrogenous resources are essential for maturation of the reproductive organs in males, and for egg maturation throughout the adult lifespan in females [37, 38] (much like in *Heliconius*). There is certainly variation between species in the degree of pollen consumption, which is much smaller for some hoverflies [42], but experimentally, quantity of pollen consumed has not been found to correlate with fitness in female *E. balteatus* [38]. Moving to a more binary (pollen-fed/pollen-deprived) framework, further experimental studies have corroborated the finding that provision of pollen is essential for egg production in *E. balteatus* females [34], and also leads to slight increases in longevity [34, 35]. However, effect sizes are very small, and similar increases have separately been observed with provision of just glucose or honey to otherwise pollen-deprived *E. balteatus* (males and females) [43].

Some species of vespid wasps, most notably in the Masarinae subfamily, actively feed on pollen [44], but again there is a paucity of lifespan data for these species, with the best available estimates suggesting adult lifespans of up to several months at most [45]. In contrast, lifespans of up to 506 days have been reported for some other vespids not known to feed on pollen [46]. However, this is confounded with sociality, which is known to correlate with lifespan across animal species [23] and is in particular associated with long life in eusocial insect reproductives [47]. We found no studies experimentally manipulating pollen intake in this system. There is also evidence for pollen-feeding in some parasitoid wasps, although this is often thought to be an inadvertent consequence of feeding on nectar and honeydew [48]. Experimental manipulations have found slight increases in

lifespan and egg production in pollen-fed as compared with pollen-deprived *Scambus buolianae* females, but once again, effect sizes are small, and there is no effect of the pollen diet on lifespan in males [36]. More recent experiments on *Torymus sinesis* also reported no differences in egg production (for females) nor lifespan (in either sex) between pollen-fed and pollen-deprived diets [49].

We have updated the Discussion to include reference to these other groups which have also evolved pollen-feeding (lines 259-264; 344-350). We also acknowledge your point about the difficulty in drawing conclusions about general mechanisms when there has been a singular evolution of pollen-feeding at the base of *Heliconius*; Reviewer #1 had similar thoughts, and we have updated the Discussion accordingly to address this (lines 344-350; 359-375). However, we would like to emphasise that we are not advocating for pollen-feeding as an evolutionarily-conserved mechanism for longevity increases in insects. Rather, we are specifically interested in the longevity increase in *Heliconius*. Based on our results, and the work that our findings build upon, we believe that the evolution of pollen-feeding would have provided the conditions for lifespan extension in *Heliconius* specifically, through the lengthened reproductive lifespan it directly facilitates, together with their continuous reproduction with overlapping generations in a largely aseasonal environment characterized by relatively constant pollen sources and host plants. We are not arguing for a general role of pollen-feeding in insect longevity, unless the system of interest also meets these conditions, which as far as we are aware, the above examples do not – although we would of course welcome further research on these systems.

It seems like the constant supply of amino acids weakens the trade-off between reproductive effort and longevity (which the authors discuss as well). Moreover, compared to non-pollen feeders, Heliconius seem to adopt a 'slow' life-history strategy (higher investment in offspring, sustained reproductive effort, longer lifespan). What I am getting at is, except from increased longevity/slower ageing, do non-pollen and pollen-feeders differ in other traits, for example, egg size, egg numbers or egg survival? Did the authors quantify these traits? I am trying to understand if the dramatic shift in life-history strategy in pollen-feeding Heliconius (especially longevity) involves correlated changes in different components of reproductive traits.

In general terms, life history in the larval and pupal stages is highly similar across Heliconiini [50]. However, there is some evidence of diet effects on oviposition rates. Dunlap-Pianka *et al*. showed lower daily egg production in pollen-fed *Heliconius charithonia* in comparison to the non-pollen-feeding outgroup *Dryas iulia*. They also showed that egg production is maintained throughout the adult lifespan of pollen-fed *H. charithonia*, whereas it tapers off after about a month in *D. iulia*, resulting in higher

lifetime egg output in *H. charithonia*. They then showed that reproductive physiology in pollen-deprived *H. charithonia* recapitulates that of the non-pollen-feeding *D. iulia*, with butterflies showing a cessation of egg-laying after around 20 days. They support these findings with results from ovarian dissections showing clear signs of ovarian senescence in ageing *D. iulia* and *Agraulis vanillae* (another non-pollen-feeding outgroup), as well as in pollen-deprived *H. charithonia*, but not in pollen-fed *H. charithonia*, nor in *Heliconius cydno*. They did not quantify egg survival, nor egg size, which is known to vary across Heliconiini but does not show clear patterns related to pollen-feeding [51]. Their findings on daily oviposition rate have also been much more recently corroborated in *H. erato*, where pollen-deprived females were once again found to lay fewer eggs with age in comparison to pollen-fed females, but only after 45 days of pollen deprivation, not at 14 days, suggesting the effect accumulates as larval resources are depleted [20].

We also attempted to corroborate these impacts of species, diet, and age on oviposition in our own study on *H. hecale* and *D. iulia*. However, we were also collecting data on several other indices of senescence for each butterfly (weight, grip strength, flight behaviour) and so could not reasonably also separate each female into an individual laying cage for 24 hours on a biweekly basis. Based on pilot data suggesting that females of both *H. hecale* and *D. iulia* would lay eggs if provided with a host-plant for a 1-hour window, we therefore added a 1-hour egg-laying assay to our battery of biweekly tests. In brief, females were deprived of host-plants for 1.5 days, and then placed individually into small (1m (L) x 0.5m (W) x 2m (H)) egg-laying cages for a 1-hour period with their preferred *Passiflora* host-plant (*P. vitifolia* for *H. hecale* and *P. biflora* for *D. iulia*). After the hour had elapsed, females were removed and eggs were counted.

Unfortunately, this dataset proved to be highly zero-inflated (122 zeroes out of 200 observations), and we ultimately concluded that disorientation related to handling and the new environment made it unlikely for females to lay in the 1-hour assay period. To account for zero-inflation, two-part (hurdle) zero-inflated generalised linear mixed models were fit to the egg count data with a truncated negative binomial distribution [52], using the R package *glmmTMB* v1.1.7 [53]. Hurdle models were selected based on biological reasoning; as daily oviposition rates for *H. hecale* have been found to number around 10 eggs per day [16], and in young *D. iulia*, between 15 and 50 eggs per day [15], it is likely that laying 0 eggs after 1.5 days of host-plant deprivation reflected a different underlying process (for example, stress from handling) than that determining number of available eggs to be laid. We found neither age (χ^2_1 = 1.24, p = 0.265) nor diet (χ^2_1 = 0.29, p = 0.589) to be a significant predictor of egg count in either species. However, we did still recapitulate Dunlap Pianka *et al.*'s finding of higher oviposition in the shorter-lived *D. iulia* (χ^2_1 = 9.98, p = 0.002), which was predicted to lay an estimated 8.11 eggs per day during the assay, more than double the estimated 3.50 eggs per day for *H.*

hecale. Because of the significant limitations of this dataset, and because the main findings are already reported in the literature in specifically-designed studies, we did not include this in the manuscript; however, we are happy to add them as a Supplementary Note if you would prefer.

In summary, it seems that the shift towards pollen-feeding in *Heliconius* is also associated with a reduction in daily oviposition rates, but greater lifetime egg output, in comparison with the non-pollen-feeding outgroups; and there is no evidence for a difference in egg size. However, to the best of our knowledge, no one has yet compared egg viability or any other metrics of reproductive effort between *Heliconius* and any of the non-pollen-feeding Heliconiini. We have updated the Introduction (lines 72-76) to refer to this information.

Similarly, sustained reproductive effort throughout the long lifespan (at least that's what earlier papers showed) is no doubt beneficial, but do certain environmental conditions favour persistence of such a life-history strategy in Heliconius? In other words, is the pollen they feed on from specific species available throughout the year? Moreover, how seasonal are host plants? I am wondering if the plants are not around, and if butterflies cannot lay eggs, there would be a mismatch between the life-history strategy and ecological conditions.

Notably, the pollen-rich *Psiguria* vines, which serve as the primary pollen source for many *Heliconius* species, produce male flowers year round with little seasonal variation [54]. While densities are comparatively low, individual plants are known to produce inflorescences every few days, and can flower continuously for up to a year [55]. Many *Heliconius* species also exploit several other pollen sources, such as *Lantana*, for which there are less precise data on seasonal pollen production. However, a larger study on *Heliconius* pollen collection across several sites, years, and seasons did not find any evidence for a disappearance of pollen sources at any point in the year [56]. All available data suggest that pollen sources are relatively constant.

Though less studied, there does also appear to be seasonal variation in host-plant availability in sub-tropical regions, with some *Passiflora* species showing defoliation or reduced growth at particular times of the year [57, 58]. However, there is again no evidence for a complete absence of host-plants, although reduced foliage may increase competition for the resource. One two-year study investigated seasonality in *Passiflora misera* and *P. suberosa*, both host-plants for *Heliconius erato phyllis*, and found increased defoliation for both *Passiflora* species in autumn and winter, but to a much greater extent in *P. misera* (at its lowest retaining ~10% intact leaves), whereas *P.*

suberosa was available all year-round (at its lowest retaining ~50% intact leaves) [57]. As a result, *H. erato* switched to its non-preferred host-plant, *P. suberosa,* in periods with low availability of *P. misera*. This is consistent with the idea that in areas with temporal cycles of host plant abundances, selection should favour generalist feeding strategies, as put forward by Benson in 1978 [59]. However, this seasonality has mostly only been shown for sub-tropical regions, for example in southeastern Brazil, where the study on *P. misera* and *P. suberosa* took place [57]. It seems that for tropical regions, for example in Trinidad, host-plant availability is instead relatively constant all year round, promoting specialist feeding strategies [59]. Either way, the data once again do not point to an overall absence of host-plants in any particular season. We have updated the manuscript to include reference to the constancy of pollen sources and availability of host-plants (lines 326-329) for any readers who may have similar questions.

I do think the lifespan dataset from butterfly farms is great, but I was hoping to find more details on how the butterflies were kept, in what conditions, and especially for Heliconius, what diet were they fed on? I believe these details are generally important, but also because there is a strong emphasis on a single individual surviving for 348 days.

Thank you for your enthusiasm for this dataset. Reviewer #2 had similar questions, and so we have rectified the lack of detail on conditions in this exhibit in Supplementary Note 5, and included additional information in our response to their comment above. In brief, the dimensions of the exhibit are 30m (L) x 15m (W) x 6m (H), many floral nectar/pollen resources are provided (including Heliconiini favourites such as *Lantana* and *Stachytarpheta*), and these are supplemented with artificial feeders containing a 20% v/v honey solution. Temperatures range between 16-32°C throughout the year.

In their mark-recapture experiment, do the authors know if the females were laying eggs or dissected the females after death to check the ovarioles to assess reproductive activity?

We were unable to dissect the females after death, as it was extremely rare to recover a dead individual before it otherwise decomposed (recovering only 5 dead individuals out of a total of 959 individuals released into the cages). We ensured that all females had the opportunity to lay by keeping track of which species were in the large “mark-release-recapture” cage and placing shoots or pots of their preferred host-plants where they could be easily encountered. We replaced these every few days, and they were

generally covered with eggs, although we had no way to verify that all females from each species were indeed laying on the plants.

How variable were the grip strength measurements, as the authors report that only the maximum value was used in the analyses?

Please see our response to Reviewer #1, who also had questions about this. In brief, we had *a priori* chosen to measure maximum grip strength, as we were interested in maximal performance, and were following the methodological approach of another paper [28]; we have updated the Methods (lines 568-572) to justify this. During the trials, individuals occasionally did not properly "grip" the perch, leading to unrealistically low readings, but we were unconcerned by this due to our decision to use the maximum. Therefore the measurements were indeed quite variable: for *H. hecale*, ranging from 0.34g to 4.63g, with a median of 1.93g and an interquartile range of 1.42–2.45g; and for *D. iulia*, ranging from 0.17g to 3.11g, with a median of 1.21g and an interquartile range of 0.89–1.60g.

Despite this, however, the maximum reading for grip strength was highly correlated with the mean reading across all 5 trials for each biweekly assay, both for *H. hecale* (ρ = 0.92, $p < 0.001$) and for *D. iulia* (ρ = 0.92, $p < 0.001$), as shown in Figure 1. We have now included these details in Supplementary Note 6 for any interested readers. We also updated the models to remove any observations with studentised residuals > 3, which is a common technique for identifying potential outliers. These models returned the same main findings, with reduced grip strength in pollen-deprived *H. hecale* ($F_{1, 73.52} = 4.60$, $p = 0.035$) but not *D. iulia* ($F_{1, 81.76} = 3.24$, $p = 0.076$), and a decline in grip strength with age in *D. iulia* ($F_{1, 99.48} = 11.92$, $p = 0.001$) but not *H. hecale* ($F_{1, 225.11} = 1.65$, $p = 0.201$).

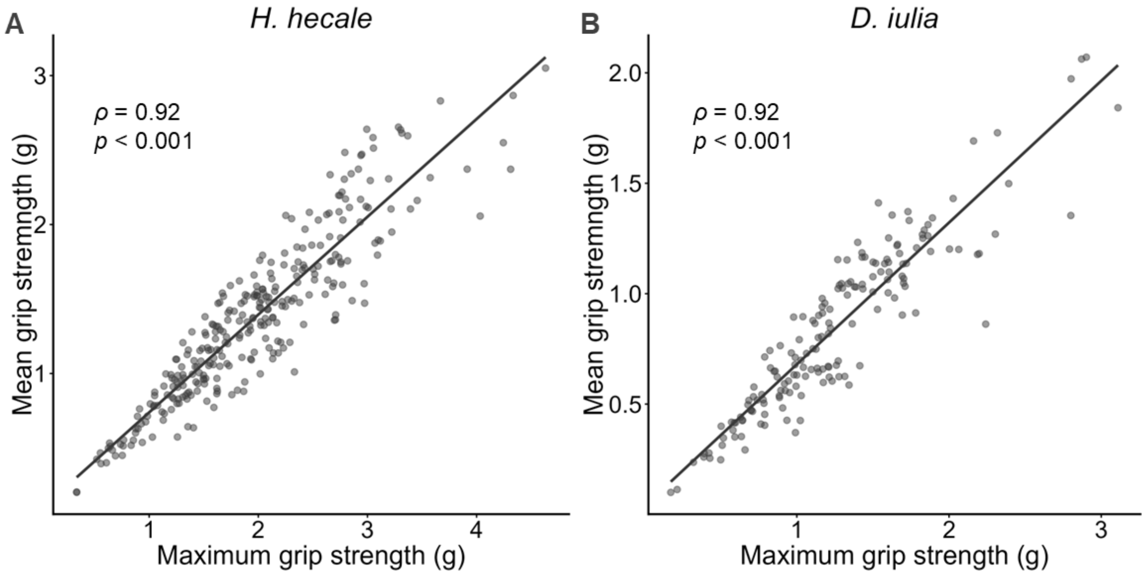

Figure 2

Other comments:

73-76: unclear which are these traits are and which traits are the targets of selection that have having indirect effect on longevity.

Our intention was not to suggest that these derived traits in particular necessarily contributed to *Heliconius'* lengthened lifespan, but rather that this longevity might itself also be a derived trait evolved across evolutionary time, and not an immediate plastic response to a nutritional difference. We have edited these lines to clarify this (lines 80-81).

84-85: Authors say this, but there is a heavy emphasis on a single individual from a single species.

We have now removed the reference to this individual from the abstract and edited the text in our Discussion (lines 237-241) to reduce emphasis. We have also included multiple references to the problematic nature of maximum lifespan as a metric of ageing in an attempt to caution against over-interpretation of our maximum lifespan figures (lines 89-93; 430-433). However, we still point to our *H. hewitsoni* statistic as we believe it helps to cement the case for *Heliconius* as a long-lived butterfly genus to non-expert readers, who will be overwhelmingly familiar with maximum lifespan as compared with

any other ageing metric. We think this is justified considering that in the same paper we temper enthusiasm over such metrics with reference to why they are problematic.

115-117: Is this true? I thought the studies already showed this (e.g. Dunlap-Pianka et al. 1977)

Dunlap-Pianka *et al.* show evidence for a plastic lifespan extension / delayed reproductive senescence in *Heliconius* contingent upon pollen-feeding. Their work suggested that pollen-deprived butterflies showed no evidence for slowed ageing, and were in fact "phenocopies" of their short-lived relatives, implying that *Heliconius*' lifespan was simply a plastic response to enhanced nutrition. Our work shows that *Heliconius'* lifespan extension and slowed ageing are evolved, and persist even in the absence of pollen.

147-148: Did the artificial protein solution mimic that of the pollen?

Critical Care Formula (CCF) (Vetark Professional, Winchester, U.K.) is a protein supplement normally used for rearing birds, reptiles, and small mammals; however, it has been commonly used for years by *Heliconius* biologists as an amino acid supplement, as it dissolves readily in sucrose solution and the butterflies will willingly feed on it with no adverse effects (see [11, 60, 61]; see also https://www.heliconius.org/2009/artificial-feeders/). Compared to individuals fed only on sugar water, female *Heliconius* fed on CCF are shown to have higher levels of cyanogenic glucosides and exhibit a nonsignificant trend towards higher oviposition [20], suggesting that they are able to derive nutritional benefits from its dissolved amino acids. However, it is a proprietary formula, and so we are unsure of precise amino acid concentrations, and did not select it specifically for its similarity to pollen. We would like to clarify once again that this artificial protein solution was only used in the cohort of butterflies from the repurposed cognitive experiment, and that butterflies in the semi-natural mark-release-recapture cohort and the pollen-fed cage of the main pollen-manipulation cohort had access to real flowers.

469-470: I am unsure what it means by 'retreat to higher reaches of the lifespan'

This point is clarified in the following sentence, which we have edited for clarity; "This means that increased reproductive longevity will expose older ages to selection, favouring the evolution of pro-longevity mechanisms"

482-485: The Authors say this, but there is heavy emphasis on a single individual that survived for 348 days

See previous response.

References

1. Chai, P. & Wicksten, M. Adaptive coloration in invertebrates. *Proceedings of a Symposium Sponsored by the American Society of Zoologists* (Texas A & M University, 1990).
2. Williams, G.C. Pleiotropy, natural selection, and the evolution of senescence. *Evolution* **11**, 398 (1957).
3. Abrams, P.A. Does increased mortality favor the evolution of more rapid senescence? *Evolution* **47**, 877-887 (1993).
4. Caswell, H. Extrinsic mortality and the evolution of senescence. *Trends Ecol. Evol.* **22**, 173-174 (2007).
5. Wensink, M.J., Caswell, H. & Baudisch, A. The rarity of survival to old age does not drive the evolution of senescence. *Evol. Biol.* **44**, 5-10 (2017).
6. Moorad, J., Promislow, D. & Silvertown, J. Evolutionary ecology of senescence and a reassessment of Williams' 'extrinsic mortality' hypothesis. *Trends Ecol. Evol.* **34**, 519-530 (2019).
7. De Vries, C., Galipaud, M. & Kokko, H. Extrinsic mortality and senescence: a guide for the perplexed. *Peer Community J.* **3**, (2023).
8. Jiggins, C. The passion: niche differentiation, coexistence, and coevolution. In *The Ecology and Evolution of* Heliconius *Butterflies* (Oxford University Press, 2017).
9. Nahrstedt, A. & Davis, R.H. Biosynthesis and quantitative relationships of the cyanogenic glucosides, linamarin and lotaustralin, in genera of the Heliconiini (Insecta: Lepidoptera). *Comp. Biochem. Physiol. B* **82**, 745-749 (1985).
10. Pinheiro de Castro, É.C. et al. The dynamics of cyanide defences in the life cycle of an aposematic butterfly: biosynthesis versus sequestration. *Insect Biochem. Mol. Biol.* **116**, 103259 (2020).
11. Mattila, A.L.K., Jiggins, C.D. & Saastamoinen, M. Condition dependence in biosynthesized chemical defenses of an aposematic and mimetic *Heliconius* butterfly. *Ecol. Evol.* **12**, (2022).
12. Brower, L.P., Brower, J.V.Z. & Collins, C.T. Experimental studies of mimicry. 7. Relative palatability and Mullerian mimicry among neotropical butterflies of the subfamily Heliconiinae. *Zoologica* **48**, 65-84 (1963).
13. Chouteau, M., Dezeure, J., Sherratt, T.N., Llaurens, V. & Joron, M. Similar predator aversion for natural prey with diverse toxicity levels. *Anim. Behav.* **153**, 49-59 (2019).
14. Pinheiro de Castro, É.C. et al. Sequestration and biosynthesis of cyanogenic glucosides in passion vine butterflies and consequences for the diversification of their host plants. *Ecol. Evol.* **9**, 5079-5093 (2019).
15. Dunlap-Pianka, H., Boggs, C.L. & Gilbert, L.E. Ovarian dynamics in heliconiine butterflies: programmed senescence versus eternal youth. *Science* **197**, 487-490 (1977).

16. Dunlap-Pianka, H.L. Ovarian dynamics in *Heliconius* butterflies: correlations among daily oviposition rates, egg weights, and quantitative aspects of oogenesis. *J. Insect Physiol.* **25**, 741-749 (1979).
17. Boggs, C.L. Nutritional and life-history determinants of resource allocation in holometabolous insects. *Am. Nat.* **117**, 692-709 (1981).
18. O'Brien, D.M., Boggs, C.L. & Fogel, M.L. Pollen feeding in the butterfly *Heliconius charitonia*: isotopic evidence for essential amino acid transfer from pollen to eggs. *Proc. R. Soc. Lond. B* **270**, 2631-2636 (2003).
19. Beck, J. & Fiedler, K. Adult life spans of butterflies (Lepidoptera: Papilionoidea + Hesperioidea): broadscale contingencies with adult and larval traits in multi-species comparisons. *Biol. J. Linn. Soc.* **96**, 166-184 (2009).
20. Pinheiro De Castro, E.C. et al. Pollen-feeding delays reproductive senescence and maintains toxicity of *Heliconius erato*. *Peer Community J.* **5**, (2025).
21. Cardoso, M.Z. & Gilbert, L.E. Pollen feeding, resource allocation and the evolution of chemical defence in passion vine butterflies. *J. Evol. Biol.* **26**, 1254-1260 (2013).
22. Montejo-Kovacevich, G. et al. Altitude and life-history shape the evolution of Heliconius wings. *Evolution* **73**, 2436-2450 (2019).
23. Salguero-Gómez, R. More social species live longer, have longer generation times and longer reproductive windows. *Philos. Trans. R. Soc. B* **379**, (2024).
24. Beltrán, M. et al. Do pollen feeding, pupal-mating and larval gregariousness have a single origin in *Heliconius* butterflies? Inferences from multilocus DNA sequence data. *Biol. J. Linn. Soc.* **92**, 221-239 (2007).
25. Kozak, K.M. et al. Multilocus species trees show the recent adaptive radiation of the mimetic Heliconius butterflies. *Syst. Biol.* **64**, 505-524 (2015).
26. Merrill, R.M. et al. The diversification of *Heliconius* butterflies: what have we learned in 150 years? *J. Evol. Biol.* **28**, 1417-1438 (2015).
27. Cicconardi, F. et al. Evolutionary dynamics of genome size and content during the adaptive radiation of Heliconiini butterflies. *Nat. Commun.* **14**, (2023).
28. Davis, A.K., Smith, F.M. & Ballew, A.M. A poor substitute for the real thing: captive-reared monarch butterflies are weaker, paler and have less elongated wings than wild migrants. *Biol. Lett.* **16**, 20190922 (2020).
29. Kubinec, R. Ordered beta regression: a parsimonious, well-fitting model for continuous data with lower and upper bounds. *Polit. Anal.* **30**, 1-18 (2022).
30. Borrero, J., Rivas-Sánchez, D., Wright, D.S., Bacquet, C.N., Montgomery, S.H., Keller, A. & Merrill, R.M. Sexual dimorphism in pollen foraging and sensory traits in *Heliconius* butterflies. Preprint at https://doi.org/10.1101/2025.09.18.676771 (2025).
31. Boggs, C.L. & Ross, C.L. The effect of adult food limitation on life history traits in *Speyeria mormonia* (Lepidoptera: Nymphalidae). *Ecology* **74**, 433-441 (1993).
32. Colchero, F., Jones, O.R. & Rebke, M. BaSTA: an R package for Bayesian estimation of age-specific survival from incomplete mark-recapture/recovery data with covariates. *Methods Ecol. Evol.* **3**, 466-470 (2012).

33. DeVries, P.J. *The Butterflies of Costa Rica and Their Natural History. Vol I: Papilionidae, Pieridae, Nymphalidae* (Princeton University Press, 1987).
34. Rijn, P.C.J. van, Kooijman, J. & Wäckers, F.L. The impact of floral resources on syrphid performance and cabbage aphid biological control. *Proceedings of the Landscape Management for Functional Biodiversity 2nd Working Group Meeting*. (Zürich-Reckenholz, 2006).
35. van Rijn, P.C.J., Kooijman, J. & Wäckers, F.L. The contribution of floral resources and honeydew to the performance of predatory hoverflies (Diptera: Syrphidae). *Biol. Control* **67**, 32-38 (2013).
36. Leius, K. Effects of pollens on fecundity and longevity of adult *Scambus buolianae* (Htg.) (Hymenoptera: Ichneumonidae). *Can. Entomol.* **95**, 202-207 (1963).
37. Irvin, N.A., Wratten, S.D., Frampton, C.M., Bowie, M.H., Evans, A.M. & Moar, N.T. The phenology and pollen feeding of three hover fly (Diptera: Syrphidae) species in Canterbury, New Zealand. *N. Z. J. Zool.* **26**, 105-115 (1999).
38. Laubertie, E.A., Wratten, S.D. & Hemptinne, J.-L. The contribution of potential beneficial insectary plant species to adult hoverfly (Diptera: Syrphidae) fitness. *Biol. Control* **61**, 1-6 (2012).
39. Luo, S., Li, Y., Chen, S., Zhang, D. & Renner, S.S. Gelechiidae moths are capable of chemically dissolving the pollen of their host plants: first documented sporopollenin breakdown by an animal. *PLoS ONE* **6**, e19219 (2011).
40. Soto, S.S. & Nakano, O. Biología de *Batrachedra nuciferae* Hodges (Lepidoptera: Coleophoridae), plaga del cocotero en América del Sur. *Bol. Mus. Entomol. Univ. Valle* **9**, 22-26 (2008).
41. Cock, M.J.W. *Batrachedra nuciferae*, an inflorescence-feeding moth associated with coconut, *Cocos nucifera*, and palmiste, *Roystonea oleracea*, in Trinidad, West Indies. *J. Insect Sci.* **13**, 124 (2013).
42. Gilbert, F.S. Foraging ecology of hoverflies: morphology of the mouthparts in relation to feeding on nectar and pollen in some common urban species. *Ecol. Entomol.* **6**, 245-262 (1981).
43. Pinheiro, L.A., Torres, L.M., Raimundo, J. & Santos, S.A.P. Effects of pollen, sugars and honeydew on lifespan and nutrient levels of *Episyrphus balteatus*. *BioControl* **60**, 47-57 (2015).
44. Hunt, J.H., Brown, P.A., Sago, K.M. & Kerker, J.A. Vespid wasps eat pollen (Hymenoptera: Vespidae). *J. Kans. Entomol. Soc.* **64**, 127-130 (1991).
45. Toth, A.L., Sumner, S. & Jeanne, R.L. Patterns of longevity across a sociality gradient in vespid wasps. *Curr. Opin. Insect Sci.* **16**, 28-35 (2016).
46. Southon, R.J., Bell, E.F., Graystock, P. & Sumner, S. Long live the wasp: adult longevity in captive colonies of the eusocial paper wasp *Polistes canadensis* (L.). *PeerJ* **3**, e848 (2015).
47. Korb, J. Why do social insect queens live so long? Approaches to unravel the sociality-aging puzzle. *Curr. Opin. Insect Sci.* **16**, 104-107 (2016).

48. Jervis, M. Functional and evolutionary aspects of mouthpart structure in parasitoid wasps. *Biol. J. Linn. Soc.* **63**, 461-493 (1998).
49. Picciau, L., Alma, A. & Ferracini, C. Effect of different feeding sources on lifespan and fecundity in the biocontrol agent *Torymus sinensis*. *Biol. Control* **134**, 45-52 (2019).
50. Hebberecht, L., Melo-Flórez, L., Young, F.J., Mcmillan, W.O. & Montgomery, S.H. The evolution of adult pollen feeding did not alter postembryonic growth in *Heliconius* butterflies. *Ecol. Evol.* **12**, (2022).
51. Brown, K.S. The biology of *Heliconius* and related genera. *Annu. Rev. Entomol.* **26**, 427-457 (1981).
52. Zuur, A.F., Ieno, E.N., Walker, N.J., Saveliev, A.A. & Smith, G.M. *Mixed Effects Models and Extensions in Ecology with R* (Springer, 2009).
53. Brooks, M.E. et al. glmmTMB balances speed and flexibility among packages for zero-inflated generalized linear mixed modeling. *R J.* **9**, 378-400 (2017).
54. Murawski, D.A. Floral resource variation, pollinator response, and potential pollen flow in *Psiguria warscewiczii*. *Ecology* **68**, 1273-1282 (1987).
55. Gilbert, L.E. Ecological consequences of a coevolved mutualism between butterflies and plants. In *Coevolution of Animals and Plants* (eds. Gilbert, L.E. & Raven, P.H.) 210-240 (University of Texas Press, 1975).
56. Boggs, C.L., Smiley, J.T. & Gilbert, L.E. Patterns of pollen exploitation by *Heliconius* butterflies. *Oecologia* **48**, 284-289 (1981).
57. Rodrigues, D. & Moreira, G.R.P. Seasonal variation in larval host plants and consequences for *Heliconius erato* (Lepidoptera: Nymphalidae) adult body size. *Austral Ecol.* **29**, 437-445 (2004).
58. Benson, W.W., Brown, K.S. & Gilbert, L.E. Coevolution of plants and herbivores: passion flower butterflies. *Evolution* **29**, 659 (1975).
59. Benson, W.W. Resource partitioning in passion vine butterflies. *Evolution* **32**, 493-518 (1978).
60. Darragh, K. et al. Male sex pheromone components in *Heliconius* butterflies released by the androconia affect female choice. *PeerJ* **5**, e3953 (2017).
61. Harpel, D., Cullen, D.A., Ott, S.R., Jiggins, C.D. & Walters, J.R. Pollen feeding proteomics: salivary proteins of the passion flower butterfly, *Heliconius melpomene*. *Insect Biochem. Mol. Biol.* **63**, 7-13 (2015).

REVIEWERS' COMMENTS

Reviewer #1 (Remarks to the Author):

I think authors have responded thoroughly and constructively to my earlier comments. My main original concern was that the inference linking slowed senescence and extended lifespan in Heliconius to pollen feeding did not sufficiently consider alternative explanations, notably aposematism/palatability. The authors have now added some discussion of this issue, including relevant theory on extrinsic mortality and senescence, and have moderated some of their language. The authors have also responded well to some methodological and analytical queries. In particular, they have clarified the design of the diet experiment, provided additional justification and sensitivity analyses for the ageing parameters and grip-strength metrics, clarified modelling decisions, and updated the literature survey. These revisions increase confidence in the robustness of the results and improve transparency for readers. One limitation that remains inherent to the design is that the diet manipulation was conducted at the cage level, but I note that the fact that the different species shared a cage and the experiment was conducted in batches over months. Overall, I believe the manuscript is a valuable and comprehensive contribution to the study of lifespan and senescence in insects and will be of broad interest.

We thank you for your helpful comments, which have served to greatly improve the paper.

Reviewer #2 (Remarks to the Author):

Thank you for your hard work on the revision! To me it looks like all comments and concerns have been answered and the manuscript now reads exceptionally well. Despite my best efforts, I was unable to find even a single typo.

The only comment I have has to do with the added sentence on liness 237-240: The maximum reported lifespan of 348 days for Heliconius hewitsoni also exceeds any butterfly previously recorded in the scientific literature, and is within range of Myscelia cyaniris (380 days), which we now present as the longest-lived butterfly species to date based on data from butterfly exhibitors (Supplementary Data 1).

-- The wording is a bit confusing, as the non-Heliconius Myscelia cyaniris is mentioned here for the first time and its relationship with Heliconius hewitsoni isn't clear. I'd suggest splitting the sentence in two to make it clearer to understand.

We thank you for your positive comments, which have been very helpful in improving and refining the paper. We have now edited lines 237-241 to introduce *Myscelia cyaniris* more clearly, splitting the sentence in two and stating that this species is a non-Heliconius nymphalid.
